# Polygenic plague resistance in the great gerbil uncovered by population sequencing

**DOI:** 10.1093/pnasnexus/pgac211

**Published:** 2022-10-05

**Authors:** Pernille Nilsson, Mark Ravinet, Yujun Cui, Paul R Berg, Yujiang Zhang, Rong Guo, Tao Luo, Yajun Song, Emiliano Trucchi, Siv N K Hoff, Ruichen Lv, Boris V Schmid, W Ryan Easterday, Kjetill S Jakobsen, Nils Chr Stenseth, Ruifu Yang, Sissel Jentoft

**Affiliations:** Centre for Ecological and Evolutionary Synthesis, Department of Biosciences, University of Oslo, 0371 Oslo, Norway; Centre for Ecological and Evolutionary Synthesis, Department of Biosciences, University of Oslo, 0371 Oslo, Norway; School of Life Sciences, University of Nottingham, NG9 8DQ, UK; State Key Laboratory of Pathogen and Biosecurity, Beijing Institute of Microbiology and Epidemiology, Beijing 100071, China; Centre for Ecological and Evolutionary Synthesis, Department of Biosciences, University of Oslo, 0371 Oslo, Norway; Centre for Coastal Research, Department of Natural Sciences, University of Agder, 4604 Kristiansand, Norway; Xinjiang Center for Disease Control and Prevention, Urumqi 830002, China; Xinjiang Center for Disease Control and Prevention, Urumqi 830002, China; Xinjiang Center for Disease Control and Prevention, Urumqi 830002, China; State Key Laboratory of Pathogen and Biosecurity, Beijing Institute of Microbiology and Epidemiology, Beijing 100071, China; Department of Life and Environmental Sciences, Marche Polytechnic University, Via Brecce Bianche, 60131 Ancona, Italy; Centre for Ecological and Evolutionary Synthesis, Department of Biosciences, University of Oslo, 0371 Oslo, Norway; State Key Laboratory of Pathogen and Biosecurity, Beijing Institute of Microbiology and Epidemiology, Beijing 100071, China; Centre for Ecological and Evolutionary Synthesis, Department of Biosciences, University of Oslo, 0371 Oslo, Norway; Centre for Ecological and Evolutionary Synthesis, Department of Biosciences, University of Oslo, 0371 Oslo, Norway; Centre for Ecological and Evolutionary Synthesis, Department of Biosciences, University of Oslo, 0371 Oslo, Norway; Centre for Ecological and Evolutionary Synthesis, Department of Biosciences, University of Oslo, 0371 Oslo, Norway; Ministry of Education Key Laboratory for Earth System Modeling, Department of Earth System Science, Tsinghua University, Beijing 100084, China; State Key Laboratory of Pathogen and Biosecurity, Beijing Institute of Microbiology and Epidemiology, Beijing 100071, China; Centre for Ecological and Evolutionary Synthesis, Department of Biosciences, University of Oslo, 0371 Oslo, Norway

**Keywords:** great gerbil, population genomics, polygenic resistance, plague, challenge experiment

## Abstract

Pathogens can elicit high selective pressure on hosts, potentially altering genetic diversity over short evolutionary timescales. Intraspecific variation in immune response is observable as variable survivability from specific infections. The great gerbil (*Rhombomys opimus*) is a rodent plague host with a heterogenic but highly resistant phenotype. Here, we investigate the genomic basis for plague-resistant phenotypes by exposing wild-caught great gerbils to plague (*Yersinia pestis*). Whole genome sequencing of 10 survivors and 10 moribund individuals revealed a subset of genomic regions showing elevated differentiation. Gene ontology analysis of candidate genes in these regions demonstrated enrichment of genes directly involved in immune functions, cellular metabolism and the regulation of apoptosis as well as pathways involved in transcription, translation, and gene regulation. Transcriptomic analysis revealed that the early activated great gerbil immune response to plague consisted of classical components of the innate immune system. Our approach combining challenge experiments with transcriptomics and population level sequencing, provides new insight into the genetic background of plague-resistance and confirms its complex nature, most likely involving multiple genes and pathways of both the immune system and regulation of basic cellular functions.

Significance Statement The great gerbil (*Rhombomys opimus*) is identified as the key rodent plague host in Central Asia. Studies have shown that it is largely resistant to plague; however, individual heterogeneity is observed. In this paper, we employ whole genome sequencing and transcriptomics on a set of wild-caught great gerbil individuals experimentally infected with plague (*Yersinia pestis*) and identify genetic differences (genome-wide) between gerbils that survived the infection and those that succumbed to it. The whole genome approach allowed identification of potential candidate genes involved in the remarkable plague resistance that characterizes this species.

## Introduction

Pathogens are well recognized as one of the strongest selective agents influencing host population genomic diversity through adaptation (1–3). Differences in pathogen pressures within and between host populations can reduce or increase genetic diversity, depending on the lethality of the disease and the type of selection. For instance, highly virulent pathogens and purifying selection at resistance loci generally act to reduce genetic diversity (4, 5), while balancing selection and low and intermediate virulence can result in higher genetic diversity (6, 7). The underlying genomic basis for host resistance to infectious diseases is predominantly caused by polygenic traits, where many genes or variants collectively contribute to the phenotype (8–10). For instance, resistance to tuberculosis in humans has been linked to multiple genomic loci (11). Thus, selection for pathogen resistance is likely to shape diversity at multiple regions in the genome.

The great gerbil (*Rhombomys opimus*) is a well-studied wild, social rodent and serves as reservoir for the plague bacterium (*Yersinia pestis*) (12, 13). Knowledge of their high level of plague resistance has existed since the early 1950s and individual heterogeneity in response to infection has been revealed by challenge experiments (14, 15). Heterogenic response to plague infection is generally considered a prerequisite for permanent plague reservoirs and has also been reported within populations of other rodent plague hosts like the black rat (*Rattus rattus*) and Asian house shrew (*Suncus murinus*) in Madagascar (16–19). For plague to persist for long times on a small local scale, there has to be a balance between having enough rodents experiencing high-level, but often fatal, bacteremia and enough rodents that persist to avoid local extinction (20, 21). One way to achieve such a balance is through heterogenic response to plague infection. Several studies of laboratory and wild rodent species have been used to investigate the genetic basis for plague resistance, and current evidence strongly suggests that it is likely governed by multiple genes (22–24). Although some of these studies were unable to determine candidate genes due to the low resolution of their method (23), others identified multiple candidate genes involved in immune-related processes as well as genes lacking a known immune function (22, 25, 26). Both Tollenaere et al. (2012b) and Blanchet et al. (2010) highlights receptors of interleukin 17 (IL17) as potential important factors in plague resistance in black rats and SEG mice, respectively (22, 26). In general, our understanding of the genomic basis for plague susceptibility and resistance is still poor and the exact mechanism might differ between species (27–30).

Host–pathogen coevolution results in resistance phenotypes on both sides as a consequence of natural selection for adaptation and counter-adaptation (31). *Yersinia pestis* is a relatively new pathogen viewed on an evolutionary timescale and displays distinct eco-geographical variations in vector–host transmission modes. For example, it has been demonstrated that in a particular site in China (Guertu) *Y. pestis* transmission is enhanced by bacterial gene selection-directed biofilm formation in the flea (extended phenotype changes) caused by climate fluctuations (32). Based on current phylogenetic analysis of *Y. pestis*, it seems plausible that the bacterium gradually evolved from *Y. pseudotuberculosis* into its more lethal, flea-borne form approximately 5 to 6000 y ago, in Central Asia or western China (33–36).

**Fig. 1.**
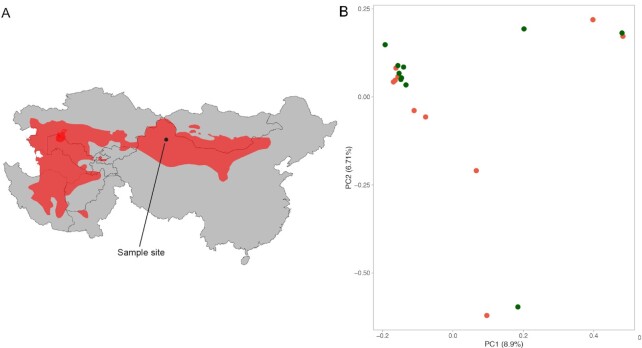
Gerbil distribution and population structure at the Xinjiang sampling site. (A) Distribution of gerbils in Central Asia, ranging from the Caspian Sea in the west to deep within China to the east. The sampling site in Xinjiang, China is marked by a black point and line. (B) Principal component analysis (PCA) of high-quality, linkage disequilibrium (LD)-pruned SNPs does not separate according to disease outcome. Dark green circles represent survivors and dark orange circles depict moribund individuals.

The Gurbantünggüt desert of the Junggar Basin in northwest China is one of the most recently identified plague foci and constitutes one of the more eastern parts of the great gerbil distribution (Fig. 1A) (37). The first occurrences of plague in wildlife in this area were reported in 2005 (37). Plague has been known to reappear in areas after decades of quiescence (38, 39). As such, plague might have been present in the rodent population prior to initiation of surveillance in the 1950s (40), with ample time for co-evolution. However, if plague indeed only entered the system in 2005, the signatures of an arms race between *Y. pestis* and gerbil could still be detectable in the host due to the short generation times and large population sizes of this animal group (41). Furthermore, the strong selective pressure exerted by a highly virulent pathogen could lead to rapid evolution of host adaptation and resistance to infection. Such rapid evolution to pathogens has been reported in other host populations including wild finches in North America and European rabbits in Australia (42–45).

Advances in high-throughput sequencing technology over the past decades have resulted in a burst of detailed investigations of genomic adaptation in nonmodel species (46–49). Recently, Nilsson and co-workers generated a highly contiguous reference genome assembly of the great gerbil (50). The study revealed a species-specific duplication of a Major Histocompatibility Complex class II (*MHCII*) gene with a predicted high affinity for *Yersinia* epitopes (50). Here, we aimed at further investigating the genetic basis of disease resistance in great gerbils by whole genome sequencing wild captured specimens from a location in the plague focus of the Gurbantünggut desert in northwest China. Our study approach is based on the presence of a heterogenic response within the population, i.e. that some individuals are plague-resistant while others are highly susceptible and die after plague exposure, and thus, mirrored in their genomic signatures as a result of natural selection in the recent evolutionary history of the great gerbil. To identify individuals with either of the two phenotypes, i.e. those that either (i) survive or those that were (ii) moribund after infection, for subsequent whole genome sequencing (12× coverage), we conducted a 22-day plague exposure experiment and screened for the two phenotypic outcomes of the infection. By comparing the genomes of moribund vs. surviving gerbils, we sought to identify genetic differences between these two phenotypes. This data set combined with transcriptome sequencing enabled us to investigate potential adaptive effects on the host by a highly virulent pathogen and locate genomic differences that potentially underlie variation in host response to infection.

## Results

### Plague challenge experiment

After plague infection, 21 of 45 challenged gerbils (47%) were deemed moribund and euthanized, all except one during the first 5 days post-infection (p.i) (Table S1). The majority of gerbils responded quickly to the disease with the onset of disease symptoms on day one p.i. For 10 individuals, the symptoms appeared on day two p.i., while five gerbils did not show any signs of disease prior to sacrifice. The surviving animals recovered quickly from the infection after being sick for only a few days, and always less than a week. Of the 21 moribund gerbils, 17 were females while the majority of the surviving individuals were male (14 out of 19), yet all the surviving gerbils with no symptoms, were female. The results of the *q*-square test indicate a sex-bias with females more likely dying of the infection and/or not being visibly sick (*X*-squared = 16.157, *df* = 2, *P*-value = 0.00031) (Table S2).

No significant correlation between the response groups (moribund vs. surviving individuals) and body weight were observed (see Fig. S1), indicating that dosage effect due to differences in body weight is negligible. Additionally, no significant differences between the cages on the individual response were observed, i.e. there were no need for taking any cage effect into account, thus, the number of replicates for the experimental design equals the number of individuals used (*n*  = 45). A subset of individuals were further selected for whole genome sequencing with the overall goal to identify immunogenomic differentiation as well as differential expressed genes in moribund vs. surviving individuals.

### Population structure and demographics

We whole genome sequenced 20 specimens from the moribund (*n *= 10) and surviving (*n *= 10) groups at an average 12× coverage per individual (Table 1). Furthermore, eight individuals from each group were RNA sequenced (liver), of which all were included in the population genome sequencing listed in Table 1, except one survivor. After mapping to the repeat-masked reference genome, calling and filtering variants, we retained a set of 1,120,260 high-quality SNPs and used a subset of 32,816 LD-pruned biallelic SNPs for nonparametric (PCA) inference of population structure. The individuals do not separate based on outcome of the challenge experiment, but some separation is seen along the first principal component (8.9% PVE), with the majority of individuals from both groups forming a relatively tight cluster (Fig. 1B). A small number of individuals are also separated from the rest along the second principal component (6.71% PVE) and consists of four moribund individuals and one survivor (Fig. 1B). Estimation of relatedness [by calculating identity by descent (IBD)], using the LD-pruned SNP set, revealed some degree of relatedness between several of the individuals. This matched the clustering pattern seen in the PCA where they form more spread out groups separated from the main cluster on both PC1 and PC2 (Fig. S2; Table S3). These IBD calculations suggest that the individuals clustering in the upper right corner might be a mix of first- and second-degree relatives and also suggest a first-degree relation for the two individuals at the bottom of the PCA plot (Table S3).

**Table 1.** Metadata on the 20 DNA-sequenced gerbils used for population analyses.

**Sample name**	**Sex**	**Body weight (*g*)**	**Day of onset of disease (p.i.)**	**Day of recovery (p.i.)**	**Day of sampling (p.i.)**	**Animal status at sampling**
D2-1^a^	Male	176	1		5	Moribund
D2-3	Female	187	1		3	Moribund
D2-4^a^	Male	176	1		5	Moribund
D2-5^a^	Male	224	1		5	Moribund
D4-3^a^	Female	132	2		5	Moribund
D5-1^a^	Female	144	1		4	Moribund
D8-3^a^	Female	123	1		5	Moribund
D9-5	Female	126	1		5	Moribund
D10-3^a^	Male	214	1		3	Moribund
D11-5^a^	Female	145	1		4	Moribund
S3-3^a^	Male	173	2	5	22	Recovered
S5-3	Male	161	2	6	22	Recovered
S6-4^a^	Female	141	1	7	22	Recovered
S8-1^a^	Female	126	1	4	22	Recovered
S9-1^a^	Male	202	1	6	22	Recovered
S9-3	Male	111	1	5	22	Recovered
S10-4^a^	Female	116	2	6	22	Recovered
S11-1^a^	Male	133	1	5	22	Recovered
S5-2^a^	Female	167	-	-	3	Healthy
S10-5	Female	118	-	-	3	Healthy

a RNA sequencing performed for individuals.

Details on sex, body weight, and days of disease onset, recovery, and sampling are listed. Those individuals who also have RNA sequenced are marked. The animals’ status at sampling is separated into those who died of the infection (“Moribund”) and those that survived (“Recovered”). Individuals denoted as “Healthy” had no clinical symptoms observed. Sample names are composed of outcome (Dead or Survived, animal group, and animal number in that group).

Genome-wide estimates of mean *F*_ST_ and nucleotide diversity (π) revealed that both the relative differentiation between moribund and survivors and the (overall) nucleotide diversity are extremely low (*F*_ST_ = -0.0084 ± 0.0578, π = 0.000152 ± 0.000136). Low nucleotide diversity might have been affected by the general decline in effective population size seen for the population over the last 200Ky. From around 10Ky, there is a strong decline which reached a minimum around 4 to 5Ky ago followed by a recovery around 1 to 2Ky ago (Fig. S3). For a more detailed description and discussion, see Note S1).

### Signatures of selective sweeps

**Fig. 2.**
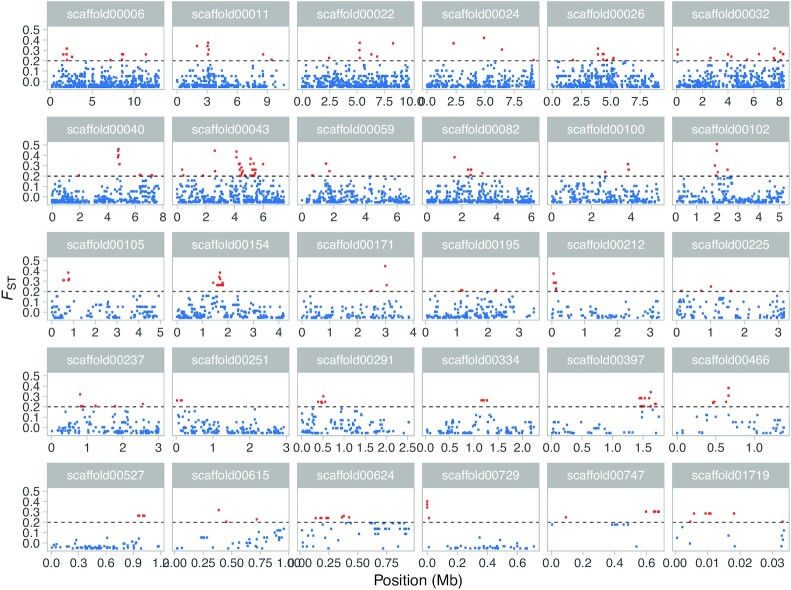
*F*_ST_ patterns for the 30 scaffolds containing elevated *F*_ST_ values. Plots of pairwise *F*_ST_ along the scaffolds shows clear peaks of differentiation (elevated *F*_ST_), with particularly large peaks on scaffolds 22, 40, and 102 and an extended peak on scaffold00043. Horizontal dashed line represents the threshold of high *F*_ST_ values for outlier SNPs (*F*_ST_ > 0.2), also indicated by red points. The data have been subsampled to 0.3 using the sample_frac() command of the dplyr R package for visualization purposes only.

We found signs of recent positive selection identifying 234 iHS (integrated haplotype score) and 122 xpEHH (cross-population haplotype homozygosity) outlier peaks from across the phased genome at a set threshold for significance (log_10_ 1 × 10^−6^) (Tables S4 and S5; Fig. S4). This is a conservative threshold representing a low false discovery rate of 0.001 and hence, only identifies SNPs with the strongest signatures of apparent selection. Sensitivity analysis performed on the scaffold with the highest xpEHH peak (scaffold00080; Table S5) where one individual at the time was excluded from the calculations, demonstrated that the signal was robust (Fig. S5). Simulations showed that under neutrality, the mean differentiation (measured as *F*_ST_) among survivors and moribund individuals was 0, whereas the mean 95th percentile is 0.11 (Fig. S6). Simulating an adaptive allele increased mean *F*_ST_ to 0.017 and the mean 95th percentile to 0.22 (Fig. S6). Therefore, our cut-off of 0.2 is a more conservative value given the null distribution and is appropriate for identifying genome regions putatively under selection. Searching for such regions with high differentiation between moribund gerbils and survivors (*F*_ST_ > 0.2) in 50-SNP windows revealed 30 scaffolds containing areas with elevated mean *F*_ST_ (0.0059 ± 0.077). Several of these scaffolds contain very high values of differentiation upwards of *F*_ST_ = 0.57. For one scaffold (scaffold00043), high *F_ST_* windows span several Mb (Fig. 2; Table S6). In total, we identified 2,706 SNPs with elevated *F*_ST_ values (0.24% of 1,120,260 total SNPs analyzed) on these 30 scaffolds. We took one of the scaffolds with highest *F*_*ST*_ values (scaffold00102; *F*_ST_ = 0.57 at positions 1,971,662, 1,972,081, and 1,980,357) and repeated the analysis multiple times, leaving out one individual each time, to ensure the signal was not dependent on the inclusion of any one individual and to account for the relatedness detected in our data (Fig. S7). PCAs generated for these 30 scaffolds did not reveal any separation between moribund and survivors; however, when extracting only SNPs from the regions of high *F*_ST_, there is moderate to strong separation between moribund and survivors in the PCA plots for several scaffolds (Fig. S8). The groups display near complete separation along PC1 for scaffolds 22 (45.89% PVE), 43 (64.17% PVE), 59 (72.07% PVE), and 102 (51.65% PVE) (Fig. S7), which is reflected in the DAPC and compoplots (Fig. S9).

### Candidate genes and gene ontology (GO)

We identified 904 unique genes within 250 kb on either side of the 234 iHS outlier peaks from across the phased genome (scaffolds > 1 Mb). GO analysis identified 36 enriched gene pathways among the outlier gene set (Table S7). These included pathways involved in regulation of gene expression and protein translation, cellular metabolism and intracellular transport and regulation of cell death (Table S7).

**Fig. 3.**
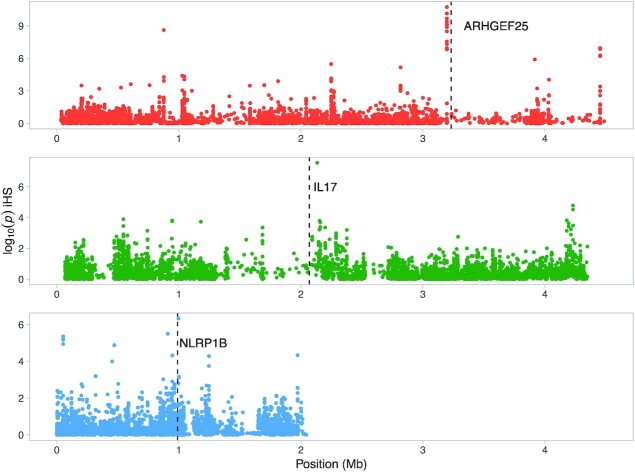
Signatures of recent selection associated with candidate genes on scaffolds 144, 148, and 346. The plot show log_10_ of the iHS analysis *P*-value, where high values indicate strong signals of selection (threshold of significance set at log_10_ 1 × 10^−6^). The scaffold numbers are displayed from top to bottom: scaffold00144, scaffold00148, and scaffold00346 and the vertical dashed lines indicates the location of candidate genes ARHGEF25 (gene start), IL17A (gene midpoint), and NLRP1B (gene midpoint).

Some of the genes associated with significant iHS peaks are well investigated immune genes, like the gene encoding the proinflammatory cytokine *IL17A* (Interleukin 17A) and *NLRP1B* (NLR Family Pyrin Domain Containing 1), which is involved in innate immunity and inflammation as a component of the NLR1 inflammasome (51, 52) (Fig. 3). Moreover, several of the significant iHS peaks were found within genes (*n* = 18), including NLRP1B (Table S8). One of the top 10 most significant iHS peaks is located on scaffold00144 just upstream (34.3 kb) of the *ARHGEF25* (Rho guanine nucleotide exchange factor 25) gene (Fig. 3). This gene works as a guanine nucleotide exchange factor for Rho family of small GTPases (53).

We conducted two complementary genome scans aiming at locating differences between moribund and surviving gerbils, i.e. the xpEHH and *F*_ST_ pairwise analyses. From the xpEHH analysis, we identified 385 unique genes within 250 kb on either side of the 122 outlier peaks across the phased genome (scaffolds > 1 Mb). GO analysis yielded 52 enriched pathways among the gene outlier set and included several immune related pathways (Table S9). In addition, pathways involved in chromatin structure and gene regulation, transcription, and translation were also identified, as well as pathways involved in cytoskeletal dynamics and apoptosis (Table S9). We further identified 565 unique genes falling within 250 kb on either side of the elevated *F*_ST_ regions (*F*_ST_ > 0.2) on the 30 identified scaffolds with higher divergence. GO analysis revealed 11 enriched pathways among the gene set and again included pathways involved in innate immunity, intracellular transport, and translation (Table S10). For 32 of the 565 genes, we identified one or more significantly elevated SNPs located within their annotated boundaries (Table S8).

**Fig. 4.**
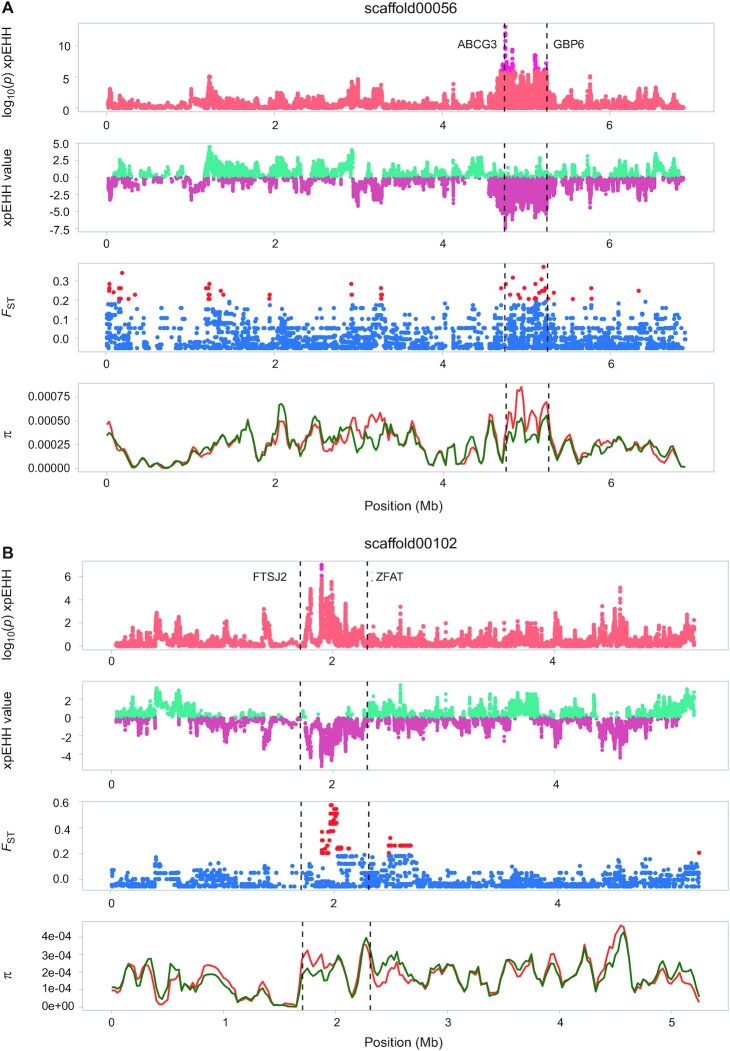
Signatures of recent selection, differentiation, and nucleotide diversity along scaffolds 56 (A) and 102 (B). For each: top panel—plot of log10 (*P*) xpEHH, where high values indicate strong signals of selection (threshold of significance set at log_10_ 1 × 10^−6^) with outlier SNPs in hot pink; second panel: plot of xpEHH value where positive values (teal) indicate selection has occurred in surviving gerbils, while negative values (dark violet) indicate selection in dying gerbils; third panel: relative differentiation (*F*_ST_) between moribund and survivors (red = SNPs with elevated values, *F*_ST _> 0.2); and fourth panel—nucleotide diversity (π) of moribund (orange) and survivors (green). Vertical dashed lines represent in (A) the location of genes *ABCG3* (ATP-binding cassette subfamily G member 3) and *GBP6* (Guanylate-binding protein 6), in (B) the location of genes *FTSJ2* also known as MRM2 (mitochondrial rRNA methyltransferase 2) and *ZFAT* (zinc finger and AT-hook domain containing).

An extracted number of the GO terms of the candidate genes identified by *F*_ST_ and xpEHH peaks are listed in Tables S9 and 10. Of the total 950 genes identified in the xpEHH and *F*_ST_ analyses, only 24 candidate genes were identified by both analyses (Table S11). In Fig. 4A and B, we display xpEHH and *F*_ST_ (as well as nucleotide diversity) for some of the most promising candidate genes identified, where peaks were identified within and/or in proximity with the genes ABCG3 (overlap peak), GBP6 (10 kb downstream), FTSJ (185 kb upstream), and ZFAT (177 kb downstream). Full gene names can be found in the legend of Fig. 4. Other selected candidate genes are shown in Fig. S10A to D. One of these genes is the *VDAC1* (Voltage dependent anion channel 1) gene, which is associated with an *F*_ST_ peak identified on scaffold00043, as well as signatures for selection in the surviving gerbils in xpEHH and nucleotide diversity analyses (Fig. S10A). A more thorough description of the candidate genes is found in the Supplementary Material (Note S2).

### Gene expression during infection

Eight individuals from each group (eight moribund and eight surviving, *n *= 16, seven males and nine females) were RNA sequenced, comprising 15 of the DNA sequenced individuals and one additional individual (Tables 1 and S1). The average number of RNAseq reads going into the mapping was 100 million per individual and the mean total unique mapping against the reference genome was high (79.52%) with a low degree of multimapping (1.3%). Differential expression (DE) analysis between moribund and surviving animals with edgeR resulted in 146 significantly DE expressed genes (FDR *P* < 0.05) (Table S12). GO analyses revealed 22 significantly enriched pathways in the full gene set and showed that the gerbil immune system is highly activated during infection with plague. Several prominent inflammatory pathways are enriched and include both cellular and humoral immunity (Table S13). Of the 146 DE genes, 124 were upregulated and 22 were downregulated in moribund individuals (Table S12). Most of the enriched pathways reported for all 146 genes are those reported for just the upregulated genes (Table S14). Three significantly enriched pathways were reported for the 22 downregulated genes (Table S15).

Twenty-two of the genes identified in the DE analysis overlapped with candidate genes identified in one or more of the genome-wide scans for recent positive selection (iHS and xpEHH) and differentiation (*F*_ST_) (Table S16). For instance, the genes *PSD4* (PH and SEC7 domain-containing protein 4) and *SERPINA1* (Serpin Family A Member 1) were found to be upregulated in moribund gerbils while two ribosomal genes *RPL27* and *RPSA* were downregulated.

## Discussion

Whole genome sequencing of wild gerbils that either survived or succumbed to a plague challenge experiment showed no overall genomic divergence between infection response phenotypes. However, smaller genomic regions with high differentiation between survivors and moribund individuals reveal evidence of genetic divergence that might underlie the outcomes of plague infection in great gerbils. These regions encompass factors involved in transcription and translation, and the regulation of these processes. Notably, several genes are directly involved in immune functions, indirectly associated with immune function such as the regulation of apoptosis, as well as cellular metabolism. Hence, what determines the outcome of a plague infection in great gerbils might be a complex combination of differences in regulation of certain key immune and (intracellular) metabolic pathways affecting the ability to regulate and mobilize resources to fight the infection. It is also interesting to note that five of the challenged animals never appeared to be infected. The reasons for this are not clear and warrants further investigations.

### A genetic basis for differences in plague resistance in gerbils

Examining a disease trait in a single population is complicated, particularly if the disease resistance has a polygenic basis, then the causative variants are difficult to detect as they likely explain only a small proportion of the genetic variation (54). Despite an overall low genetic diversity of the gerbil population investigated, we identify several smaller genomic regions displaying genetic differentiation between survivors and moribund. We believe that the low level of overall genetic differentiation in fact, strengthens our ability to detect differences between individuals that can be connected to plague survival. For instance, in several pairs of individuals that appear to be full siblings, one survived the infection while the other was deemed moribund. Our findings suggest that specific alleles (or combinations of alleles) confer resistance. In particular, the genomic regions on scaffolds 22, 43, 59, and 102 identified in the *F*_ST_ analysis, are responsible for near complete and complete separation between the two groups with 46, 64, 72, and 52% of the variance in the structure explained, respectively. It should be noted, however, that the different genomic scans including *F*_ST_ and xpEHH gave a large number of potential candidate genes (Tables S9 and S10), whilst the list of overlapping genes between at least two of the analyses were relatively few (Table S11), in line with other studies using these methods (55, 56). One of these genes is *ZFAT*, which is located downstream one of the largest identified peaks, on scaffold00102, and encodes a DNA binding protein thought to be involved in transcriptional regulation. In mice, the *ZFAT* gene is strongly expressed in T cells and B cells of immune-related tissues such as the thymus, spleen, and lymph nodes (57), it plays a role in thymic T-cell development and peripheral T-cell homeostasis (58). In pigs, genetic variants of ZFAT has been linked to susceptibility to enterotoxigenic *Escherichia coli* infection (59). Another is the *GBP6* gene identified on scaffold00056 in association with xpEHH and *F*_ST_ peaks encoding a member of the family of guanylate binding proteins (GBPs), which are highly induced by interferon gamma and other inflammatory cytokines and are documented to be involved in innate immune functions mainly towards intracellular pathogens (60).

*Yersinia pestis* is known to manipulate the inflammatory response (61–64). One of the strategies used by the bacterium, is to induce immune cell death in neutrophils, macrophages, and dendritic cells by apoptosis as opposed to the highly inflammatory pyroptosis, effectively hampering the innate immune systems’ ability to properly respond to and alert the adaptive immune system about the ongoing infection (61, 65). In our study, we find evidence of positive selection on genes responsible for the regulation of apoptosis in surviving gerbils. It is possible that the immune cells of surviving individuals are more capable of resisting bacterial induced apoptosis due to genetic differences in these key apoptosis regulating genes. Multiple other candidate genes, including genes more directly involved in regulation of innate and adaptive immune responses, were identified in the *F*_ST_ analysis and the complementary xpEHH genome scan and are described and discussed in Note S2.

### Signatures of positive selection

Our analysis of selection in the population as a whole (i.e. the iHS analysis) identified several immune related genes which could indicate an increased general resistance to pathogens in this species. A proper proinflammatory environment is required during an infection for an effective activation and execution of adaptive cellular responses while avoiding hyperactivation that can lead to excessive collateral damage. One of the identified candidate genes, IL17A, belongs to a family of cytokines that are strong inducers of inflammation, signaling through a pathway that depends on the adaptor molecule ACT1 ultimately activating proinflammatory mediators such as the transcription factor nuclear factor-κB (NF-κB) (66). IL17A is expressed on several subsets of lymphocytes such as CD8 + T cells, natural killer (NK) cells, and NKT cells. Its main function has been shown to be neutrophil recruitment, and IL17A is considered an important driver of inflammation and immunity to extracellular pathogens due to the highly proinflammatory program of gene expression it induces (66). Notably, several IL17 receptors have previously been highlighted as potential players in plague resistance in other rodent species (22, 26). Furthermore, the candidate gene NLRP1B is also a contributor to innate immunity and inflammation as a sensor component of the NLR1 inflammasome (67). Inflammasomes are multimolecular complexes in the cell cytosol consisting of members of the nucleotide-binding domain-leucine-rich repeat (NLR) family proteins and inactive pro-caspase-1 (CASP1) (68). Once activated, CASP1 triggers downstream inflammatory responses resulting in pyroptotic cell death. Inflammasomes are known to be activated by directly detecting pathogen ligands or their activities such as bacterial effector proteins or toxins (52). In mice, NLRP1B was found to be activated in response to *Bacillus anthracis* lethal toxin and certain Nlrp1b alleles conferred resistance in mice to anthrax spore challenge through successful activation of the inflammasome and release of cytokines orchestrating a potent neutrophil response (67, 69). Investigations into great gerbil NLRP1B alleles might be an interesting avenue for further research into plague resistance in this species.

Some of the identified signatures of selection may be connected to the previously reported *MHCII DRB* gene duplication in gerbils (50), like the signal associated with the *PSD4* gene. Upregulation of *PSD4* is in this context of special interest as it controls the movement of *MHCII*-containing vesicles along the actin cytoskeleton in dendritic cells through the activation of the GTPase *ARL14* (ADP ribosylation factor like GTPase 14) (70). This might indicate that the gerbils have modified parts of the MHCII signaling system/pathway and how (effectively) bacterial antigens are presented to the adaptive immune system. Additional candidate genes are discussed in Note S3.

### Plague resistance in great gerbil impacted by sex-bias?

Our results could indicate that the great gerbil display a sex-bias in its plague resistance, as we see that females are more prone to die from plague, while males are better survivors. Differences in immune functions between males and females are well known in mammals and other animals, where it has been theoretically and empirically proven that females have a more sensitive whereas males a more specific immune system (71, 72), meaning that females are better at detecting a broad specter of pathogens while males mount a more efficient immune response. Recent studies in mice suggests that immune responses to microbial infections are influenced not only by mouse genotype but also sex as females had lower survival rates than males with the same genotype (73). Further scrutiny into specific alleles of candidate genes identified in this study should therefore also factor in sex.

### Gene expression differences in plague resistance

The upregulated pathways in moribund gerbils during plague infection are those commonly involved in fighting gram-negative bacteria, such as *humoral immune response*, *blood coagulation*, *response to lipopolysaccharide*, *acute inflammatory response*, and *myeloid leukocyte mediated immunity* (Table S13). In effect, some of the significantly enriched pathways and genes might promote plague infection by reducing the proinflammatory response and the differentiation and proliferation of host immune cells (74–76). However, determining which upregulated and downregulated genes and pathways that are driven by and are beneficial to the host and which are regulated due to manipulation by bacterial virulence genes is hard to disentangle. It should also be noted, that the differential expression analyses conducted in the plague-challenged gerbils was a comparison between moribund individuals sampled in the midst of their infection state vs. survivors sampled at the end of the experiment, when done fighting the bacterium. This sampling design did not allow us to identify how the survivors responded to the infection, since their potentially increased gene expression at the height of infection would have passed at the time of sampling. This set-up is a clear drawback for looking at differences in expression among gerbils dying from, or surviving a plague infection. However, design constraints limited the number of gerbils that could be included in the experiment. A stronger design would have been to sacrifice *n* survivors for every moribund individual, but would also require some form of biomarker for survival and/or death to make sure sampled individuals labeled as survivors are indeed going to survive the infection. This was and is currently not possible but would be important to pursue in future experiments.

### Concluding remarks

The outcome of an infection is determined by the combined effects of collateral damage caused by the host immune response and the direct (tissue) damage caused by the pathogen. Survival in this context might depend on the balance and timing of host responses. Here, we reveal evidence for selection on genes related to the innate and adaptive immune systems, as well as in basic cellular functions such as regulation of transcription, translation, and cellular metabolism in the great gerbil. The involvement of the innate immune system mirrors that of Busch et al., who found the innate immune system to be involved in plague resistance in prairie dogs (77, 78). Furthermore, we see evidence for selection on genes related to regulation of apoptosis which may be associated with an increased ability to survive a plague infection. Our findings suggest plague resistance is polygenic and that genetic differences among moribund gerbils and survivors are likely responsible for the outcome of the infection. Future work should aim to establish how these genetic differences confer functional differences in the regulation of cellular and immune functions during plague infection.

## Methods

### Study design, animals, and bacterial strain

This study design is based on the presence of a heterogenic response observed within gerbil populations (15, 16), i.e. that some individuals are plague-resistant, while others are susceptible and die after plague exposure. How this difference in response, however, is linked to genomic divergence due to past and present selection for plague resistance in the great gerbil is yet to be determined.

Subadult gerbils were captured within a 10 × 10 km area of the natural plague focus in the eastern parts of the Junggar Basin located in the Gurbatntünggut desert, Xinjiang, China. The average distance between the great gerbil burrows in this area is 80 m and the probability of capturing live gerbils is about 1 to 2 per burrow. Anticipating exclusion of individuals based on screening, no less than 180 gerbils were captured. The gerbils were housed in the field laboratory for at least 2 weeks prior to screening for *Y. pestis* F1 antigens and anti-F1 antibodies using up-converting phosphor technology-based lateral flow strips (79) and indirect hemagglutination assays (IHA), respectively (15, 80). Ultimately, about two-thirds of the 180 gerbils were excluded based on lower body weight (the younger ones) or positive F1 antibody status. Gerbils negative for both assays were used for subsequent animal challenges with the fully virulent *Y. pestis* strain 2505. The strain was isolated from a live great gerbil in Minai County of the Junggar Basin, Xinjiang, China in 2005 and has an LD50 of <10 in BALB/c mice (40).

### *Yersinia pestis* challenge

For screening and identification of individuals with either of the two phenotypes, we conducted a 22-day plague exposure experiment, where 45 gerbils (18 males and 27 females) were randomly divided into nine housing groups (*n *= 5 per group). The gerbils in each housing group were marked with nontoxic dyes from the root of the hair; by the head, and the left front, right front, left back, and right back of the extremities with gerbil no. 1 to 5 marked individually. After anesthesia by ether inhalation, all 45 gerbils were subcutaneously injected in the groin with 1 ml of *Y. pestis* 2505 culture suspended in physiological saline (5.6 × 10^9^ CFU/ml). A subcutaneous injection was chosen as it allows for the required volume containing the large dose of plague to be injected. The bacterial suspension was prepared in a single batch and quantified by the turbidimetry McFarland method, and the cfu/ml of the suspension was also performed by the conventional plating method. The injection process was completed within 1 h and during the injection, the suspension was mixed continuously and gently with a mixer. Another nine gerbils (three males and six females) were injected with 1 ml of physiological saline to serve as a handling control (making sure the animals did not succumb to stress from the procedure). All the gerbils were raised group-wise in filter-top cages (M5 type; 475 × 350 × 200 mm) arranged in a single layer in the order of housing group in an air-conditioned room set at 22°C and humidity at 30% with chow and water ad libitum. The light cycle was 14 h during the day (illuminance 100 to 200 lx), and 10 h at night. All challenged individuals were observed and scored twice per day by two independent laboratory personnel with long time experience evaluating clinical signs of plague (including increased anal temperature, polydipsia, closed eyes, ruffled fur, hunched posture, and lethargy) (15). For instance, gerbils in a diseased state is curled all over the body (and not able to prostrate or lay on its back), with no responses to stimuli, with an accelerated course of the disease between morning and afternoon. The above-mentioned clinical signs are keys to determine whether the diseased gerbil should be deemed (i) moribund or evaluated to (ii) survive to the next day. If there is a response, and there is little difference of the status between the morning and afternoon, it can continue to live till next day. Therefore, the animal was not deemed moribund at the onset of symptoms, instead, the decision to euthanize an animal was based on these observations and by the above-mentioned judgement. The animals were classified into two different categories: (i) moribund (those that showed signs of severe plague symptoms and concurrently lack of vitality) and (ii) surviving, which was further subdivided into those that recovered (displaying signs of plague symptoms but with continued vitality and no accelerated disease progression), and healthy (those that showed no signs of plague infection) throughout the experimental period. The moribund animals were euthanized as soon as signs of obvious disease appeared. In total, 21 challenged, moribund gerbils were euthanized at different days p.i. In addition, one gerbil was found dead at day 13 p.i., and was dissected aseptically to collect the liver. Surviving animals and the control individuals were euthanized at day 22 post infection (p.i.). Four of the five individuals showing no signs of symptoms during the trial (i.e. the healthy individuals) were euthanized on day 3 and 4 p.i. while the fifth was euthanized on day 22 p.i. Immediately after euthanasia the animals were dissected aseptically to collect the liver. See Table S1 for details and metadata of all gerbils.

The liver tissue samples were split in two. Half of the collected liver tissue was snap frozen in liquid nitrogen and then transferred to clean tubes and kept at -20°C until DNA extraction. The remaining half of the animal’s livers were cut into smaller pieces and submerged in 5 ml RNA*later* (Ambion), incubated at 4°C overnight and then frozen at -20°C until RNA extraction. Ten moribund and 10 surviving individuals (*n *= 20, 9 males and 11 females) were whole genome sequenced at an average 12× coverage per individual (see details in next section).

Animal challenge experiments and the use of great gerbil tissue in this study were performed abiding by the biosafety and ethical regulations issued by the Ministry of Health, China, and approved by the Committee for Animal Welfares of Xinjiang CDC. Sampling was performed prior to China’s signature of the Nagoya Protocol (date of accession: 2016 September 6). The sampled species have a “least concern” status in the IUCN Red List of Threatened Species.

### DNA extraction and sequencing

DNA from gerbil livers was extracted using Qiagen Blood and Tissue DNeasy kit (Qiagen Inc., USA) following the manufacture’s protocol. DNA extractions were freeze dried prior to shipping to the University of Oslo and upon arrival resuspended in 200 μl E.Z.N.A. Elution buffer (Omega Biotek) and placed in a heating block at 37°C for 4 h. The extractions were then analyzed with Qubit (Thermo Fisher Scientific), NanoDrop (Thermo Fisher Scientific), and Bioanalyzer (2100, Agilent Technologies) to assess the quality and quantity of DNA. DNA samples from ten survivors and ten moribund individuals (*n *= 20) from the experiment, were selected for DNA sequencing based on high-quality DNA (Table 1). Prior to library prep, an additional 100 µl E.Z.N.A. Elution buffer was added to the samples due to high DNA yields. Library prep was performed using the Illumina TruSeq DNA PCR Free protocol and the samples were sequenced using Illumina HighSeq 2500 with a 350 bp insert size at the Norwegian Sequencing Centre (NSC), University of Oslo, Norway.

### Repeat masking, read alignment, and variant calling

Prior to population genomic analyses, all types of repeats were masked from the gerbil reference genome (50) using RepeatMasker v4.0.6 (81) with default settings and “rhombomys opimus” species filter to avoid calling variants in these areas (82). Thirty-four percent (34%) of the 2.47 Gb gerbil reference genome was masked by RepeatMasker prior to mapping and included all types of repeats (Table S17). Raw sequence reads were trimmed for Illumina adaptors and low-quality reads using Trimmomatic v0.36 (83). Reads with an average quality of less than 20 across 5 bp step windows were removed as well as reads below 40 bp in length. Trimmed and filtered reads (paired only) were mapped to the repeat masked reference genome using bwa-mem v0.7.8 (84) with default parameters except adding an -M parameter to enable Picard (http://picard.sourceforge.net) and also specifying read group. As each individual was sequenced on multiple lanes, each sequence lane was mapped separately and then merged to produce a final bam file for each individual. Bams were then sorted, filtered for duplicates, and indexed using Picard v1.72. Bams were realigned around insertion–deletion polymorphisms and variant calls were made for all sites using the GATK HaplotypeCaller of GATK’s Genome Analysis Toolkit v3.7 (85, 86). The raw vcf was filtered to extract SNPs only and hard filtered according to GATK’s recommendations, including filtering on QualByDepth (QD, with thresholds adjusted to fit the data, see Fig. S11). In the process, the SNPs in the vcf were annotated with filter thresholds. For downstream analyses, additional filters were applied creating a high-quality dataset, which included only SNPs occurring in all individuals and a minor allele threshold of 0.05 (MAF < 5%) and DP > 4 (removing DP < 5) (hereafter “filtered variants”).

### Population structure estimation

LD decay was calculated across each of the 20 largest scaffolds (10% of the assembled genome) using the SNP set of filtered variants. Pairwise *r^2^* values were calculated between all SNPs within a 500 kb window in Plink v1.90b3b (87). Decay plots (Fig. S12) were created by binning the distance between SNPs in increments of 1 kb and averaging the *r^2^* values within each bin (88). To investigate population structure, a second dataset (hereafter “LD-pruned”) was created by further performing linkage pruning on the filtered variants set using Plink2 v2.00a2LM (89) filtering for all loci within 100 kb windows with an *r^2^* exceeding 0.1. Genome window size was then determined based on the rate of LD decay from the 20 largest scaffolds (Fig. S12). PCA was then performed on the LD pruned variants using Plink2 v2.00a2LM. As the gerbils were captured within a relatively small area, we tested the individuals for familial relationship by running IBD calculations on the LD pruned dataset using Plink v1.90b3b. This allowed us to see if relatedness had an impact on the clustering pattern observed in the PCA and to evaluate if any individuals should be excluded from downstream analysis.

### Phasing and demographic analyses

A separate filtering approach was used on the raw, unfiltered dataset prior to phasing and demographic analyses. First, scaffolds under 1 Mb were excluded from the dataset and the remaining SNPs and genotypes were filtered on low quality using vcflib (DP—summed across individuals—between 40 and 600, FS < 40, QUAL > 19.99). To test for robustness of the demographic reconstruction to individual genotype, minimum coverage and to missing data per locus, we repeated the analyses filtering for individual genotype DP > 2 and DP > 4 as well as filtering out loci with more than 20% missing individuals (Fig. S3).

Phasing was performed on these two variant datasets (hereafter “phased variants”) using beagle v4.1 with default parameters and no imputation. We wanted to investigate the recent demographic history of the gerbil population to see if any population decline could be linked to the emergence of plague. Since plague is a young disease, this required a sensitive method with good ability to model the recent past of the gerbil population (90). Demographic analyses using MSMC2 (90), were performed on the phased variants of the 21 longest scaffolds only, all over 1 Mb in size. Five randomly generated MSMC2 input files were created, each containing 3 of the 20 individuals, and used in replicated runs. The results were plotted in R with a 1-y generation time and a mutation rate of 5 × 10^−9^ (sub/site/gen) (91, 92).

### Genome scans for signatures of selection

To identify genomic regions and potential candidate genes involved in plague resistance, we looked for signs of selective sweeps at two levels; first, in the populations as a whole and second, looking for differences between survivors and moribund individuals by treating the two groups as two separate phenotypes in pairwise-population analyses.

### iHS and xpEHH

We calculated iHS and xpEHH from the phased variants (DP > 4 only) to look for signs of recent positive selection in the gerbil population as a whole (93) using the R package *rehh* 2.0 (94). Whereas iHS is designed to identify signatures of selective sweeps within populations, xpEHH is a comparative statistic that identifies divergent haplotype structure between pairwise populations. In this case, we used xpEHH to investigate differences in haplotype homozygosity between gerbils classified as moribund or survivors after the challenge experiment. Scripts for converting the phased vcf into *rehh* input files were modified from (95). Due to the discovered relatedness among the individuals in the experiment, we conducted sensitivity analysis on a single scaffold, scaffold00080 as it has the most significant xpEHH peak, by dropping one individual at a time and recalculating xpEHH to see if the results changed significantly (Fig. S5).

### *F*_ST_,simulations, and nucleotide diversity analyses

Genome-wide *F*_ST_ was calculated with the R package PopGenome v2.6.1 (96) on the variant filtered set, which had been divided into scaffolds using SnpSift v4.0 (97). Sliding-window *F*_ST_ was calculated on a 50 SNP basis (width = 50, jump = 25) using the sliding.windows.transform flag. The same parameters per SNP were calculated on a concatenated dataset. All 50-SNP windows with *F*_ST_ values ≥ 95th percentile (i.e. a value of 0.2) were extracted and the corresponding scaffolds were identified (Table S5). Per SNP, *F*_ST_ values for the identified scaffolds were plotted to visualize the *F*_ST_ distribution within these scaffolds (Fig. 2).

PCA, DAPC plots, and compoplots were constructed for all scaffolds with *F*_ST_ 50-SNP windows > 0.2 to visualize clustering between moribund and survivors, using the R packages VcfR 1.8.0 (98)) and Adegenet 2.1.1 (99). Due to the discovered relatedness and since the *F*_ST_ values are based on calculations from only 20 individuals (10 in each “population”), the robustness of the results was evaluated by calculating *F*_ST_ values after a leave-one-out procedure on one of the scaffolds (scaffold00102) (Fig. S7). This scaffold was chosen as it had a large peak containing the highest calculated *F*_ST_ value (*F*_ST_ = 0.57). Nucleotide diversity (π) was calculated in sliding windows of 100 kb in 25 kb steps using vcftools v0.1.14 (100), for the moribund and survivors separately.

To justify the 95th percentile *F*_ST_ threshold and to generate a null *F*_ST_ distribution, neutral simulations were performed using SLiM3 (101) under a demographic history informed by the results of the MSMC2 analyses. A population of 10,000 individuals, each with a 1 Mb chromosome was simulated for 10,000 generations. Simulations also included exponential population growth to 25,000 individuals after 8,000 generations. At the end of each simulation, 20 individuals were randomly sampled from the population pool, randomly assigned to two groups and *F*_ST_ was calculated. Simulations were repeated 100 times to give an indication of the null distribution of mean *F*_ST_ and the upper 95th percentile.

We additionally performed simulations allowing a single adaptive mutation to arise at the center of the 1 Mb chromosome after 8,000 generations. This mutation was assigned a selection coefficient of 0.5 and was allowed to sweep to fixation until it reached a frequency of 0.6 when it became neutral (i.e. selection coefficient set to zero). A partial selective sweep like this is indicative of the kind of selection we expect to produce standing adaptive variation in the gerbil system. At the end of each simulation, we randomly sampled 10 individuals with the allele and 10 without it, calculating *F*_ST_ among them.

### Gene enrichment analysis of candidate genes

We extracted candidate genes surrounding the outlier peaks from the iHS and xpEHH analyses at a 250 kb distance using scripts modified from (95). For the iHS and xpEHH outliers, we set the threshold at a log10 *P-*value of 6 (this is equivalent to a *P* = 1 × 10^−6^) prior to candidate gene extraction. We also extracted candidate genes within 250 kb of all *F*_ST_ > 0.2 windows found on the 30 scaffolds with high *F*_ST_ regions. The gene lists were filtered to contain only a unique set of gene IDs and names were matched to human orthologs before running ontology analyses using clueGO in Cytoscape v3.7.0 (102). All analyses were run with medium network specificity, in a right-sided hypergeometric test with Benjamini-Hochberg FDR correction, only reporting pathways with a *P* < 0.05. For enriched pathways highlighted in the results, all *P*-values are after FDR correction. The identity and function of candidate genes were also examined manually through web-based searches.

### RNA extraction, sequencing, and differential expression analysis

RNA was extracted from the liver samples preserved in RNAlater using a standard chloroform procedure (103) and the samples were stored at -80°C. Library prep and sequencing were conducted at the Beijing Genomics Institute (BGI, https://www.bgi.com/us/sequencing-services/dna-sequencing/) using Illumina TruSeq RNA Sample Prep Kit and PE sequencing on the HiSeq4000 instrument (150 bp read length). In total, eight individuals from each group (eight moribund and eight surviving, *n *= 16, seven males and nine females) were RNA sequenced, comprising of 15 of the DNA sequenced individuals and one additional individual ( Table 1 and Table S1).

The raw sequence reads were trimmed using trimmomatic v0.36 and mapped to the gerbil reference genome using STAR v2.5.2a (104) with default parameters. The average number of reads used for the mapping was 100 million per individual. A less stringently filtered annotation file for the reference genome than presented in Nilsson et al. (50) was used in the mapping process. A raw count matrix was created using htseq v0.7.2 with strandedness option set to “no” and otherwise default parameters to extract raw counts from the mapped files. The annotation file was then used to extract each feature count. Prior to normalization and differential expression (DE), the count matrix was filtered by requiring expression in minimum two libraries and excluding genes with read counts < 1 across all samples. Normalization and dispersion were calculated using rmFactors, estimateDisp, estimateCommonDisp and estimateTaqwiseDisp using default parameters. One individual was excluded from further analysis due to high levels of individual variation that would reduce the power of downstream differential expression (Note S4 and Fig. S13). Differences in expression between moribund gerbils and survivors were analyzed using the edgeR package in R (105). Differential expression was calculated using exacttest (function exactTest) between moribund and survivor. Resulting differential expressed genes were filtered using *P* < 0.05. The generated list of genes with adjusted *P*-values (FDR < 0.05) were further analyzed for significantly enriched pathways in ClueGO in Cytoskape v3.7.0. For the enrichment analyses, the complete list of genes was analyzed as well as lists separated by genes that were upregulated and downregulated in the moribund individuals. The three enrichment analyses were run with medium network specificity in a right-sided hypergeometric test with Benjamini–Hochberg FDR correction and *P* < 0.05. Finally, the identity and function of DE genes was also examined manually through web-based searches.

## Supplementary Material

pgac211_Supplemental_Files

## Data Availability

The version of the reference genome used in this paper as well as the two differentially filtered annotation files are available at Figshare: https://figshare.com/s/9035ed40f970d0545d06 The genome assembly used here is a previous version of the assembly reported in Nilsson et al. (50) prior to NCBI removal of a small duplicated gene and masking of minor contamination. Raw read data analyzed in the current study have been deposited in the European Nucleotide Archive (ENA, www.ebi.ac.uk/ena) under study accession number PRJEB45416, individual accession numbers ERS7641382-ERS7641401 (whole genome sequencing) and ERS13531117–ERS13531132 (RNA sequencing).
