# Supplementary material for: Polygenic plague resistance in the great gerbil uncovered by population sequencing
Source: PNAS Nexus. 2022 Oct 5;1(5):pgac211. doi: 10.1093/pnasnexus/pgac211 (PMC9802093; doi:10.1093/pnasnexus/pgac211)
Supplement: pgac211_Supplemental_Files [file pgac211_supplemental_files.zip › PNASNEXUS-PNASNEXUS-2022-00082-s01.pdf]

Supplementary Materials

Polygenic plague resistance in the great gerbil uncovered by population sequencing

Pernille Nilsson, Mark Ravinet, Yujun Cui, Paul R. Berg, Yujiang Zhang, Rong Guo, Tao Luo, Yajun Song, Emiliano Trucchi, Siv N. K. Hoff, Ruichen Lv, Boris V. Schmid, W. Ryan Easterday, Kjetill S. Jakobsen, Nils Chr. Stenseth, Ruifu Yang and Sissel Jentoft

Supplementary Note S1	p. 2
Supplementary Note S2	p. 2
Supplementary Note S3	p. 5
Supplementary Note S4	p. 5
Supplementary Figures S1-S13	p. 6 – 31
Supplementary Tables S1-S17	p. 32 – 45
References	p. 46 – 47

Supplementary Notes

Note S1: ***Signatures of past population decline***

Our demographic analysis of the gerbils shows a general declining trend in effective population size over the last 200 K years that appears to plateau around 20-50 K years ago (Figure S3). This coincides with expansion of deserts and sand fields in China occurring during the onset of the Last Glacial Maximum approximately 26 Kya characterized by a cold-dry climate. From around 10 Kya there is a strong decline with a minimum in population size by around 4-5 Kya. This is consistent with a reduction in available desert habitat during the Holocene Optimum around 5-9 Kya, when a transition to a warm-wet climate led to increased vegetation growth that nearly completely covered the sand fields [1]. Effective population size reaches its minimum around 5 Kya, which likely explains the extremely low degree of nucleotide diversity observed. When effective population size becomes low, genetic drift will also be stronger than selection further reducing the diversity [2]. The gerbils were captured in a relatively small area and in reality, are highly likely to be part of the same (meta)population. Hence, the low divergence we observe most likely also reflects the relatively small geographic range of the sampling area in combination with the social structure and patterns of dispersal for gerbils [3,4]. As the individuals in this experiment were all of same age (i.e. sub-adults) the potential relationship would be full and half-siblings. Notably, even though there are some degree of relatedness, we do detect differences in plague resistance. We believe the low level of genetic differentiation strengthens our ability to detect differences between individuals that can be connected to plague survival. For instance, in several pairs of individuals that appear to be full siblings, one survived the infection while the other was deemed moribund. This might suggest that specific alleles (or combinations of alleles) could confer resistance.

Whether plague (or a similarly lethal disease) might have impacted the gerbil population during the last 6 Ky is difficult to determine due to the recent evolutionary history of the pathogen, but is a prediction that could be tested in the future with a more detailed demographic analysis.

Note S2: ***Candidate genes identified in the complementary genome scans locating differences between moribund and surviving gerbils.***

Several significant xpEHH peaks are found within genes including *CXCL14* (C-X-C Motif Chemokine Ligand 14), *OR13A1* (olfactory receptor 13A1) and *BPGM* (bisphosphoglycerate mutase) on scaffold00061, scaffold00548 and scaffold00376 encoding a chemokine, an olfactory receptor and a trifunctional enzyme with mutase,

synthase and phosphatase activity, respectively (Table S8) [5-7]. Two of the top 10 most significant xpEHH peaks are found on scaffold00056 and scaffold00136 (Table S5). Within scaffold00056 the peak overlaps with the *ABCG3* (ATP-binding cassette sub-family G member 3) gene and *GBP6* (Guanylate-binding protein 6) is located downstream of the peak, closely associated with other outlier values (10 kb downstream of closest significant outlier), while *CYCS* (Cytochrome C, Somatic) is located just downstream (41 kb) of the peak on scaffold00136 (Figures 4A and S10D). In addition, among the candidate genes are *CD180* (previously known as *RP105*), encoding a cell surface molecule present on immune cells, and located just upstream (42.5 kb) of a highly significant xpEHH peak on scaffold00091 (Figure S10B).

We further identified 565 unique genes falling within 250 kb on either side of the elevated F_{ST} regions (F_{ST} > 0.2) on the 30 identified scaffolds with higher divergence. GO analysis revealed 11 enriched pathways among the gene set and included pathways involved in innate immunity, intracellular transport and translation; *Macrophage activation involved in immune response* (p = 0.031), *SRP-dependent cotranslational protein targeting to membrane* ($p = 1.68 \times 10^{-11}$) and *ribosome biogenesis* ($p = 4.72 \times 10^{-5}$) (Table S10). We identified 32 genes that had one or more significantly elevated SNP located within their annotated boundaries (Table S8).

A gene of particular interest from this subset is *MLF1* (myeloid leukemia factor 1) on scaffold00022 due to its involvement in lineage commitment of primary hemopoietic progenitor cells (Figure S10C). No known genes fall within the cluster of large F_{ST} values containing the highest peak on scaffold00102, but two genes were located nearby (Figure 4B). These were *FTSJ2* (also known as *MRM2* - mitochondrial rRNA methyltransferase 2) located upstream (185 kb) of the peak and *ZFAT* (zinc finger and AT-hook domain containing) located downstream of the peak (177 kb downstream of closest F_{ST} > 0.2). The three candidate genes (*MLF1, FTSJ2, ZFAT*) are all involved in regulation of transcription or believed to modify components involved in translation. Two of them (*ZFAT* and *MLF1*) are implicated in regulating cell lineage commitment of various cell populations of the immune system. *ZFAT* is located downstream one of the largest identified peaks, on scaffold00102, and encodes a DNA binding protein thought to be involved in transcriptional regulation. In mice, the *ZFAT* gene is strongly expressed in T cells and B cells of immune-related tissues such as the thymus, spleen and lymph nodes [8], it plays a role in thymic T-cell development and peripheral T-cell homeostasis [9] and has also been identified as an antiapoptotic molecule in a human leukemia cell line [10,11]. Several diseases have been linked to genetic variants of *ZFAT*, including autoimmune thyroid disease and severity of Hashimoto disease in humans and susceptibility to enterotoxigenic *Escherichia coli* infection in pigs [12-14]. *MLF1*, overlapping with an F_{ST} peak on scaffold00022, is involved in lineage commitment of primary hemopoietic progenitor cells restricting erythroid formation and enhancing myeloid formation [15]. In humans, *MLF1* is also known as an oncogene where overexpression in hematopoietic cells is associated with acute myeloid leukemia [16]. It is tempting to speculate that there could be differences in critical cell

populations of the immune system that tips the scale in the gerbil's favor for the surviving individuals.

Y. pestis is known to interfere with numerous proteins encoded by many genes and immune pathways manipulating the inflammatory response [17-20]. One of the strategies used by the bacterium, is to induce immune cell death in neutrophils, macrophages and dendritic cells by apoptosis as opposed to the highly inflammatory pyroptosis, as well as globally depleting natural killer cells, effectively hampering the innate immune systems' ability to properly respond to and alert the adaptive immune system about the ongoing infection [17,21]. In our study, we find evidence of positive selection on gene(s) responsible for the regulation of apoptosis, i.e. the *CYCS* gene in the surviving gerbils. The *CYCS* gene identified on scaffold00136 by the xpEHH analysis (Figure 4D) encodes the small heme-containing protein, cytochrome *c*, which is central in mitochondrial respiration by transferring electrons to the respiratory chain to maintain ATP function [22]. However, it is also involved in the apoptotic pathway when released into the cytosol where it can trigger the activation of the caspase cascade in intrinsic apoptotic pathway [22]. Its release occurs through a pore in the outer mitochondrial membrane generated by the oligomerization of the protein product of the *VDAC1* (Voltage dependent anion channel 1) gene, which is associated with an F_{ST} peak identified on scaffold00043, as well as signatures for selection in xpEHH and nucleotide diversity analyses (Figure S10A) [23]. It is possible that the immune cells of surviving individuals are more capable of resisting bacterial induced apoptosis due to genetic differences in these key apoptosis regulating genes.

We also discovered genetic differences associated with genes more directly involved in regulation of innate and adaptive immune responses between surviving and dying gerbils. The *GBP6* gene identified on scaffold00056 in association with an xpEHH peak encodes a member of the family of guanylate binding proteins (GBPs) which are highly induced by interferon gamma and other inflammatory cytokines and are documented to be involved in innate immune functions mainly towards intracellular pathogens [24]. Other GBP family members have been shown to control bacterial or protozoan infections in studies of *Listeria monocytogenes* and *Toxoplasma gondii,* conferring some resistance in murine models [25]. Furthermore, we uncovered genetic divergence between the two gerbil groups regarding the cell surface molecule CD180. The pattern recognition receptor CD180 dimerizes with MD1 forming a cell surface complex working in concert with TLR4 on antigen presenting cells like macrophages, B cells and dendritic cells, controlling the recognition and signaling of lipopolysaccharide (LPS) found in gram-negative bacteria [26,27]. In studies investigating LPS-induced systemic inflammation and sepsis, CD180 appeared to be involved in dampening inflammatory responses [28]. CD180 has also been shown to have a potential role in host resistance to infections caused by other pathogens like *Staphylococcus aureus* [29]. CD180 could potentially be an important regulator of immune responses during plague infection in a way that increase survival, as the signatures of selection associated with CD180 appeared to have occurred in the survivors (Figure S10B).

Note S3: ***Signatures of positive selection in the gerbil population***

Several candidate genes identified in our genome scans has functions that can be connected to cytoskeletal functions. The connection to actin cytoskeleton regulation is particularly interesting as *Y. pestis* is known to impair the cytoskeletal dynamics of phagocytes, blunting phagocytosis and paralyzing dendritic cell movement in the host [30,31]. The seventh strongest signal of selection in the iHS analysis was located upstream of *ARHGEF25*. As a guanine nucleotide exchange factor for Rho family of small GTPases, it is reportedly involved in multiple physiologic functions such as vascular smooth muscle contractility and cell movement [32-35].

Note S4. ***Degree of variation in RNA libraries.***

The DESeq R package was used to estimate the degree of variation in each of the RNA libraries to investigate potential issues to take into account or samples to exclude in downstream RNA analyses. The method was applied to investigate and discover potential library differences that might bias the differential expression (DE) analyses. Boxplots of the dispersion of each library with the log10 of normalized gene counts on the y axis revealed a particularly large dispersion for individual S5-2 and prompted its exclusion from the final DE analyses performed with the R package edgeR (Figure S13).

Supplementary Figures

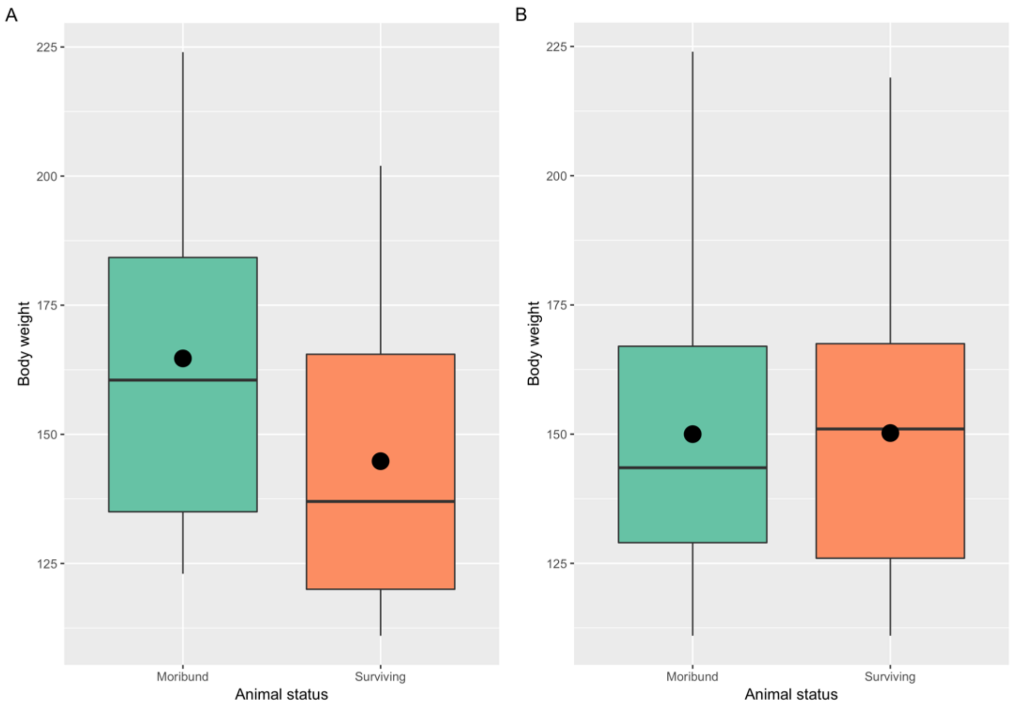

Figure S1. ***Boxplot of the outcome of the challenge experiment and body weight.*** Visualization of the outcome of the results for A) the whole genome sequenced individuals only (Table 1) and B) all challenged gerbils in the experimental setup (Table S1, the single dead individual excluded). The black dot represents the average for each group.

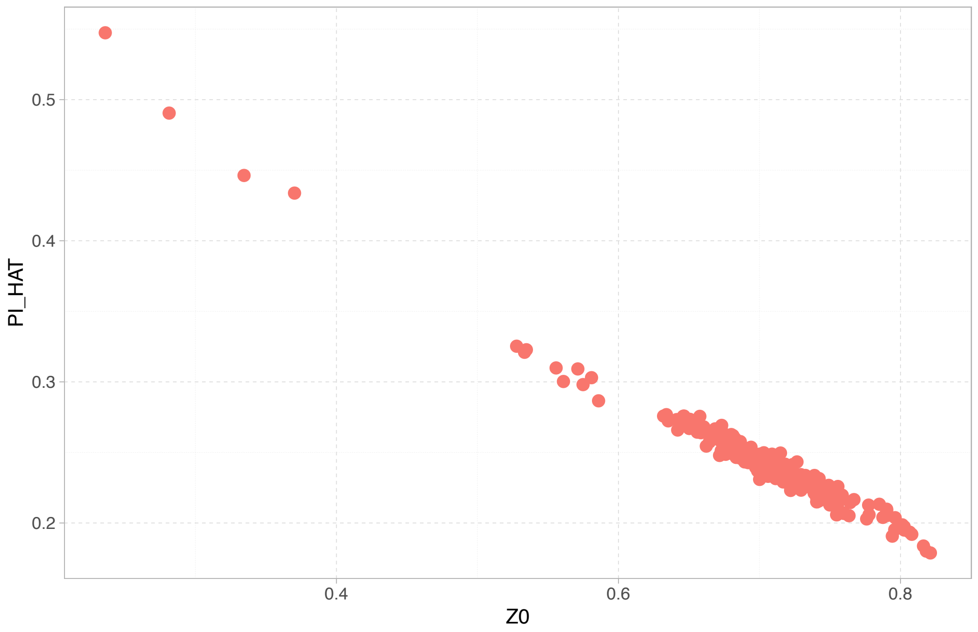

Figure S2. ***Estimation of relatedness between samples.***
The plot visualizes the results of pairwise comparisons of individuals using the IBD calculations in *plink*. Four pairs of individuals are revealed to be closely related with PI_HAT scores close to or above 0.5 which indicate they might be full siblings. Z0 is the probability of the pair sharing 0 alleles at any given site, and should be ~ 1 for unrelated individuals. Pairs with PI_HAT scores above the set threshold of 0.05 was reported, revealing any form of relatedness in the samples. See Table S3 for detailed values and further explanations.

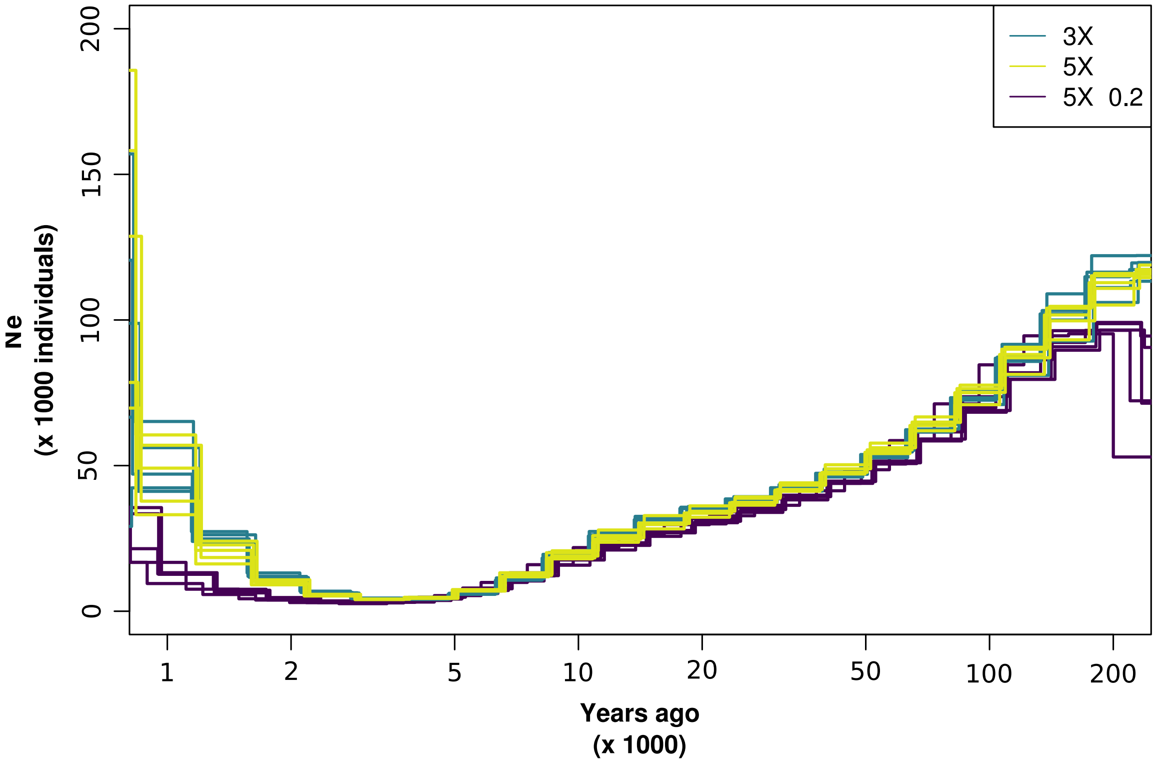

Figure S3. ***Demographic inference of great gerbil demography.***
MSMC2 approach was applied to estimate effective populations size in five random replicates of three individuals at a time. Different filtering strategies concerning individual genotype coverage (minimum 3X in light blue or minimum 5X in yellow) and maximum proportion of missing individuals per locus (max 20 % in purple or no limitation for the other two colors) were tested.

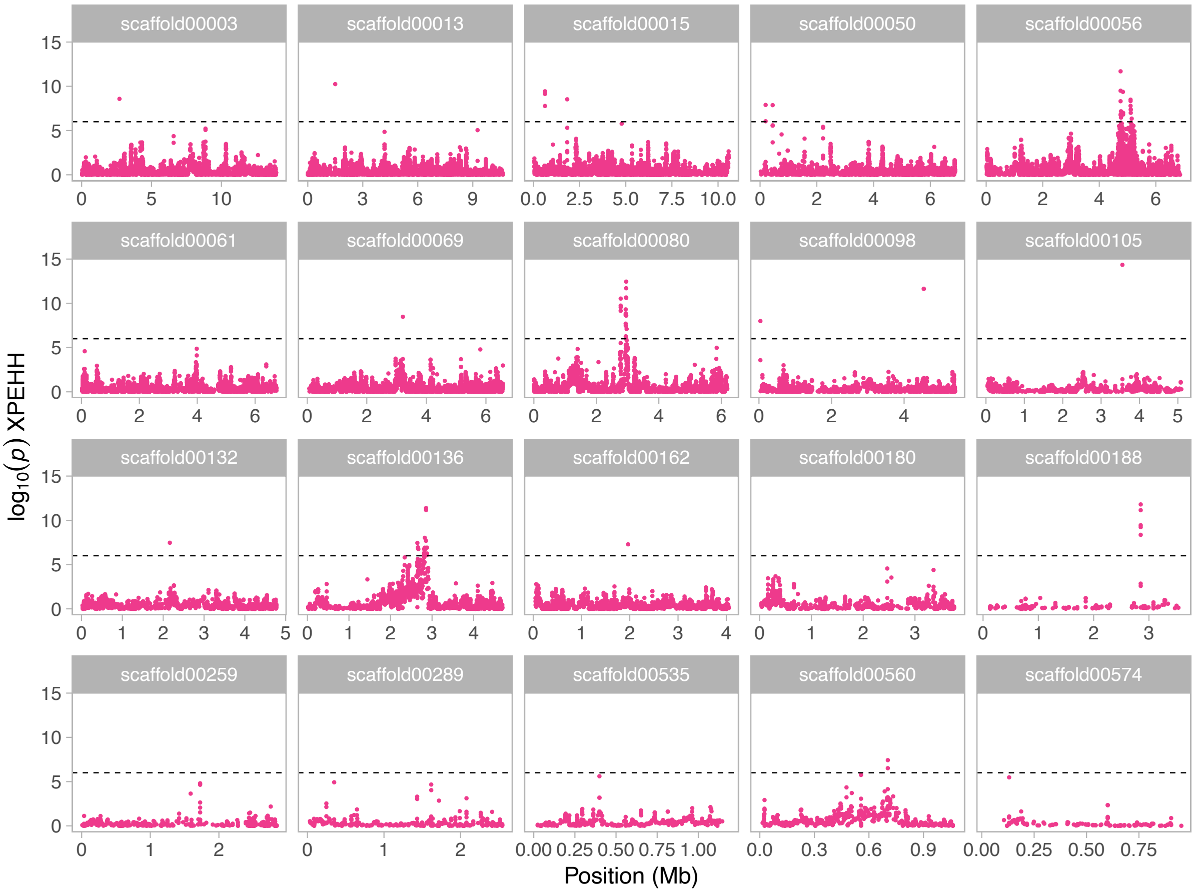

Figure S4. ***Signatures of recent selection for the 70 most significant clusters/peaks.*** The plots of xpEHH peak signals in the great gerbil population shows peaks of high haplotype homozygosity i.e. strong signals of selection, with particularly large peaks on scaffolds 56, 80 and 136. Horizontal dashed line represents threshold of significance ($\log_{10}$ 1×10^{-6}) for outlier SNPs. The data was subsampled to 0.2 using the sample_frac() command of the dplyr R package prior to plotting for visualization purposes only. As this command randomly selects a fraction of the values, it occasionally looks like there are no outlier peaks above the set threshold for some of the scaffolds.

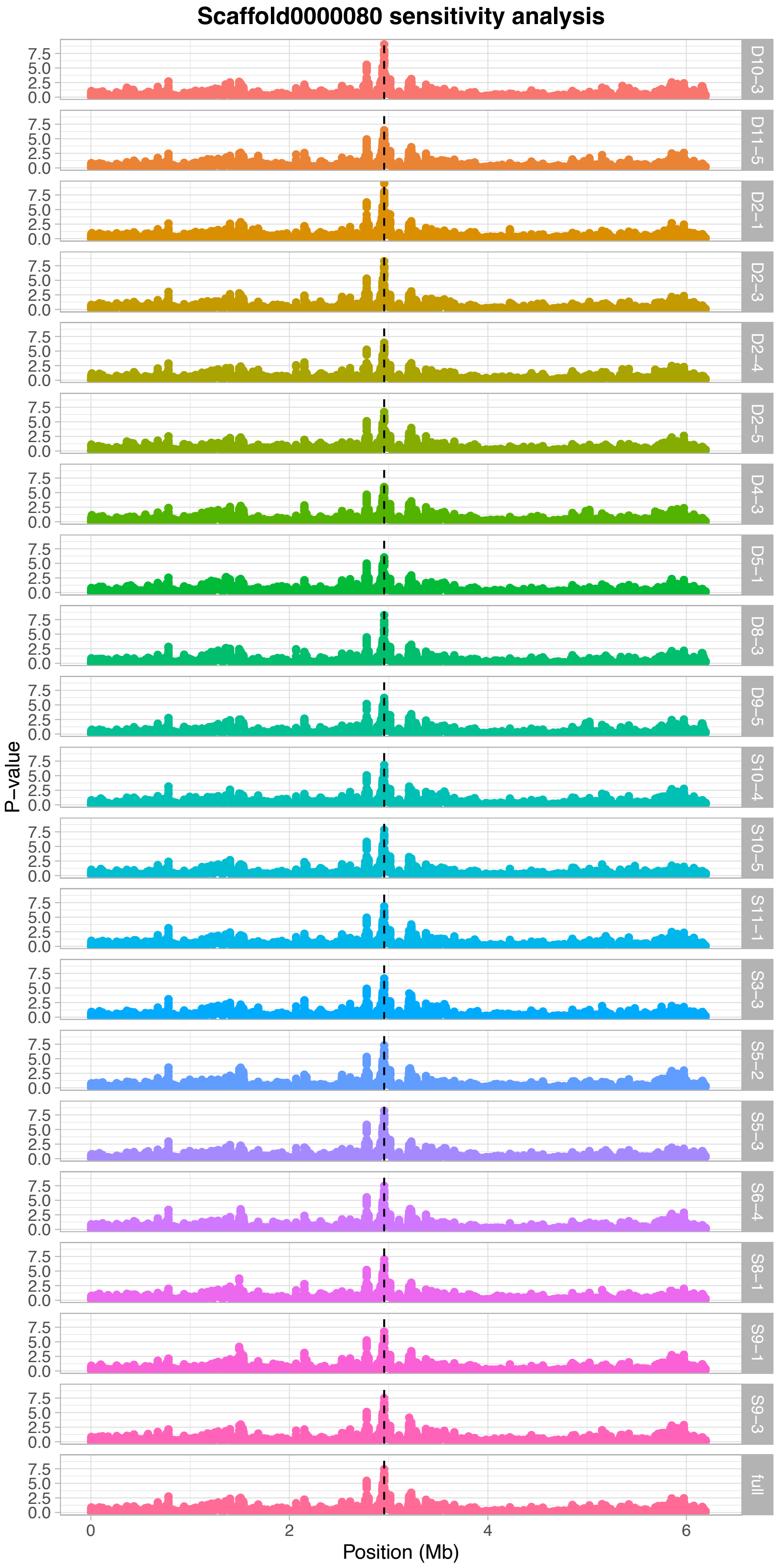

Scaffold0000080 sensitivity analysis
P−value
Position (Mb)
D10−3
D11−5
D2−1
D2−3
D2−4
D2−5
D4−3
D5−1
D8−3
D9−5
S10−4
S10−5
S11−1
S3−3
S5−2
S5−3
S6−4
S8−1
S9−1
S9−3
full

Figure S5. ***Sensitivity analysis of scaffold00080 xpEHH peak.***

The robustness of the xpEHH peak signal was evaluated by excluding one individual at a time and recalculating xpEHH to see if it altered the results. The dashed line is the position of the highest identified peak (*p* value 14.7 at position 2 954 856) and the name of the excluded individual is displayed in the facet. The full dataset is plotted at the bottom of the figure. The signal is quite robust with some fluctuations in the size of the peak, but is maintained in all scenarios.

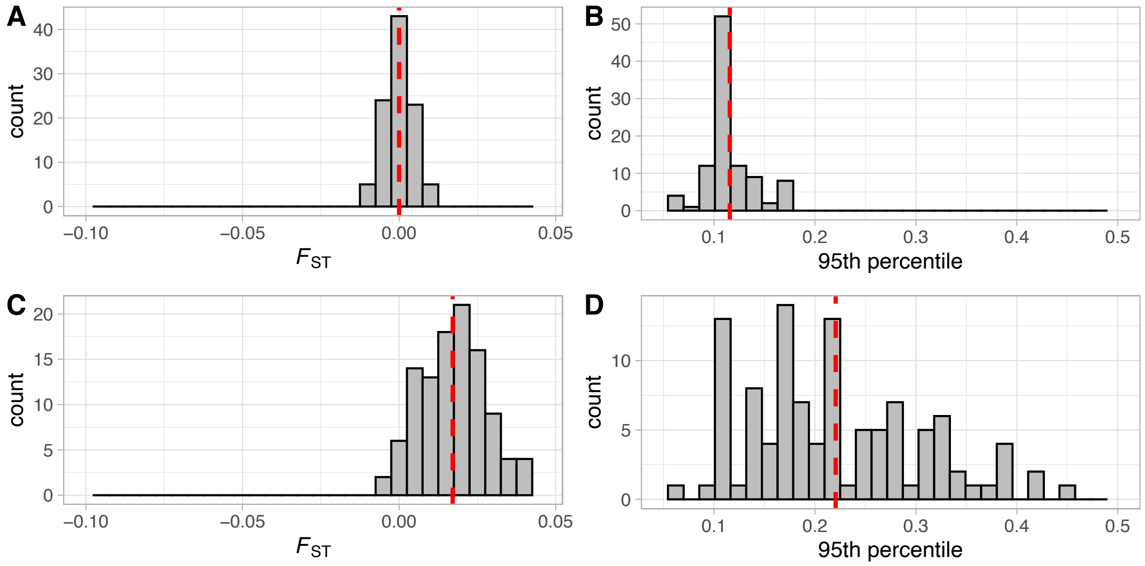

Figure S6. ***Forward-in-time simulations.***

A) Null distribution of mean genome-wide FST for 100 forward-in-time-simulations under neutrality; (B) Null distribution of the upper 95th percentile for 100 forward-in-time simulations under neutrality. (C) Distribution of mean genome-wide FST for 100 forward-in-time-simulations with selection; (D) Distribution of the upper 95th percentile for 100 forward-in-time simulations with selection. Dashed red line denotes mean value for each distribution. Neutral simulations mimicked the demographic scenario identified by our MSMC analyses; we simulated 10,000 individuals with a 1Mb chromosome, allowing only neutral mutations for 10,000 generations. After 8000 generations, we allowed an exponential increase of the population size to 25,000 individuals and then randomly sampled 20 individuals and sorted into 2 phenotypic treatments. Selective simulations (C & D), were the same as neutral with the inclusion of a single adaptive mutation that arises after 8000 generations and undergoes a partial selective sweep to a frequency of 0.6 before 10,000 generations.

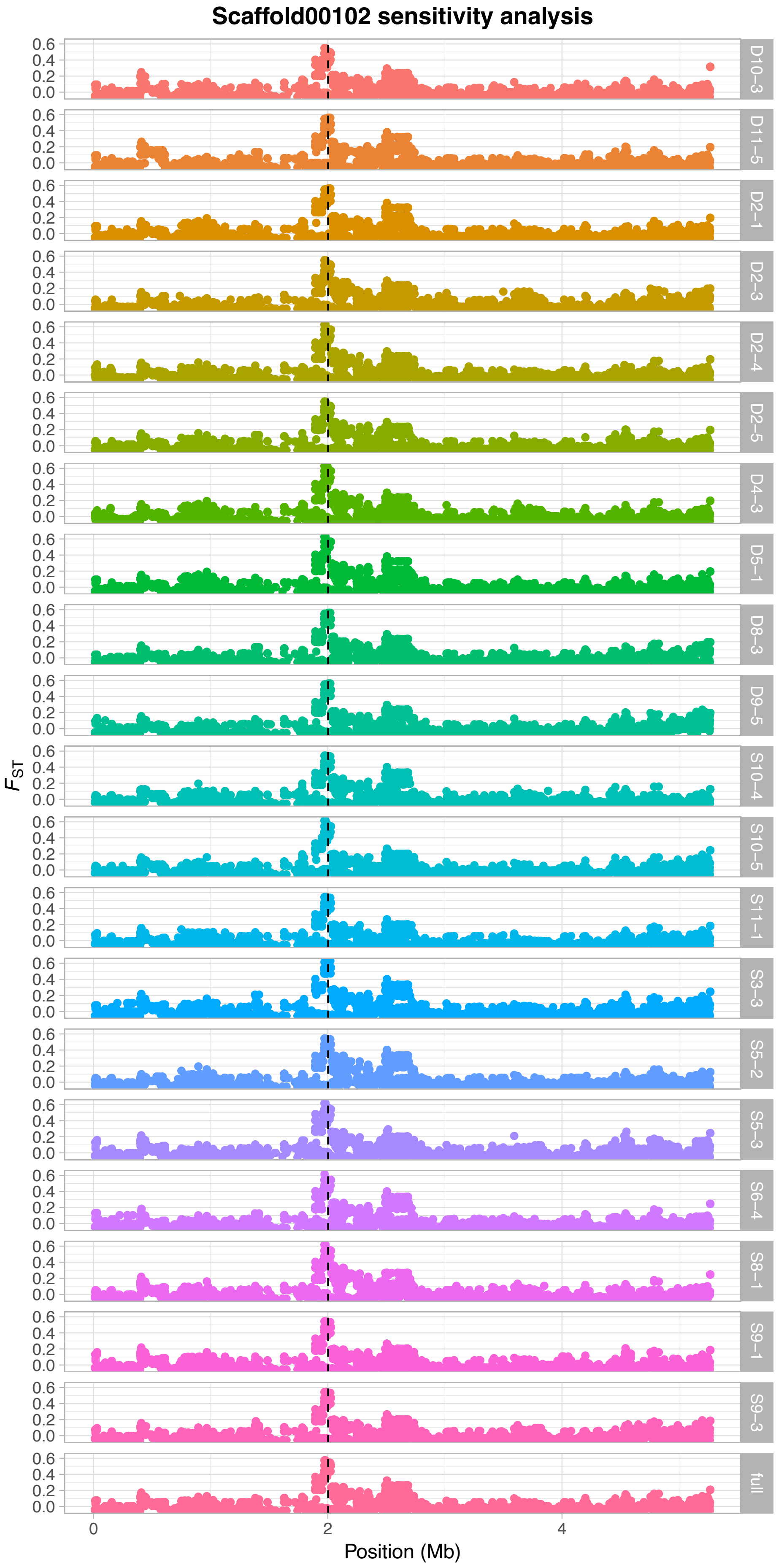

Scaffold00102 sensitivity analysis
D10–3
D11–5
D2–1
D2–3
D2–4
D2–5
D4–3
D5–1
D8–3
D9–5
S10–4
S10–5
S11–1
S3–3
S5–2
S5–3
S6–4
S8–1
S9–1
S9–3
full
F_{ST}
0.6
0.4
0.2
0.0
0
2
4
Position (Mb)

Figure S7 ***Sensitivity analysis of F_{ST} outlier peak on scaffold00102.***

The robustness of the F_{ST} outlier signal was evaluated by excluding one individual at a time and recalculating F_{ST} to see if it altered the results. The dashed line is the position of the highest F_{ST} (F_{ST} = 0.57 at position 1 971 662) value on the scaffold and the name of the excluded individual is displayed in the facet. The full dataset is plotted at the bottom of the figure. The signal is robust and is maintained in all scenarios.

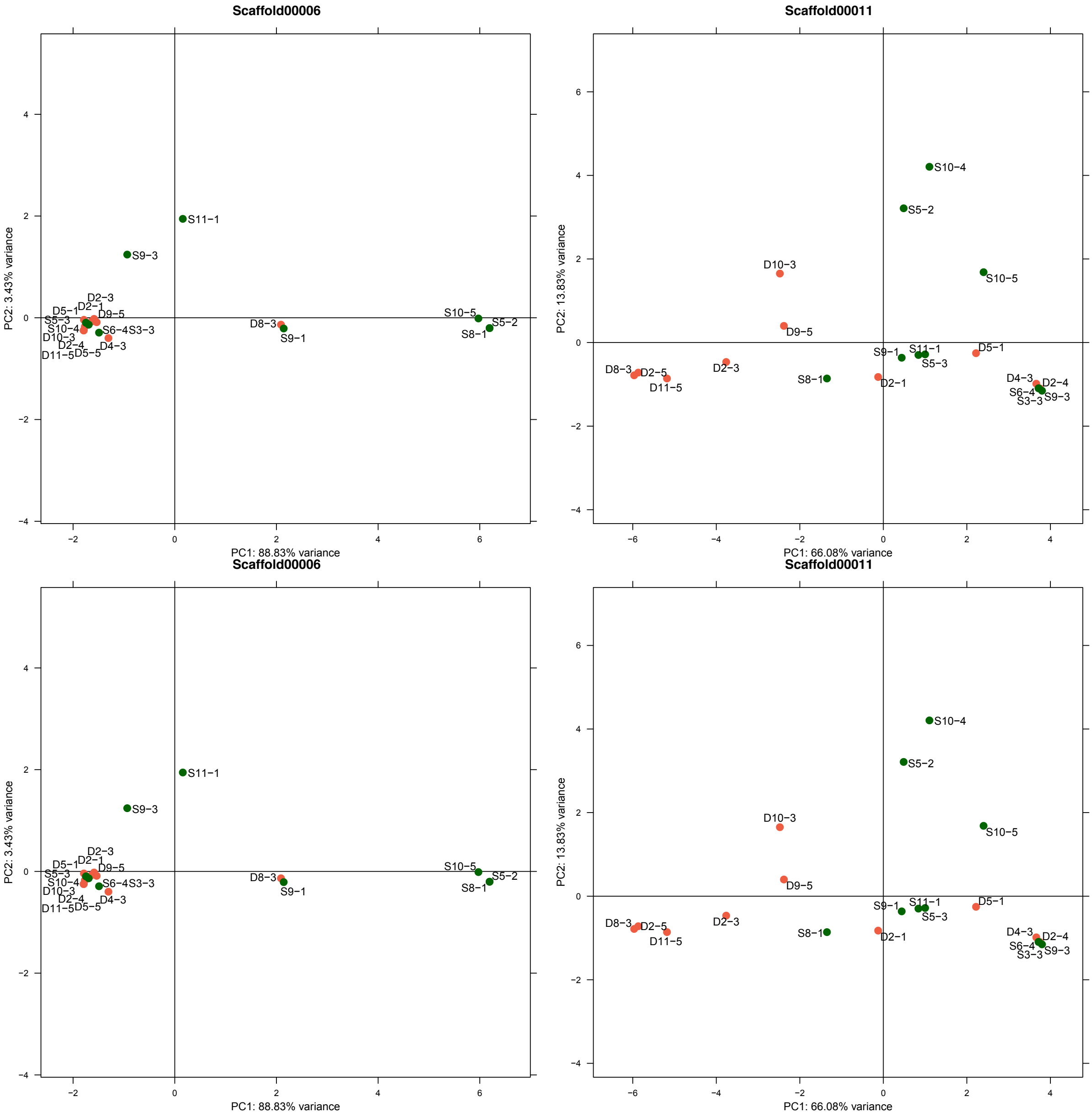

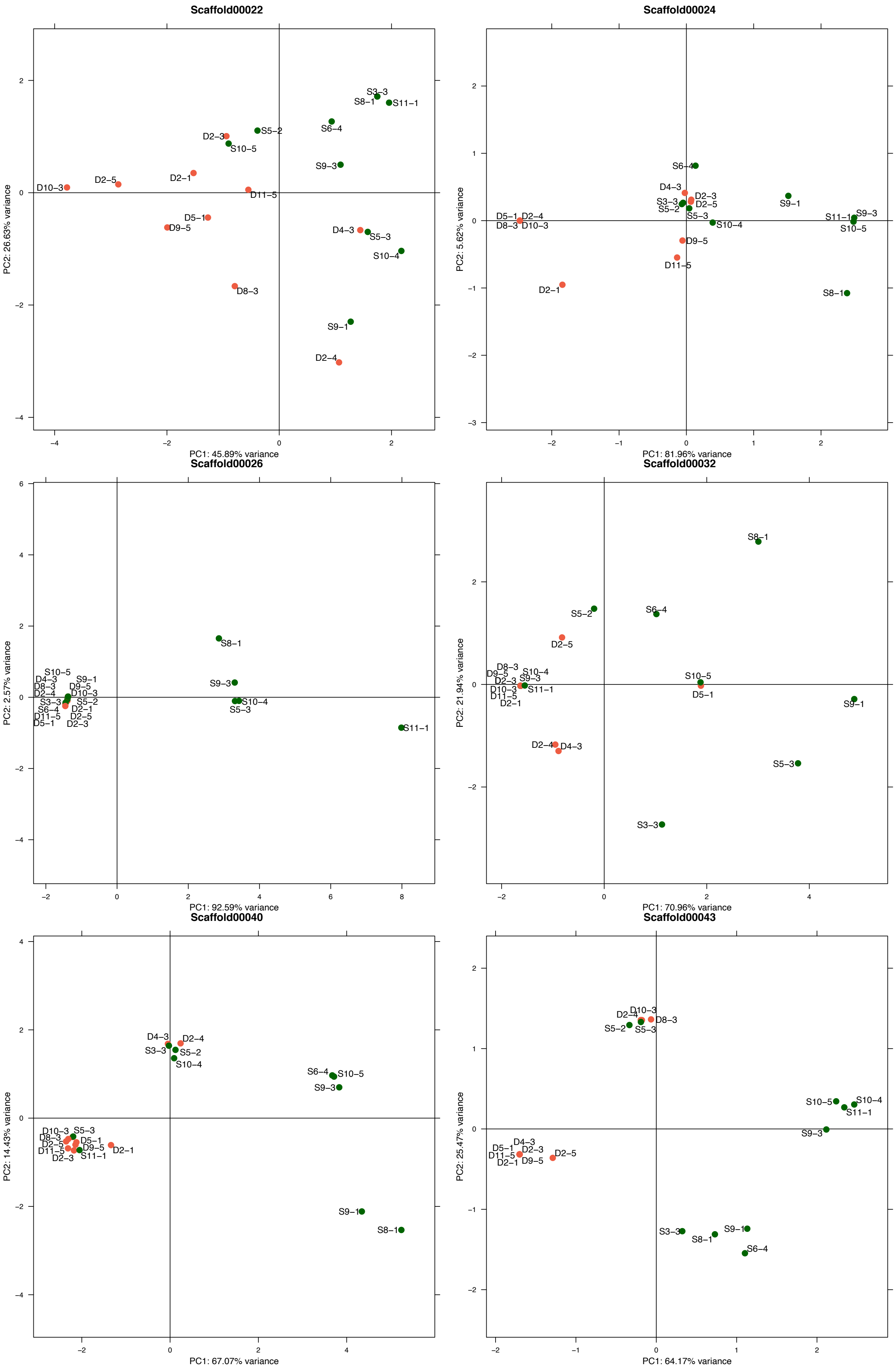
Scaffold00022
PC1: 45.89% variance
PC2: 26.63% variance
Scaffold00024
PC1: 81.96% variance
PC2: 5.62% variance
Scaffold00026
PC1: 92.59% variance
PC2: 2.57% variance
Scaffold00032
PC1: 70.96% variance
PC2: 21.94% variance
Scaffold00040
PC1: 67.07% variance
PC2: 14.43% variance
Scaffold00043
PC1: 64.17% variance
PC2: 25.47% variance

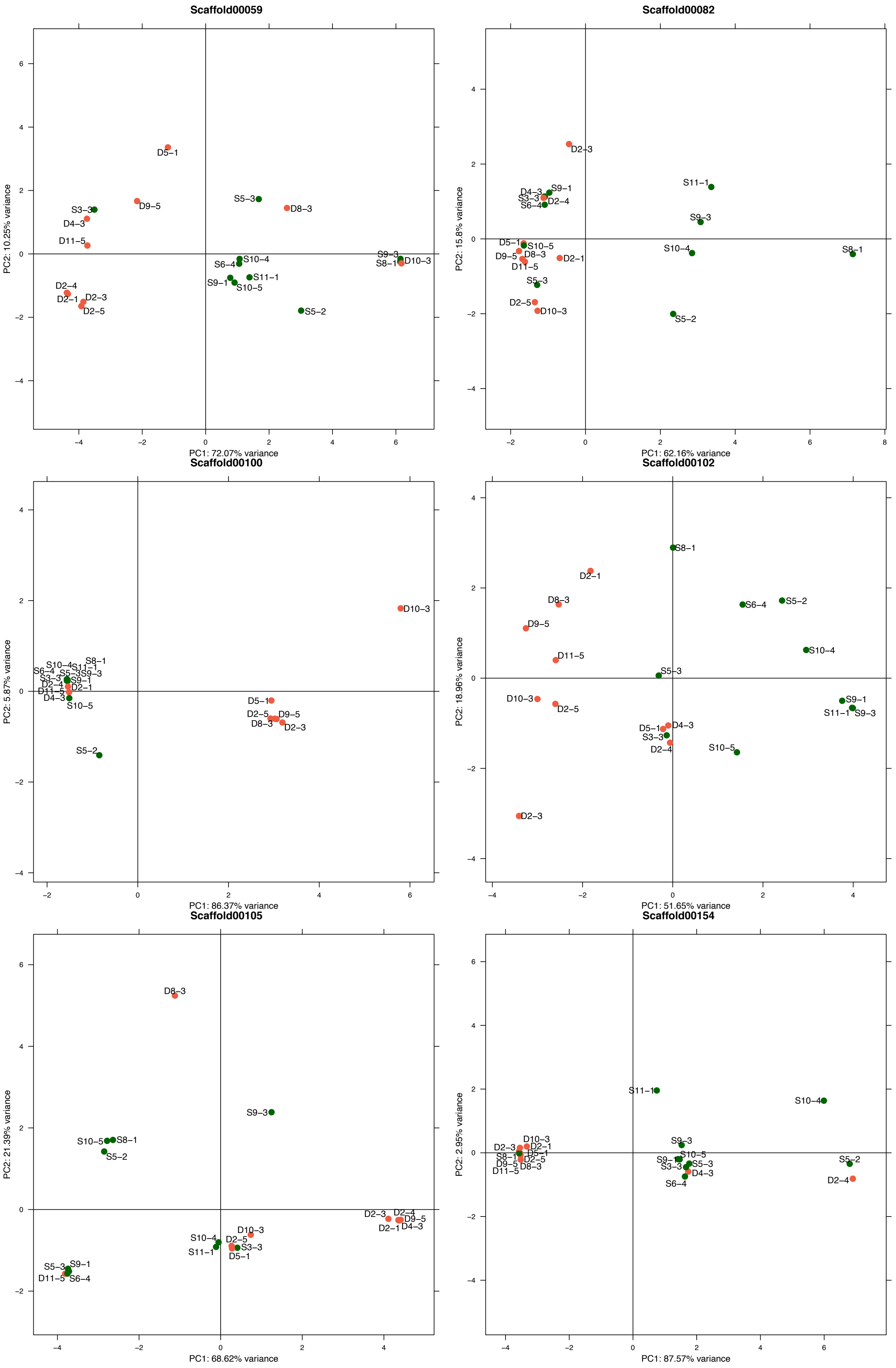
Scaffold00059
PC1: 72.07% variance
PC2: 10.25% variance
Scaffold00082
PC1: 62.16% variance
PC2: 15.8% variance
Scaffold00100
PC1: 86.37% variance
PC2: 5.87% variance
Scaffold00102
PC1: 51.65% variance
PC2: 18.96% variance
Scaffold00105
PC1: 68.62% variance
PC2: 21.39% variance
Scaffold00154
PC1: 87.57% variance
PC2: 2.95% variance

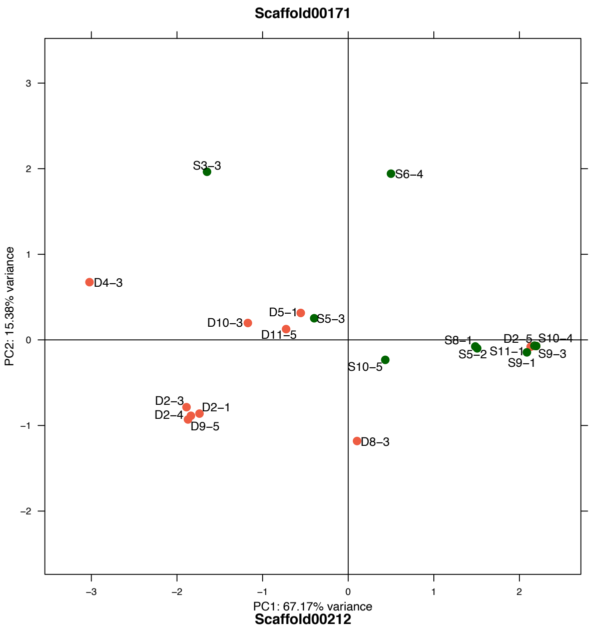
Scaffold00171
PC1: 67.17% variance
PC2: 15.38% variance
S3–3
S6–4
D4–3
D5–1
S5–3
D10–3
D11–5
S8–1
D2–5
S10–4
S5–2
S11–1
S9–3
S9–1
S10–5
D2–3
D2–1
D2–4
D9–5
D8–3

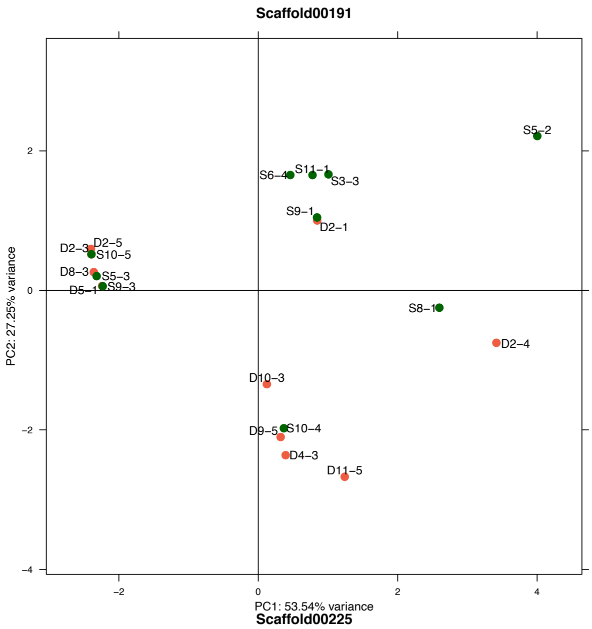
Scaffold00191
PC1: 53.54% variance
PC2: 27.25% variance
S5–2
S11–1
S6–4
S3–3
S9–1
D2–1
D2–3
D2–5
S10–5
D8–3
S5–3
D5–1
S9–3
S8–1
D2–4
D10–3
D9–5
S10–4
D4–3
D11–5

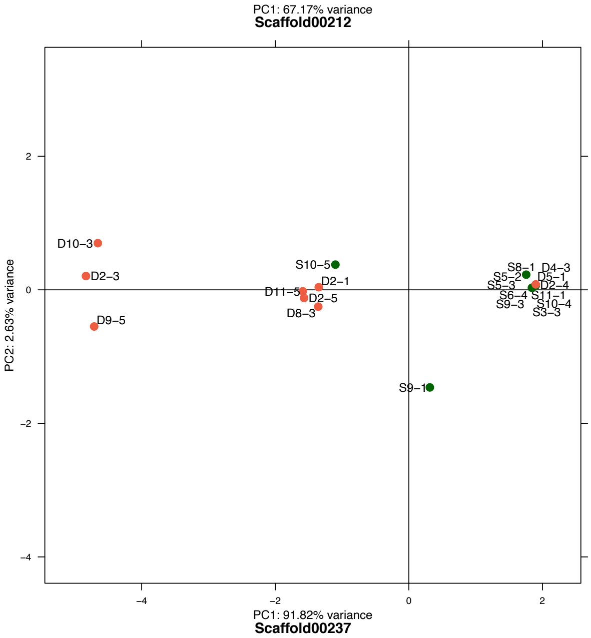
Scaffold00212
PC1: 91.82% variance
PC2: 2.63% variance
D10–3
D2–3
D9–5
S10–5
D2–1
D11–5
D2–5
D8–3
S8–1
D4–3
S5–2
D5–1
S5–3
D2–4
S6–4
S11–1
S9–3
S10–4
S3–3
S9–1

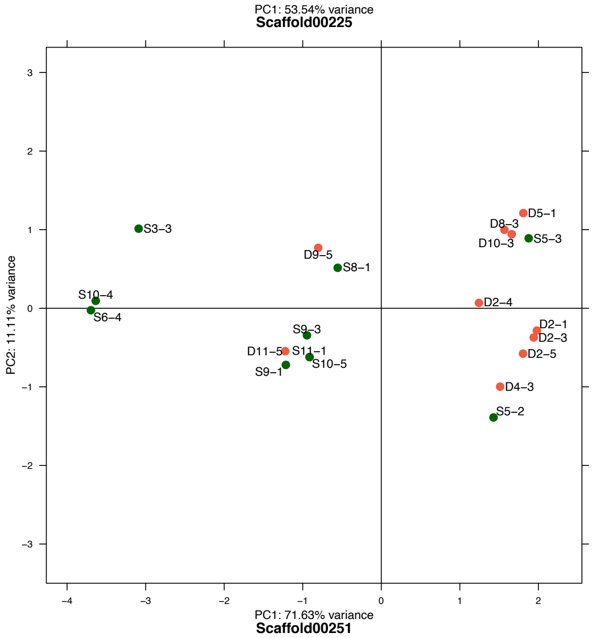
Scaffold00225
PC1: 71.63% variance
PC2: 11.11% variance
S3–3
D9–5
S8–1
D8–3
D5–1
D10–3
S5–3
S10–4
S6–4
D2–4
S9–3
D11–5
S11–1
S10–5
S9–1
D2–1
D2–3
D2–5
D4–3
S5–2

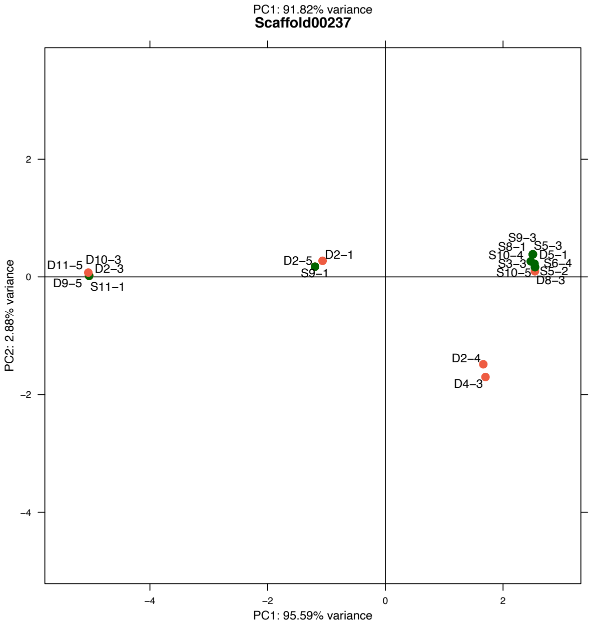
Scaffold00237
PC1: 95.59% variance
PC2: 2.88% variance
D11–5
D10–3
D2–3
D9–5
S11–1
D2–5
D2–1
S9–1
S9–3
S8–1
S5–3
S10–4
D5–1
S3–3
S6–4
S10–5
S5–2
D8–3
D2–4
D4–3

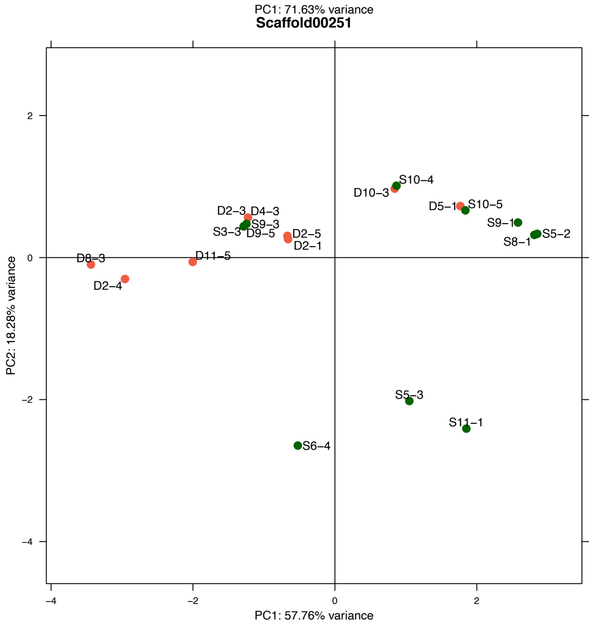
Scaffold00251
PC1: 57.76% variance
PC2: 18.28% variance
S10–4
D10–3
D5–1
S10–5
S9–1
S5–2
S8–1
D2–3
D4–3
S3–3
S9–3
D9–5
D2–5
D2–1
D8–3
D11–5
D2–4
S5–3
S11–1
S6–4

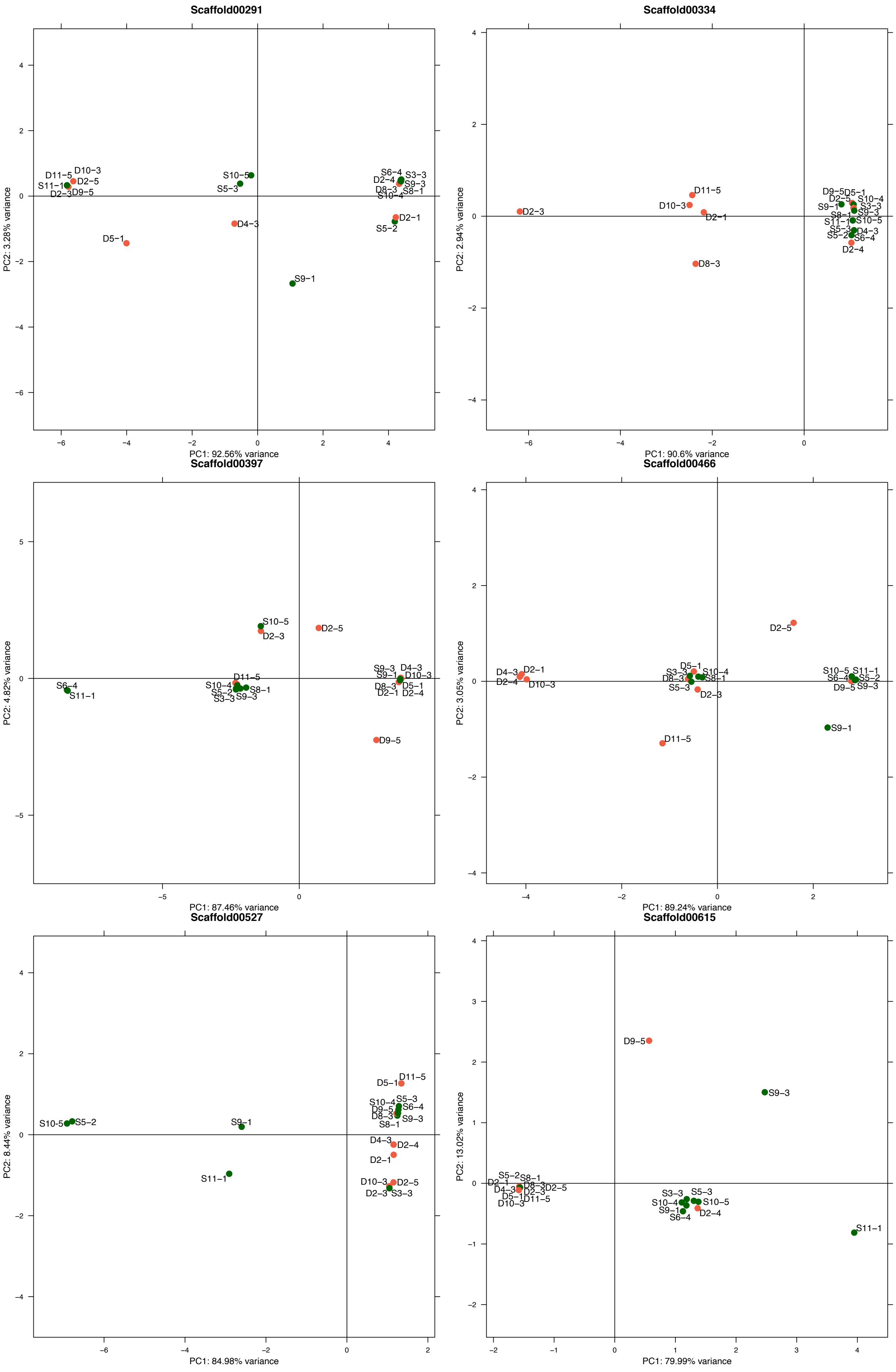
Scaffold00291
PC1: 92.56% variance
PC2: 3.28% variance
Scaffold00334
PC1: 90.6% variance
PC2: 2.94% variance
Scaffold00397
PC1: 87.46% variance
PC2: 4.82% variance
Scaffold00466
PC1: 89.24% variance
PC2: 3.05% variance
Scaffold00527
PC1: 84.98% variance
PC2: 8.44% variance
Scaffold00615
PC1: 79.99% variance
PC2: 13.02% variance

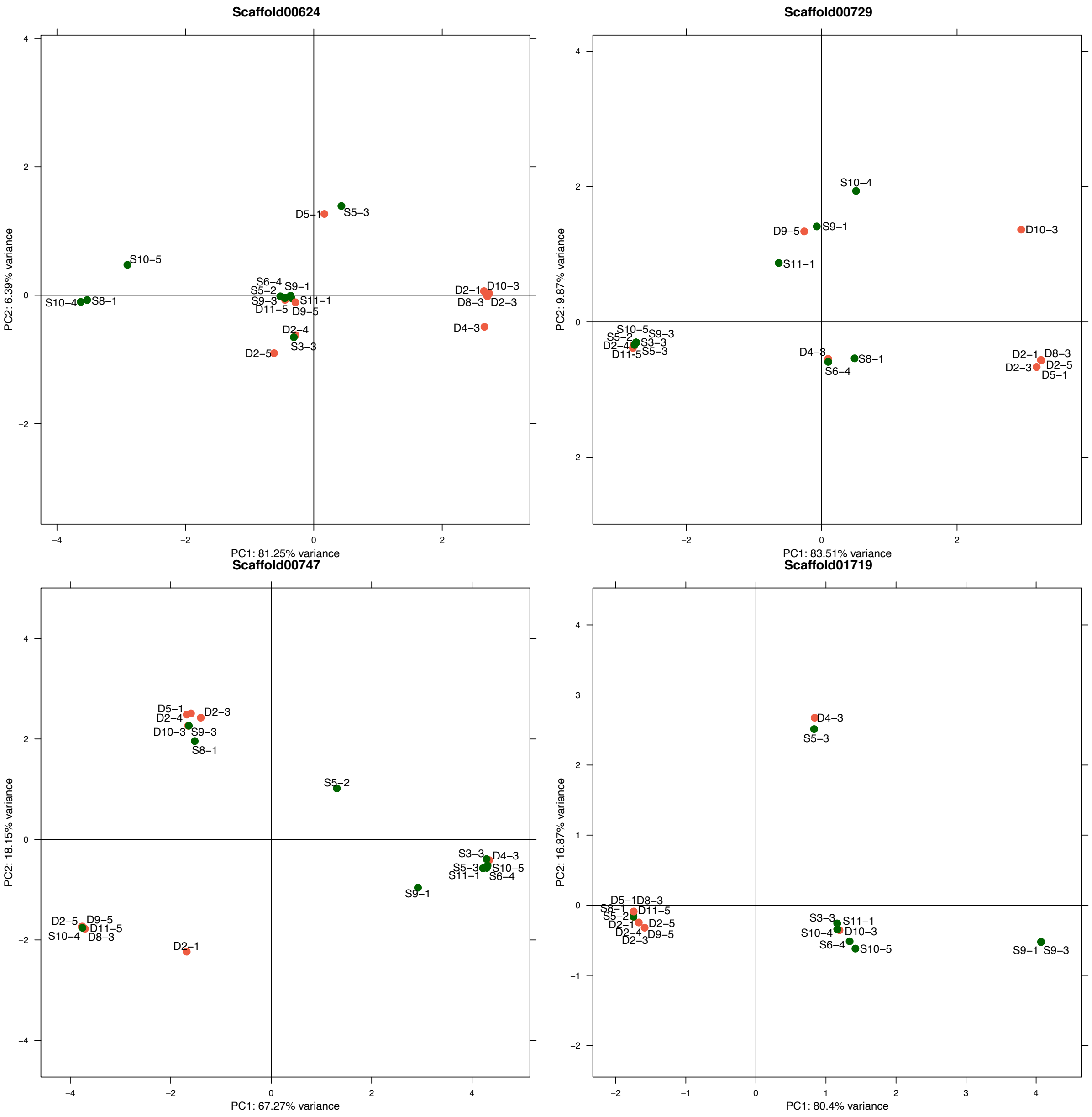

Figure S8. ***PCAs of the 30 scaffolds identified with regions of elevated F_{ST}.***
PCAs of the 30 identified scaffolds with elevated F_{ST}. Only SNPs found within each region of elevated F_{ST} was used to generate the PCAs. Several scaffolds show near complete separation with only a few individuals being placed within the other group, i.e. Scaffolds 22, 43, 59 and 102.

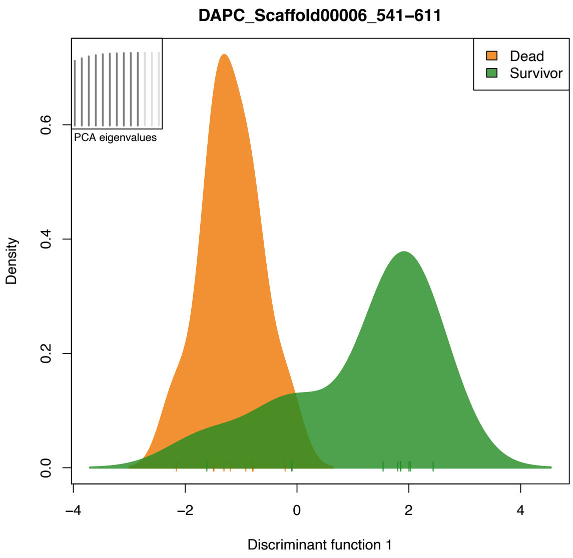
DAPC_Scaffold00006_541–611
PCA eigenvalues
Dead
Survivor
Density
0.0
0.2
0.4
0.6
−4
−2
0
2
4
Discriminant function 1

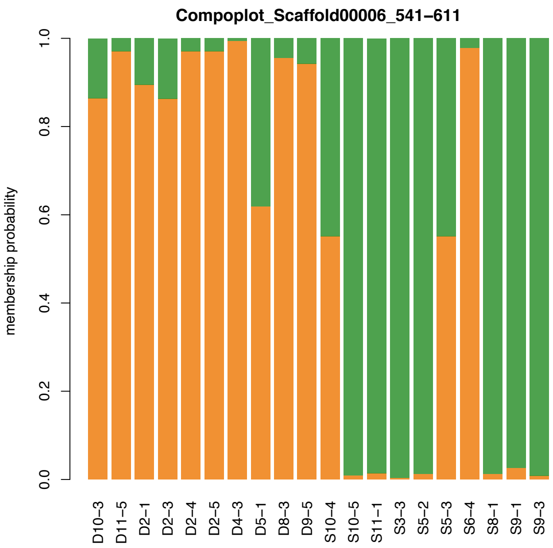
Compoplot_Scaffold00006_541–611
membership probability
0.0
0.2
0.4
0.6
0.8
1.0
D10−3 D11−5 D2−1 D2−3 D2−4 D2−5 D4−3 D5−1 D8−3 D9−5 S10−4 S10−5 S11−1 S3−3 S5−2 S5−3 S6−4 S8−1 S9−1 S9−3

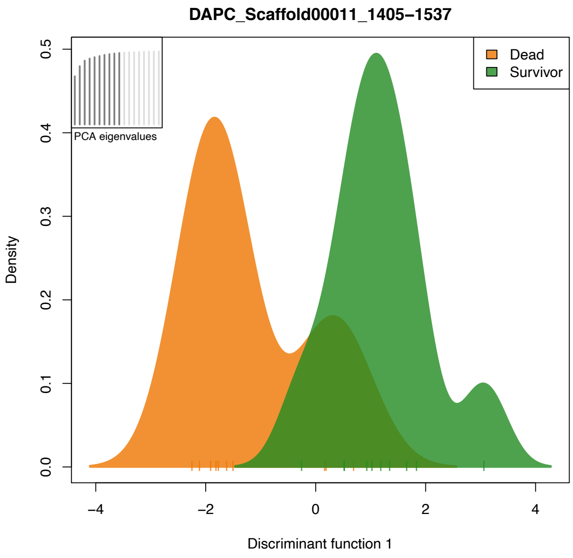
DAPC_Scaffold00011_1405–1537
PCA eigenvalues
Dead
Survivor
Density
0.0
0.1
0.2
0.3
0.4
0.5
−4
−2
0
2
4
Discriminant function 1

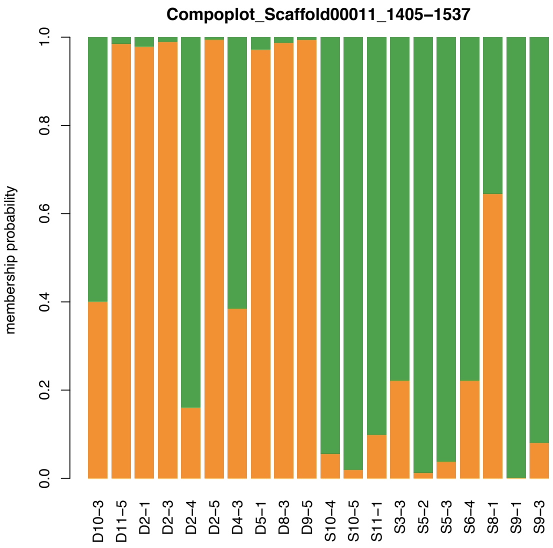
Compoplot_Scaffold00011_1405–1537
membership probability
0.0
0.2
0.4
0.6
0.8
1.0
D10−3 D11−5 D2−1 D2−3 D2−4 D2−5 D4−3 D5−1 D8−3 D9−5 S10−4 S10−5 S11−1 S3−3 S5−2 S5−3 S6−4 S8−1 S9−1 S9−3

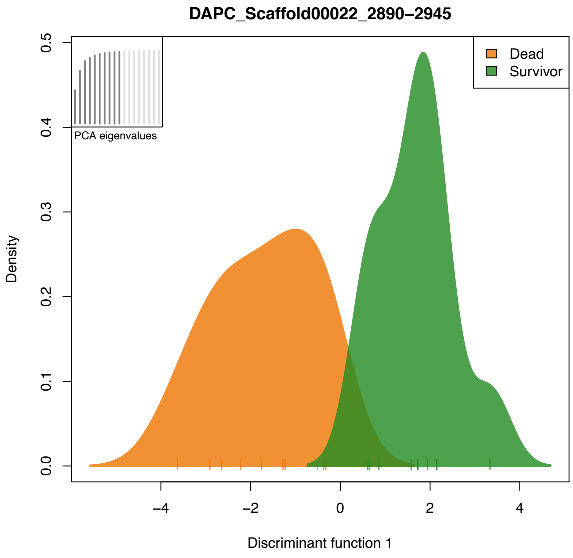
DAPC_Scaffold00022_2890–2945
PCA eigenvalues
Dead
Survivor
Density
0.0
0.1
0.2
0.3
0.4
0.5
−4
−2
0
2
4
Discriminant function 1

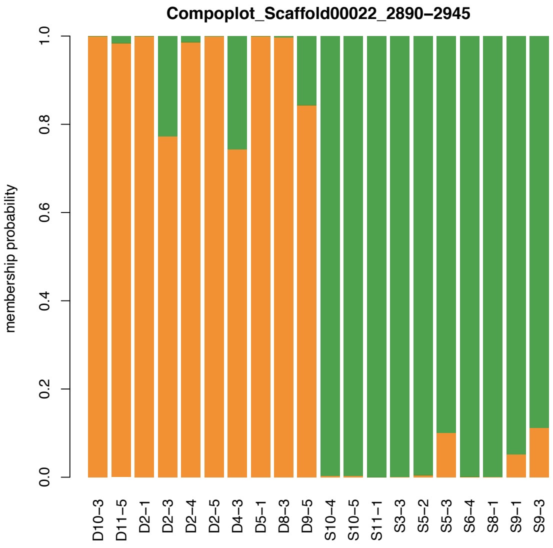
Compoplot_Scaffold00022_2890–2945
membership probability
0.0
0.2
0.4
0.6
0.8
1.0
D10−3 D11−5 D2−1 D2−3 D2−4 D2−5 D4−3 D5−1 D8−3 D9−5 S10−4 S10−5 S11−1 S3−3 S5−2 S5−3 S6−4 S8−1 S9−1 S9−3

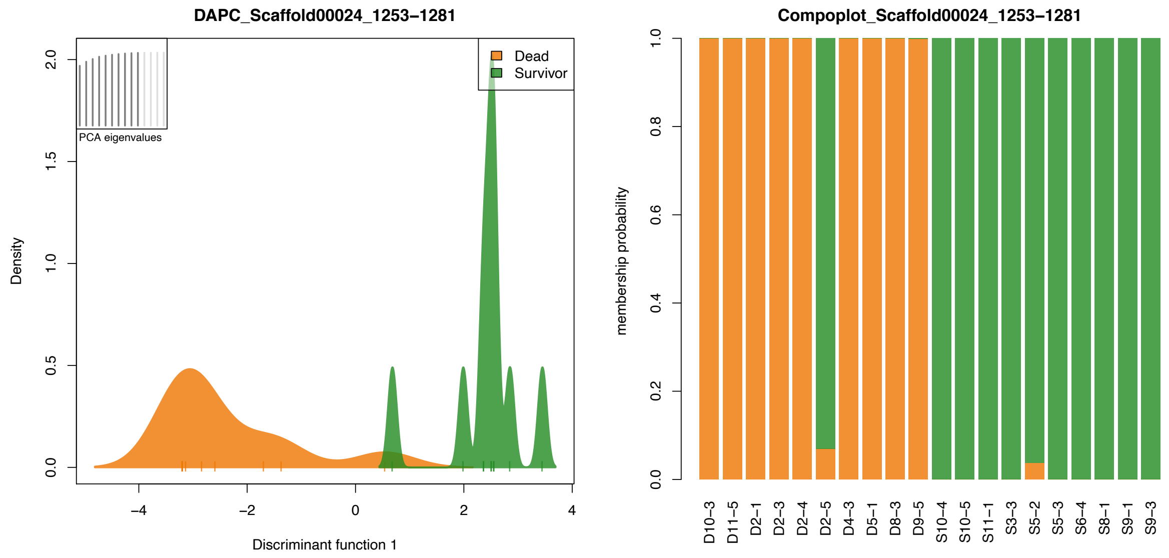
DAPC_Scaffold00024_1253–1281
Dead
Survivor
PCA eigenvalues
Density
Discriminant function 1
Compoplot_Scaffold00024_1253–1281
membership probability
D10–3 D11–5 D2–1 D2–3 D2–4 D2–5 D4–3 D5–1 D8–3 D9–5 S10–4 S10–5 S11–1 S3–3 S5–2 S5–3 S6–4 S8–1 S9–1 S9–3

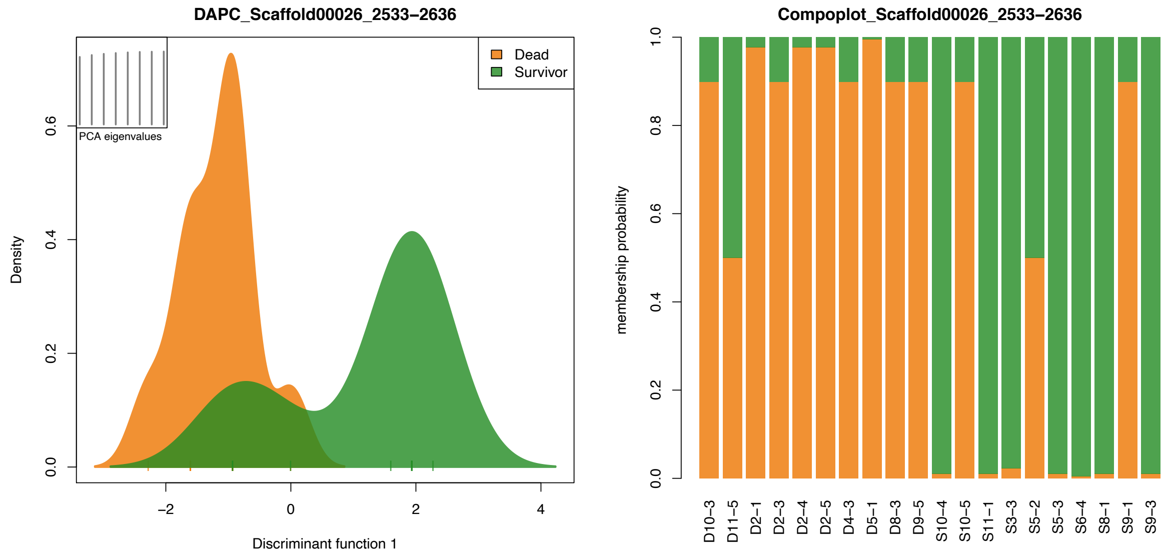
DAPC_Scaffold00026_2533–2636
Dead
Survivor
PCA eigenvalues
Density
Discriminant function 1
Compoplot_Scaffold00026_2533–2636
membership probability
D10–3 D11–5 D2–1 D2–3 D2–4 D2–5 D4–3 D5–1 D8–3 D9–5 S10–4 S10–5 S11–1 S3–3 S5–2 S5–3 S6–4 S8–1 S9–1 S9–3

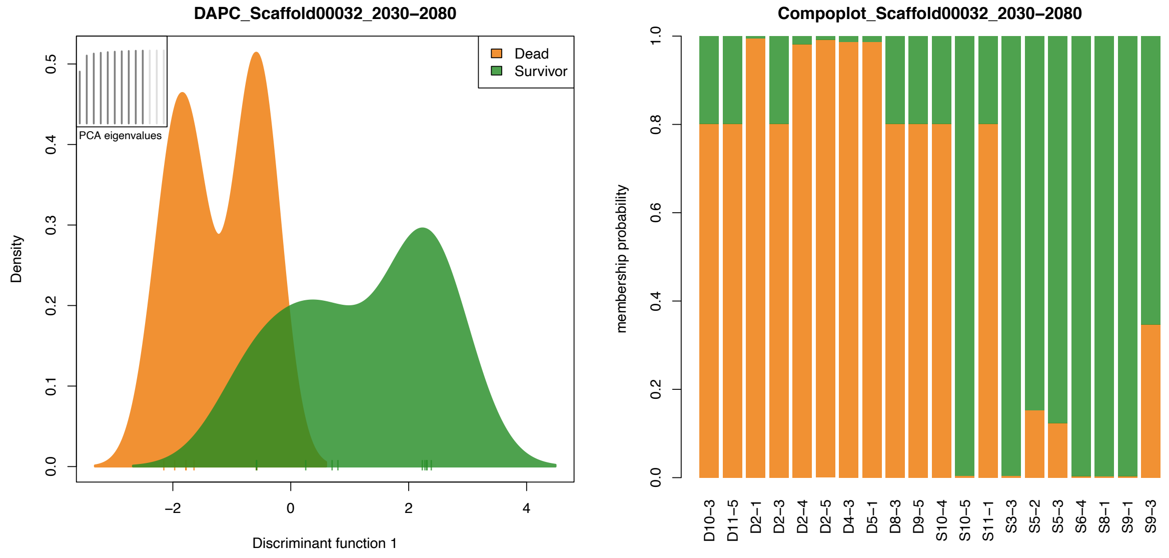
DAPC_Scaffold00032_2030–2080
Dead
Survivor
PCA eigenvalues
Density
Discriminant function 1
Compoplot_Scaffold00032_2030–2080
membership probability
D10–3 D11–5 D2–1 D2–3 D2–4 D2–5 D4–3 D5–1 D8–3 D9–5 S10–4 S10–5 S11–1 S3–3 S5–2 S5–3 S6–4 S8–1 S9–1 S9–3

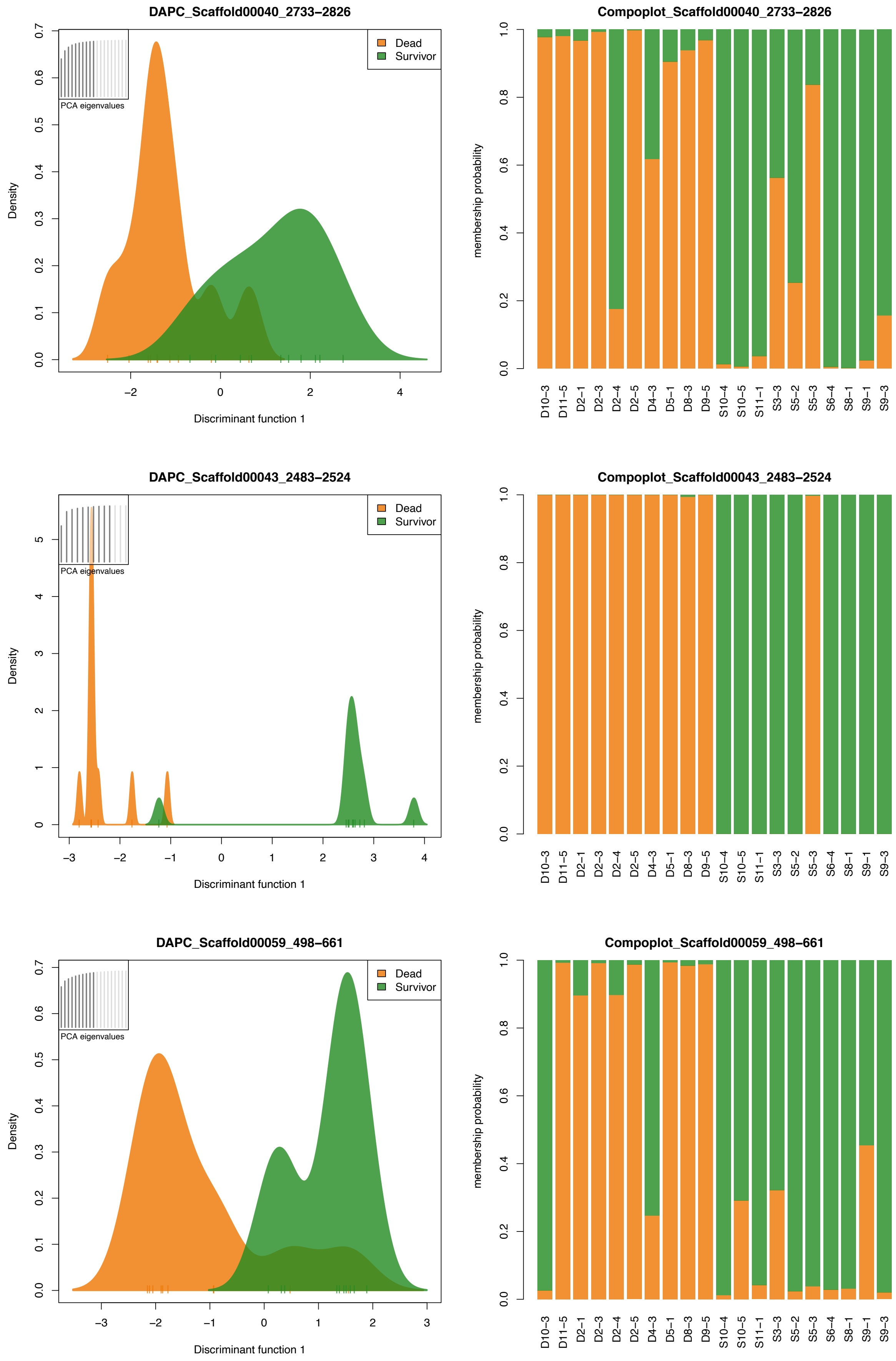
DAPC_Scaffold00040_2733–2826
Compoplot_Scaffold00040_2733–2826
DAPC_Scaffold00043_2483–2524
Compoplot_Scaffold00043_2483–2524
DAPC_Scaffold00059_498–661
Compoplot_Scaffold00059_498–661
PCA eigenvalues
Dead
Survivor
Density
Discriminant function 1
membership probability
D10–3
D11–5
D2–1
D2–3
D2–4
D2–5
D4–3
D5–1
D8–3
D9–5
S10–4
S10–5
S11–1
S3–3
S5–2
S5–3
S6–4
S8–1
S9–1
S9–3

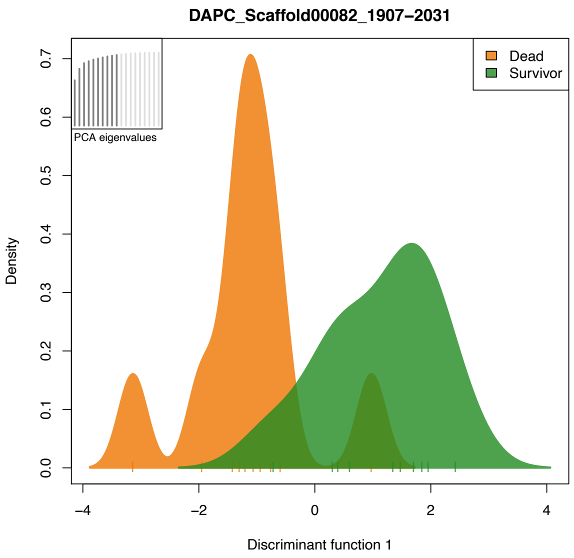
DAPC_Scaffold00082_1907–2031
PCA eigenvalues
Dead
Survivor
Density
Discriminant function 1

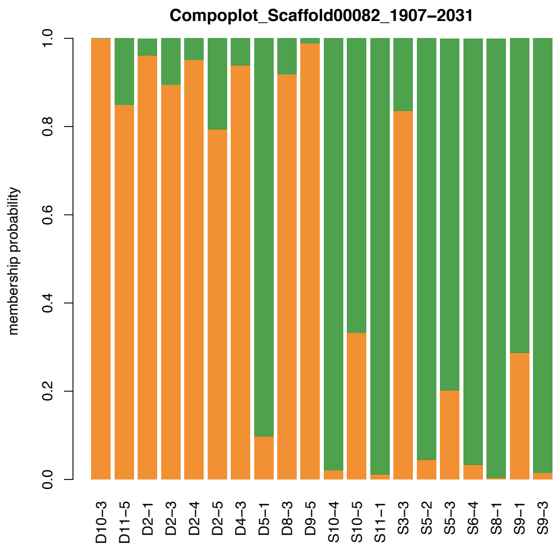
Compoplot_Scaffold00082_1907–2031
membership probability
D10–3 D11–5 D2–1 D2–3 D2–4 D2–5 D4–3 D5–1 D8–3 D9–5 S10–4 S10–5 S11–1 S3–3 S5–2 S5–3 S6–4 S8–1 S9–1 S9–3

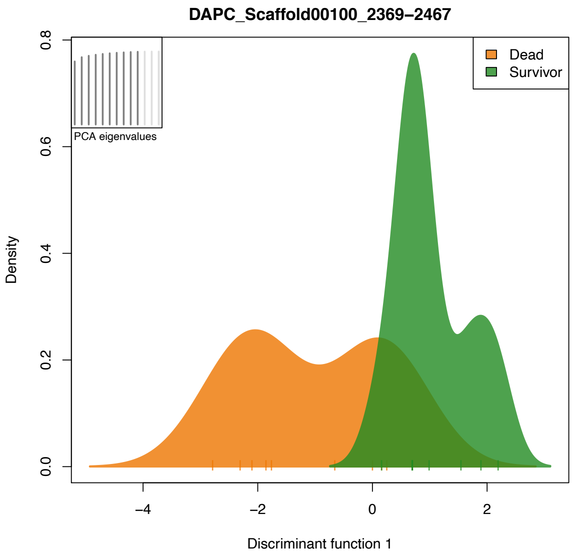
DAPC_Scaffold00100_2369–2467
PCA eigenvalues
Dead
Survivor
Density
Discriminant function 1

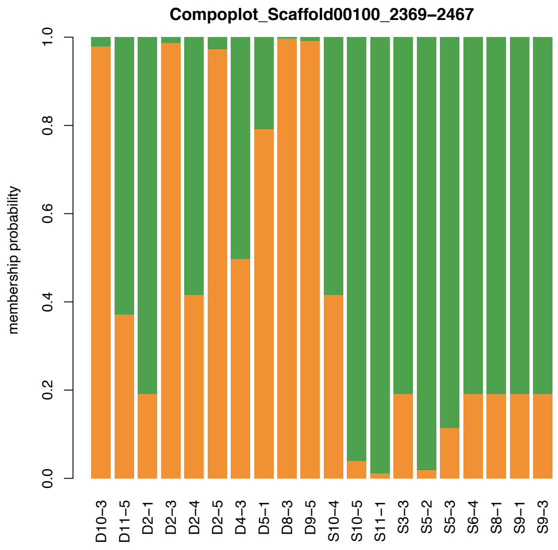
Compoplot_Scaffold00100_2369–2467
membership probability
D10–3 D11–5 D2–1 D2–3 D2–4 D2–5 D4–3 D5–1 D8–3 D9–5 S10–4 S10–5 S11–1 S3–3 S5–2 S5–3 S6–4 S8–1 S9–1 S9–3

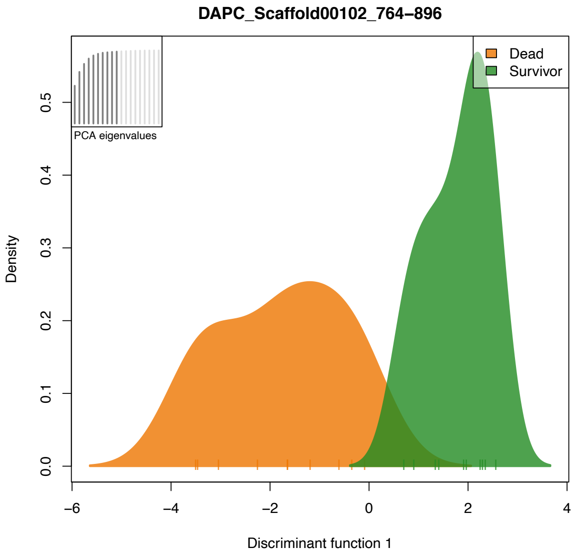
DAPC_Scaffold00102_764–896
PCA eigenvalues
Dead
Survivor
Density
Discriminant function 1

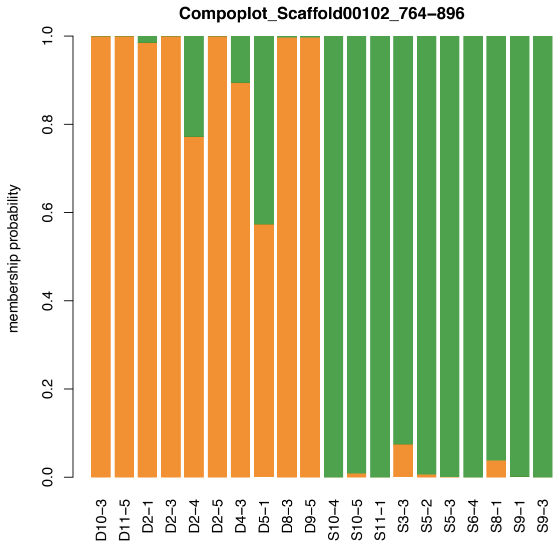
Compoplot_Scaffold00102_764–896
membership probability
D10–3 D11–5 D2–1 D2–3 D2–4 D2–5 D4–3 D5–1 D8–3 D9–5 S10–4 S10–5 S11–1 S3–3 S5–2 S5–3 S6–4 S8–1 S9–1 S9–3

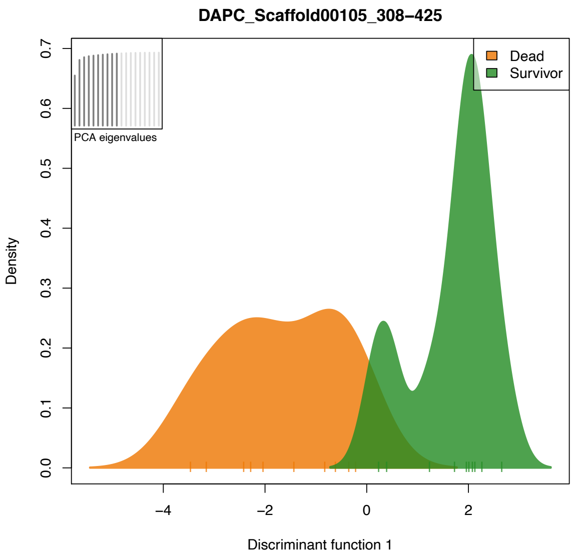
DAPC_Scaffold00105_308–425
PCA eigenvalues
Dead
Survivor
Density
Discriminant function 1

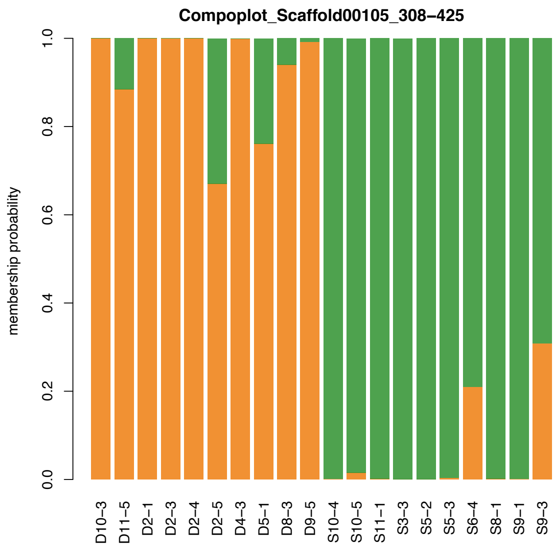
Compoplot_Scaffold00105_308–425
membership probability
D10–3
D11–5
D2–1
D2–3
D2–4
D2–5
D4–3
D5–1
D8–3
D9–5
S10–4
S10–5
S11–1
S3–3
S5–2
S5–3
S6–4
S8–1
S9–1
S9–3

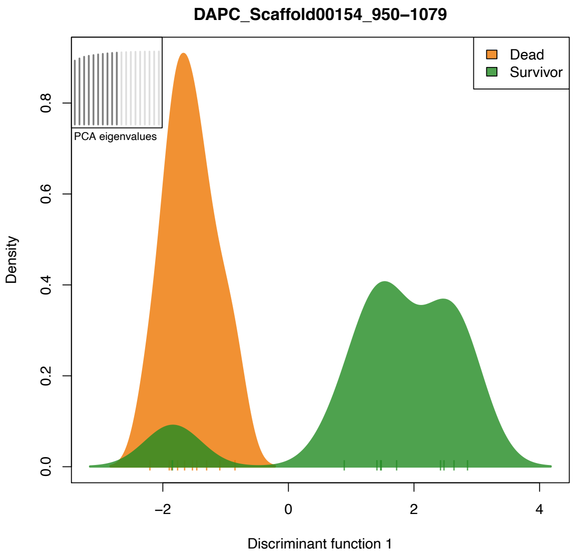
DAPC_Scaffold00154_950–1079
PCA eigenvalues
Dead
Survivor
Density
Discriminant function 1

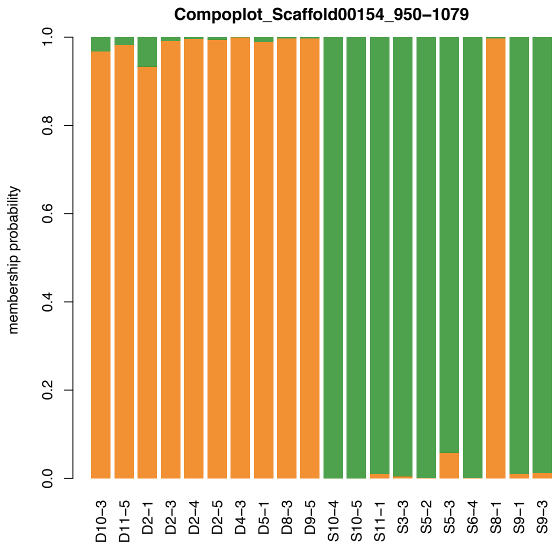
Compoplot_Scaffold00154_950–1079
membership probability
D10–3
D11–5
D2–1
D2–3
D2–4
D2–5
D4–3
D5–1
D8–3
D9–5
S10–4
S10–5
S11–1
S3–3
S5–2
S5–3
S6–4
S8–1
S9–1
S9–3

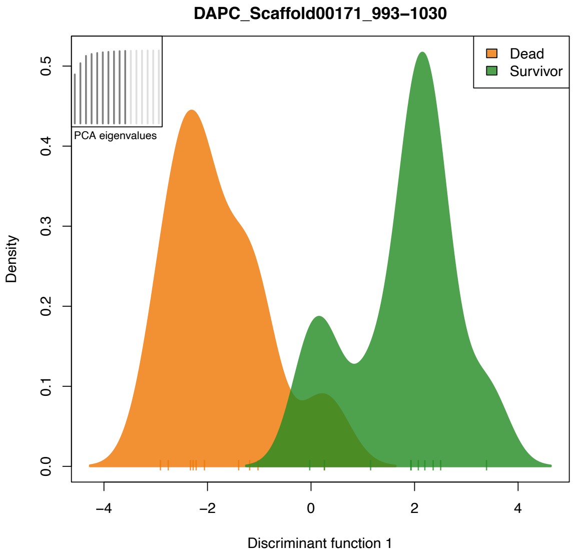
DAPC_Scaffold00171_993–1030
PCA eigenvalues
Dead
Survivor
Density
Discriminant function 1

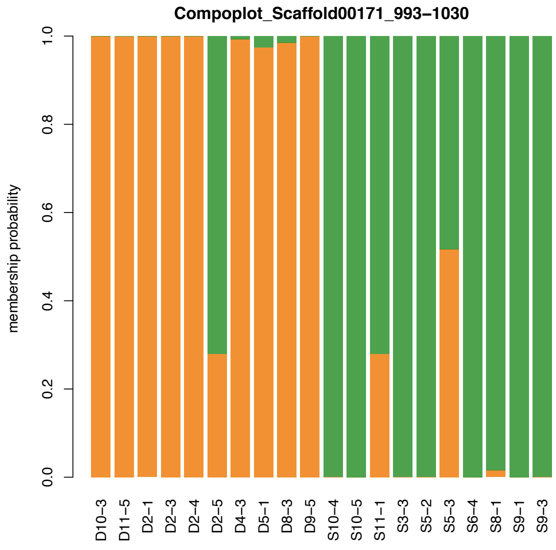
Compoplot_Scaffold00171_993–1030
membership probability
D10–3
D11–5
D2–1
D2–3
D2–4
D2–5
D4–3
D5–1
D8–3
D9–5
S10–4
S10–5
S11–1
S3–3
S5–2
S5–3
S6–4
S8–1
S9–1
S9–3

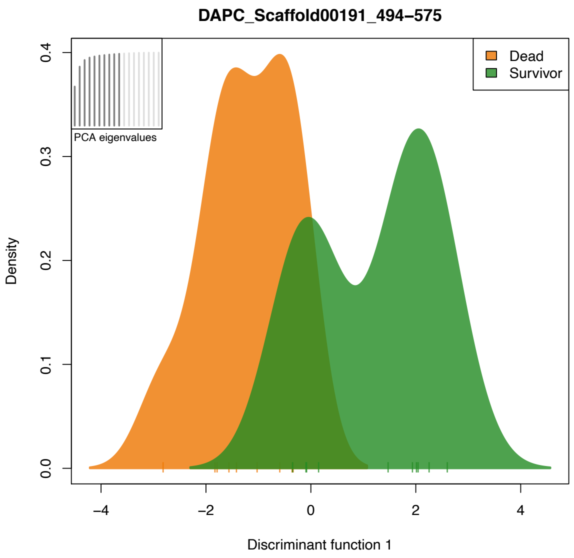
DAPC_Scaffold00191_494–575
Dead
Survivor
PCA eigenvalues
Density
Discriminant function 1

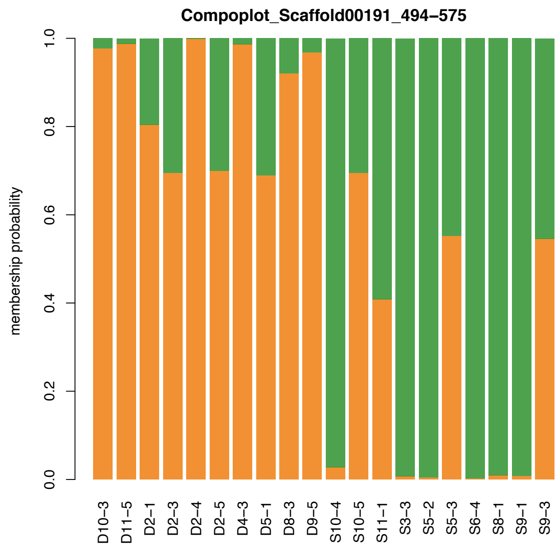
Compoplot_Scaffold00191_494–575
membership probability
D10–3
D11–5
D2–1
D2–3
D2–4
D2–5
D4–3
D5–1
D8–3
D9–5
S10–4
S10–5
S11–1
S3–3
S5–2
S5–3
S6–4
S8–1
S9–1
S9–3

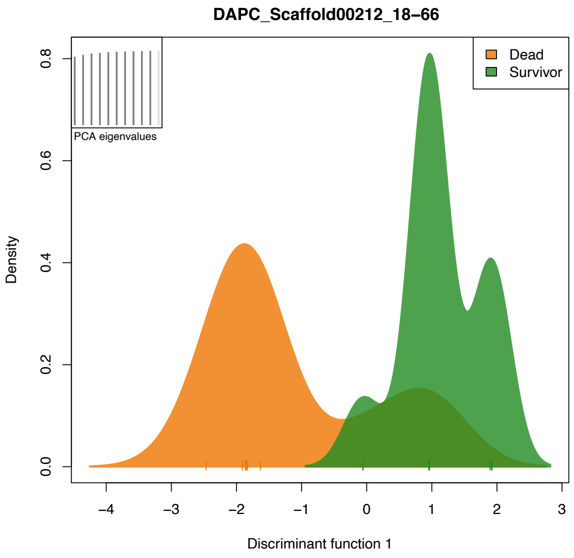
DAPC_Scaffold00212_18–66
Dead
Survivor
PCA eigenvalues
Density
Discriminant function 1

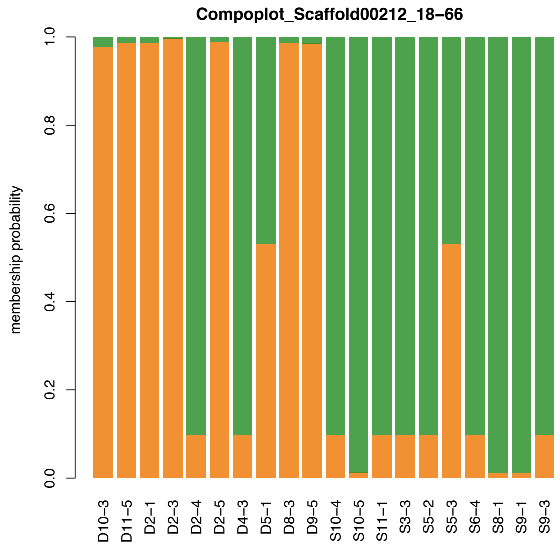
Compoplot_Scaffold00212_18–66
membership probability
D10–3
D11–5
D2–1
D2–3
D2–4
D2–5
D4–3
D5–1
D8–3
D9–5
S10–4
S10–5
S11–1
S3–3
S5–2
S5–3
S6–4
S8–1
S9–1
S9–3

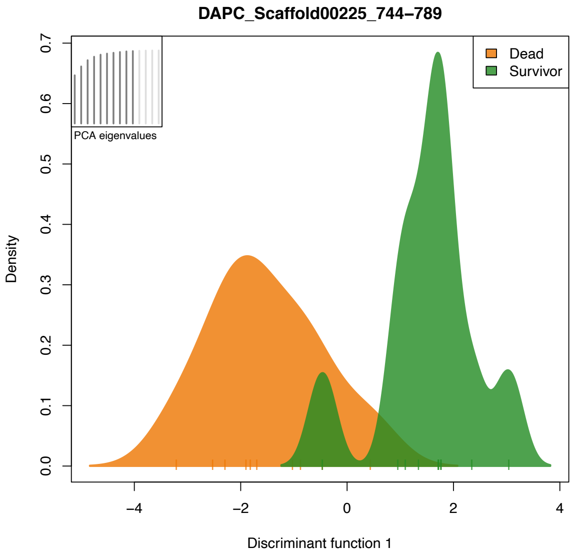
DAPC_Scaffold00225_744–789
Dead
Survivor
PCA eigenvalues
Density
Discriminant function 1

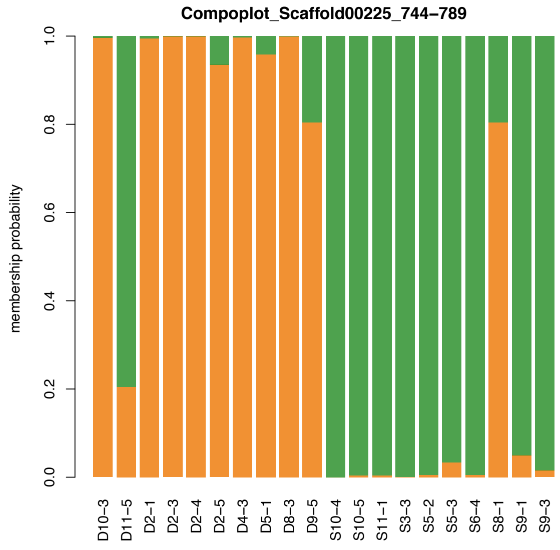
Compoplot_Scaffold00225_744–789
membership probability
D10–3
D11–5
D2–1
D2–3
D2–4
D2–5
D4–3
D5–1
D8–3
D9–5
S10–4
S10–5
S11–1
S3–3
S5–2
S5–3
S6–4
S8–1
S9–1
S9–3

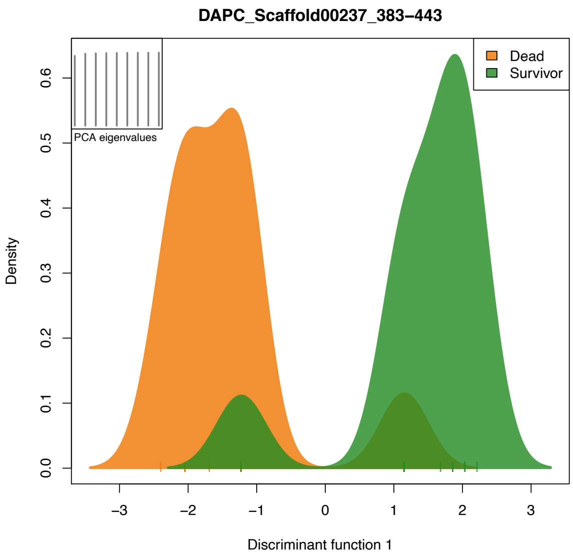
DAPC_Scaffold00237_383–443
Dead
Survivor
PCA eigenvalues
Density
Discriminant function 1

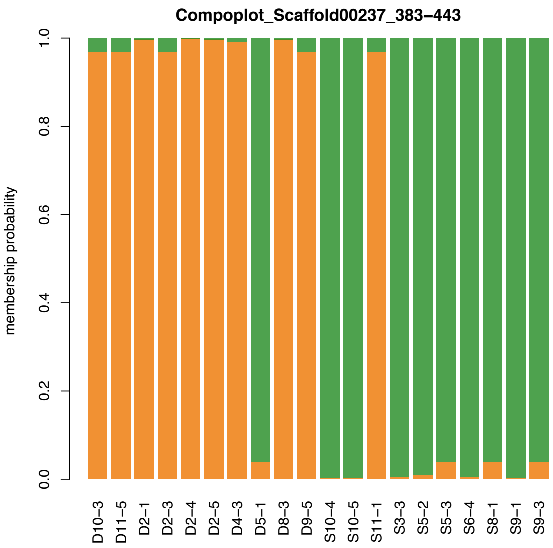
Compoplot_Scaffold00237_383–443
membership probability
D10–3 D11–5 D2–1 D2–3 D2–4 D2–5 D4–3 D5–1 D8–3 D9–5 S10–4 S10–5 S11–1 S3–3 S5–2 S5–3 S6–4 S8–1 S9–1 S9–3

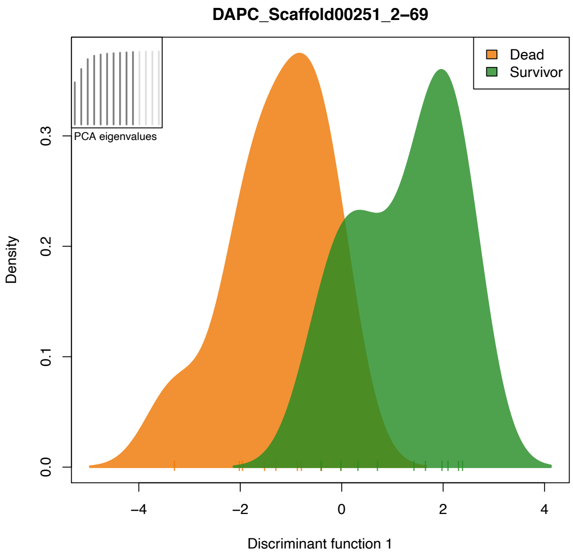
DAPC_Scaffold00251_2–69
Dead
Survivor
PCA eigenvalues
Density
Discriminant function 1

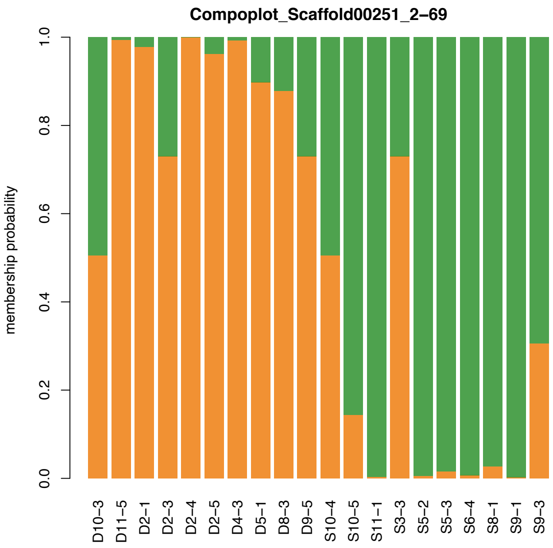
Compoplot_Scaffold00251_2–69
membership probability
D10–3 D11–5 D2–1 D2–3 D2–4 D2–5 D4–3 D5–1 D8–3 D9–5 S10–4 S10–5 S11–1 S3–3 S5–2 S5–3 S6–4 S8–1 S9–1 S9–3

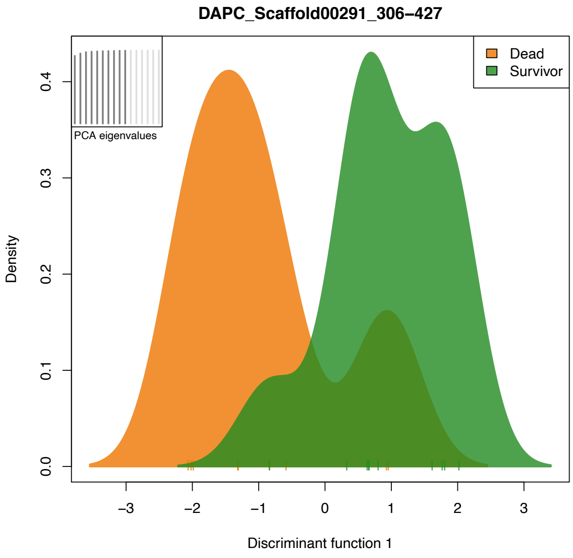
DAPC_Scaffold00291_306–427
Dead
Survivor
PCA eigenvalues
Density
Discriminant function 1

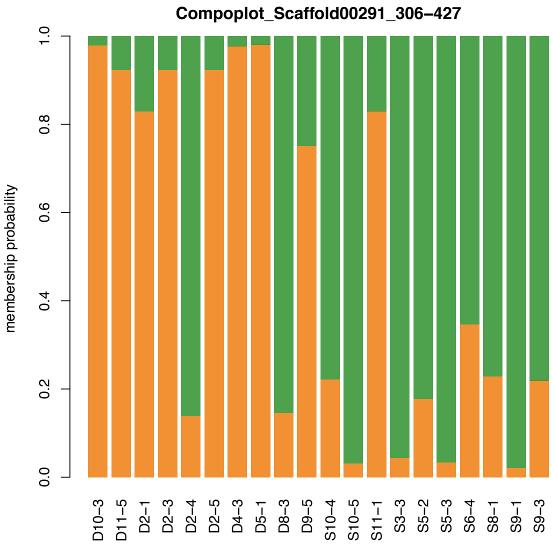
Compoplot_Scaffold00291_306–427
membership probability
D10–3 D11–5 D2–1 D2–3 D2–4 D2–5 D4–3 D5–1 D8–3 D9–5 S10–4 S10–5 S11–1 S3–3 S5–2 S5–3 S6–4 S8–1 S9–1 S9–3

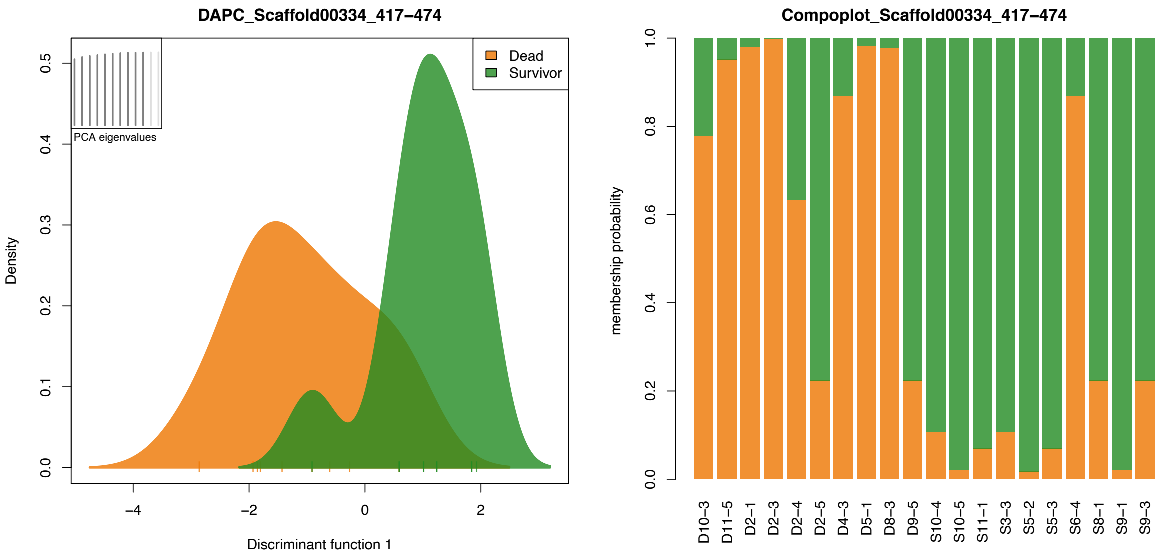

Figure S9. ***DAPCs and compoplots of the 21 scaffolds identified with regions of elevated F_{ST}.***

Only SNPs found within each region of elevated F_{ST} was used to generate the plots for each scaffold. Moribund individuals are depicted in orange and survivors in green.

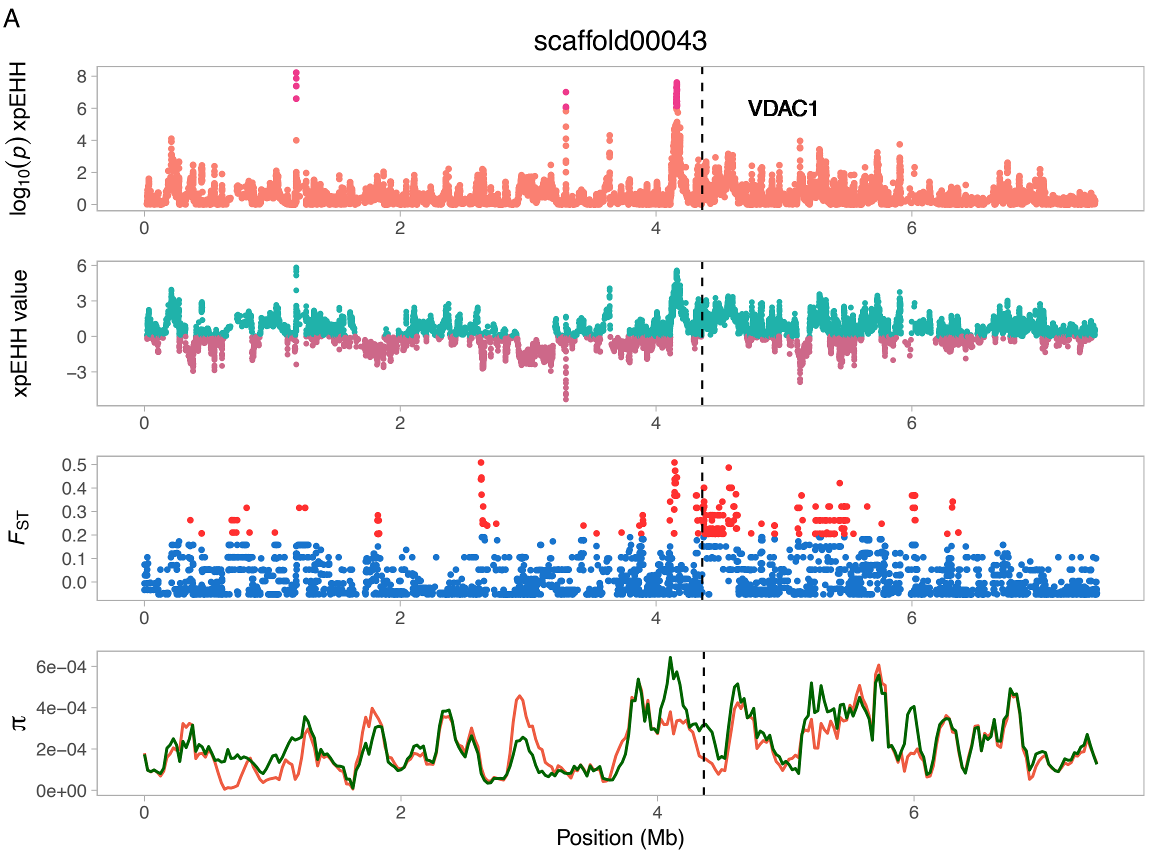

B

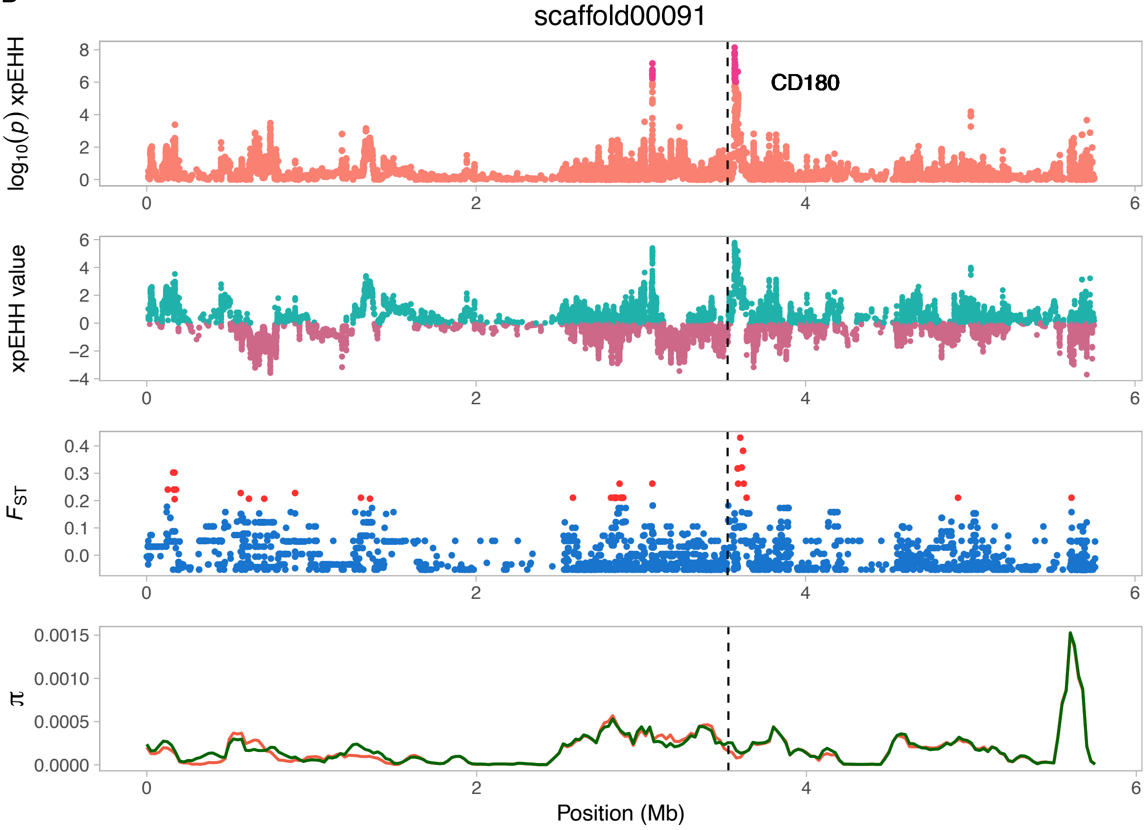
scaffold00091
CD180
log$_{10}$(p) xpEHH
xpEHH value
F_{ST}
π
Position (Mb)

C

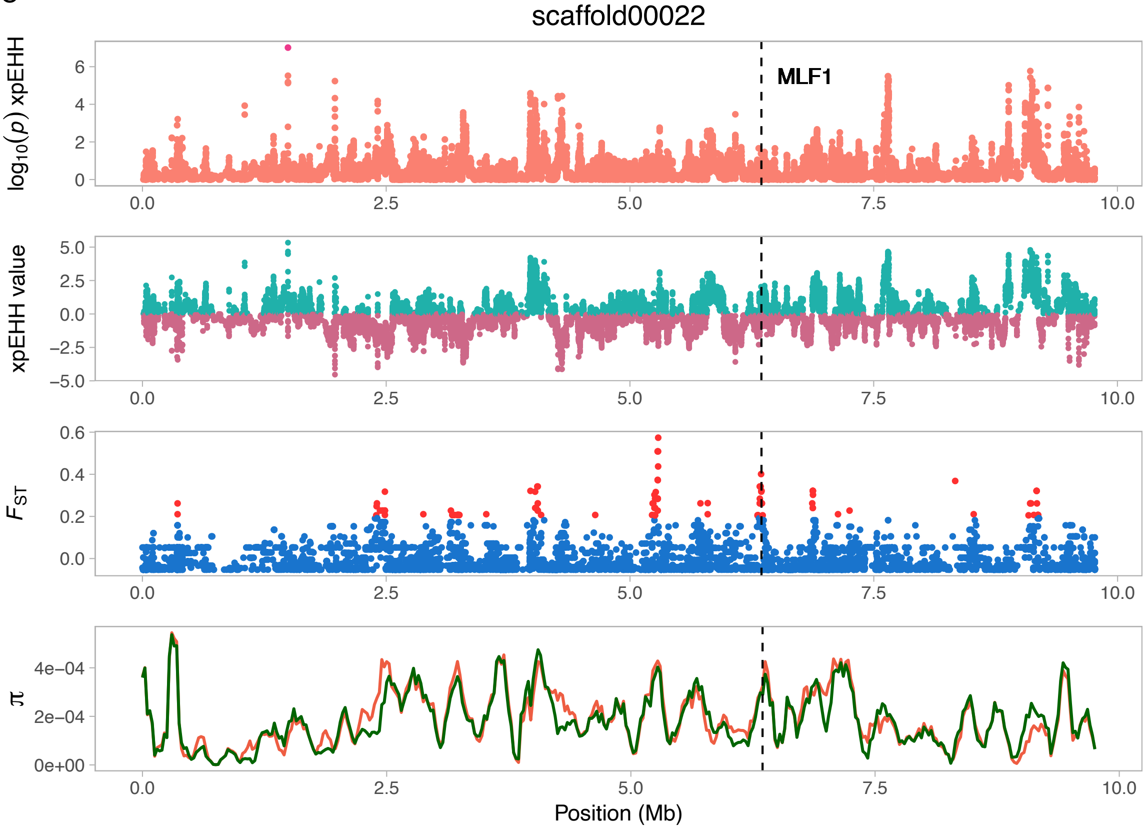
scaffold00022
MLF1
log$_{10}$(p) xpEHH
xpEHH value
F_{ST}
π
Position (Mb)

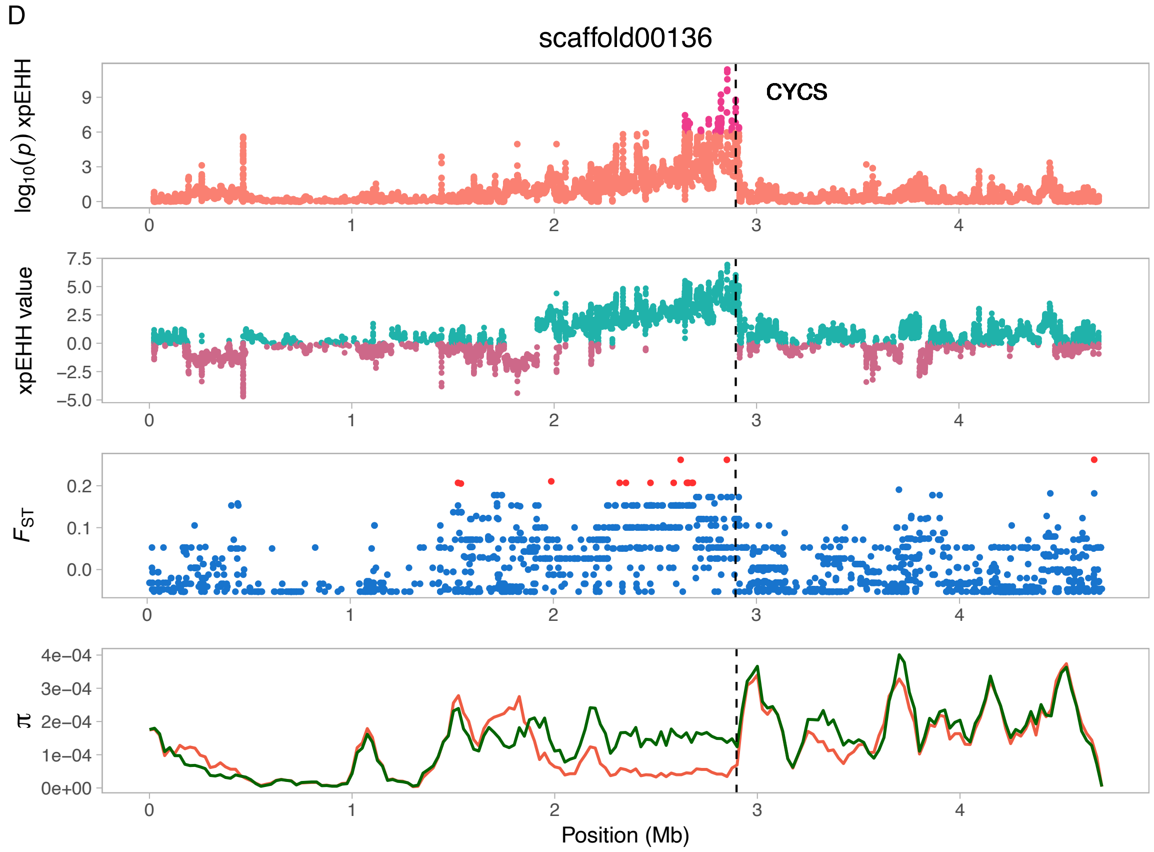

Figure S10. ***Signatures of recent selection, differentiation and nucleotide diversity along scaffolds 43 (A), 91 (B), 22 (C) and 136 (D).*** For each; Top panel – Plot of log10 (*p*) xpEHH, where high values indicate strong signals of selection (threshold of significance set at $\log_{10} 1 \times 10^{-6}$), second panel; Plot of xpEHH value where positive values (teal) indicate selection has occurred in surviving gerbils, while negative values (dark violet) indicate selection in dying gerbils, third panel; relative differentiation (F_{ST}) between moribund and survivors (red = SNPs with elevated values, $F_{ST} > 0.2$), fourth panel – nucleotide diversity (π) of moribund (orange) and survivors (green). Vertical dashed lines represent in (A) the midpoint of the *VDAC1* gene within a significant F_{ST} peak, and upstream of a significant xpEHH peak. In (B) the dashed line represents the midpoint of the *CD180* gene, just upstream of significant xpEHH and F_{ST} peaks. In (C) the location of the *MLF1* gene within a significant F_{ST} peak. For the gene positions in (C) and (D) the midpoint is used.

The positive xpEHH value at the position (and surrounding region) of the VDAC1 and CD180 genes indicate that the selection has occurred in the survivors.

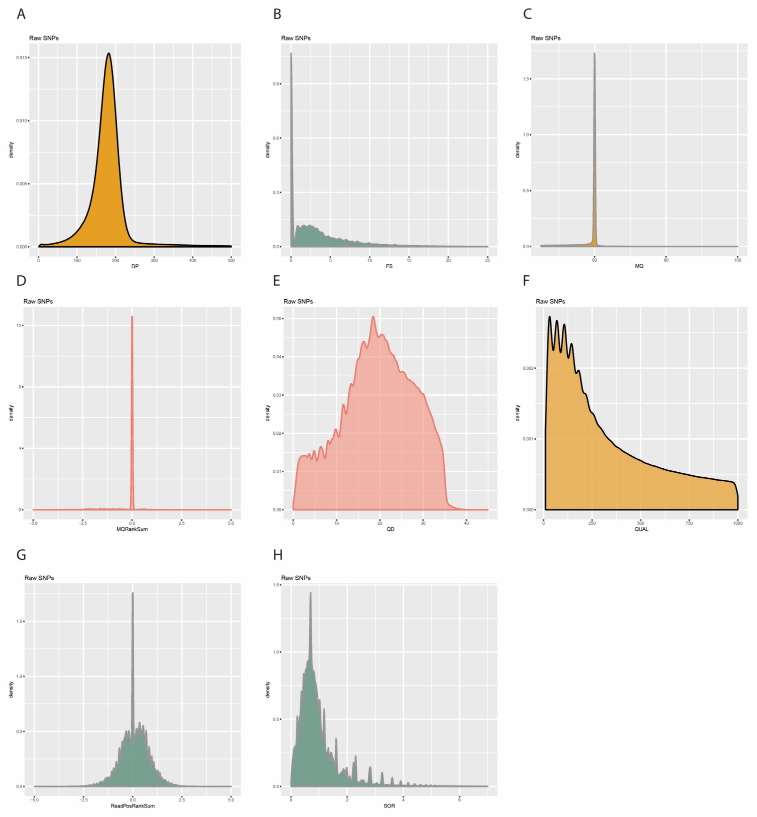

Figure S11. ***GATK quality metrics of the raw SNP set.***
The annotation values of SNPs were extracted from the raw vcf file using GATKs -T VariantsToTable flag and density plots for each quality metric was generated using the ggplot2 package in R to determine cut-off values for the hard filtering of SNPs. (A) DP, (B) Fisher strand (FS), (C) MQ, (D) MQRankSum, (E) Quality by depth (QD), (F) QUAL, (G) ReadPosRankSum and (H) SOR. The thresholds used on the gerbil data set were as follows: FS > 60.0, **MQ < 50.0**, **MQRankSum < -2.5**, **QD < 5.0**, **ReadPosRankSum < -2.5** and SOR > 3.0 (thresholds deviating from default in bold).

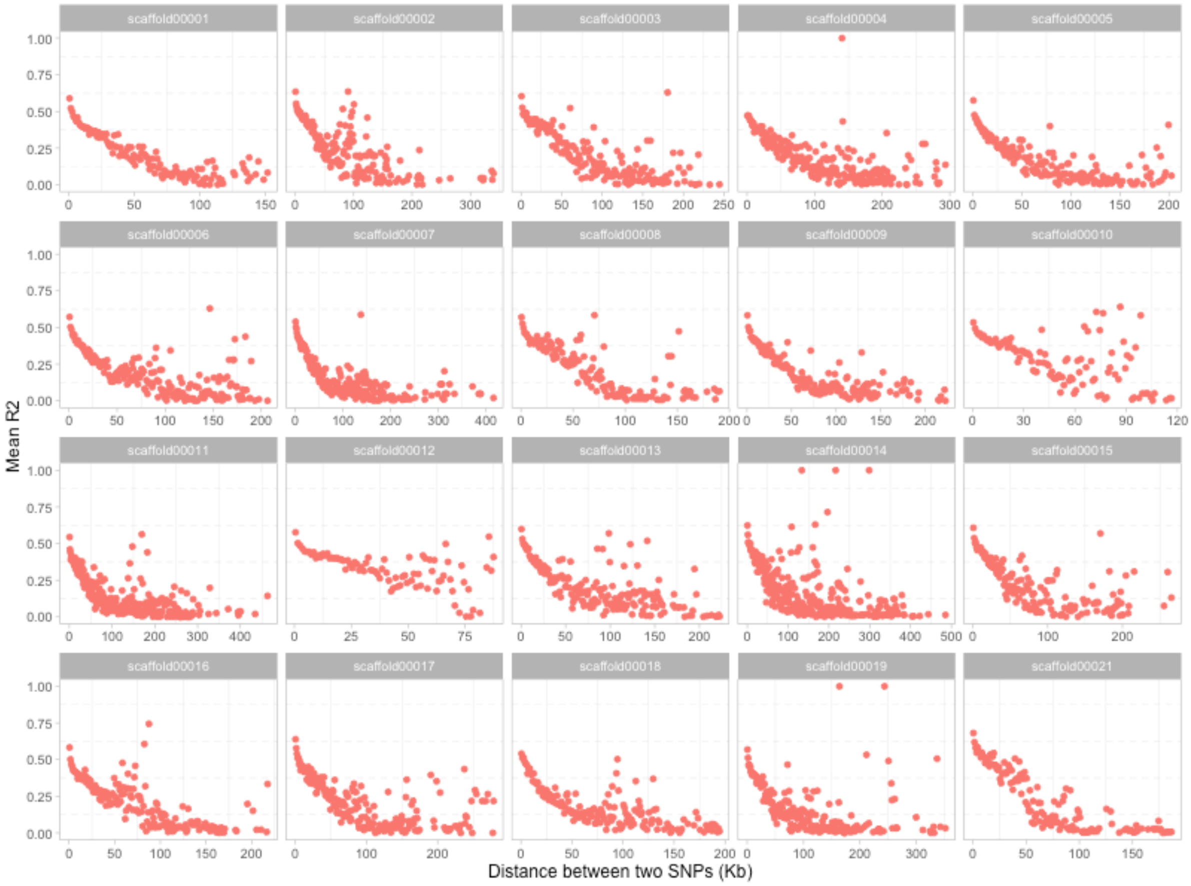

Figure S12. ***Linkage decay calculated for the 20 largest scaffolds in the genome assembly of great gerbil.***

For all scaffolds, linkage decays around 100 kb.

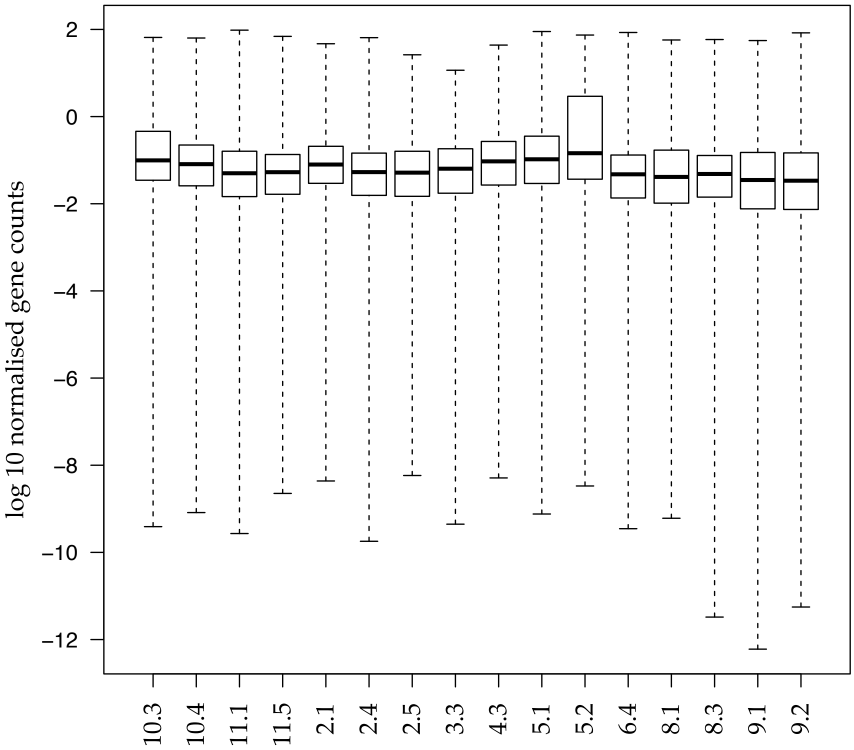

Figure S13. *Log10 of normalized gene counts for each of the 16 RNA libraries.*

Supplementary Tables

Table S1. ***Metadata on all great gerbils in the challenge experiment.***
Information on sex, body weight, temperature, dose of *Y. pestis* and days of disease onset, recovery and sampling are listed. Those individuals who also have RNA sequenced are marked. The animals' status at sampling is separated into those who died of the infection ('Moribund') and those that survived ('Recovered'). Individuals that did not show any signs of disease are marked "Healthy". Animal groups 2-6 and 8-11 were challenged with *Y. pestis*, while animal groups 7 and 12 are control animals injected with saline.

Animal group	*Ind.*	*Sex*	*Body weight (g)*	*Anus Temp. (°C)*	*Dose (×10^9 CFU)*	*Date of onset (p.i.)*	*Date of recovery (p.i.)*	*Date of dying* (p.i.)*	*Date of death (p.i.)*	*Date of samplin g (p.i.)*	*Animal status at sampling*
2	1	male	176	36.6	5.6	1		4		5	Moribund[a]
	2	male	219	36.2	5.6	1	7			22	Recovered
	3	female	187	37.8	5.6	1		3		3	Moribund
	4	male	176	37.8	5.6	1		4		5	Moribund[a]
	5	male	224	37.6	5.6	1		4		4	Moribund[a]
3	1	female	147	36.9	5.6	1		3		3	Moribund
	2	male	137	37.2	5.6	1	6			22	Recovered
	3	male	173	37.2	5.6	2	5			22	Recovered[a]
	4	female	130	38.3	5.6	1		4		5	Moribund
	5	female	126	36.8	5.6	1	6			22	Recovered
4	1	female	144	37.1	5.6	2		5		22	Moribund
	2	female	164	37.3	5.6	1		3		3	Moribund
	3	female	132	37.3	5.6	2		4		5	Moribund[a]
	4	male	174	38.0	5.6	1	7			22	Recovered
	5	male	169	37.1	5.6	1	6			22	Recovered
5	1	female	144	37.3	5.6	1		3		3	Moribund[a]
	2	female	167	37.6	5.6	-	-	-		3	Healthy[a]
	3	male	161	37.1	5.6	2	6			22	Recovered
	4	female	137	38.7	5.6	1		4		5	Moribund
	5	male	151	38.0	5.6	1	5			22	Recovered
6	1	female	179	37.2	5.6	2			13	13	Dead
	2	male	169	36.0	5.6	2	5			22	Recovered
	3	female	143	38.0	5.6	1		4		4	Moribund
	4	female	141	37.3	5.6	1	7			22	Recovered[a]
	5	female	132	37.6	5.6	1		5		5	Moribund
8	1	female	126	36.8	5.6	1	4			22	Recovered[a]
	2	female	125	36.6	5.6	2	5			22	Recovered
	3	female	123	37.7	5.6	1		4		5	Moribund[a]
	4	female	123	37.9	5.6	1		3		3	Moribund
	5	male	159	37.7	5.6	2	7			22	Recovered
9	1	male	202	37.2	5.6	1	6			22	Recovered[a]
	2	female	152	37.7	5.6	-	-	-		22	Healthy[b]
	3	male	111	36.4	5.6	1	5			22	Recovered
	4	female	137	38.2	5.6	-	-	-		4	Healthy
	5	female	126	37.5	5.6	1		4		5	Moribund
10	1	female	122	38.4	5.6	1		5		5	Moribund
	2	male	151	37.6	5.6	1	5			22	Recovered
	3	male	214	37.7	5.6	1		3		3	Moribund[a]
	4	female	116	37.8	5.6	2	6			22	Recovered[a]
	5	female	118	38.2	5.6	-	-	-		3	Healthy
11	1	male	133	37.7	5.6	1	5			22	Recovered[a]
	2	female	162	37.9	5.6	-	-	-		4	Healthy
	3	male	126	38.1	5.6	2	5			22	Recovered
	4	female	111	37.8	5.6	1		4		5	Moribund
	5	female	145	38.0	5.6	1		4		4	Moribund[a]

7	1	female	145	37.7	*saline*	-	-	-		22	Healthy
	2	female	132	38.1	*saline*	-	-	-		22	Healthy
	3	female	142	35.7	*saline*	-	-	-		22	Healthy
	4	male	129	35.3	*saline*	-	-	-		22	Healthy
	5	male	148	37.0	*saline*	-	-	-		22	Healthy
12	1	female	224	36.6	*saline*	-	-	-		22	Healthy
	2	female	157	37.9	*saline*	-	-	-		22	Healthy
	3	male	203	38.6	*saline*	-	-	-		22	Healthy
	4	female	121	37.4	*saline*	-	-	-		22	Healthy

[a] DNA and RNA sequence libraries generated
[b] RNA library generated
*The day the animals were euthanized

Table S2. *Sex-biased outcome of the infection.*
A q-square test was run to test for sex-related results in the outcome of the plague infection based on the information on individuals extracted from Table S1 and summarized in the table below.

	Males	*Females*	*Total*
Healthy	0	5	5
Recovered	14	5	19
Dying (including dead individual at day 13 p.i.)	4	17	21
**Total**	18	27	45

X-squared = 16.157, df = 2, p-value = 0.0003101

Table S3. *Gerbil relatedness estimated by IBD calculations.*
Identity by descent (IBD) calculations based on the LD pruned set (--indeep-pairwise 100 10 0.1). The set contains 32 816 SNPs. The sample prefix letter stands for the moribund animals i.e. dead (D) and survived (S), and is followed by the group and individual number. The table is ordered by descending PI_HAT, which is a measure of overall IBD alleles (Unrelated individuals should have values close to 0). The first two columns detail the two individuals in the comparison (IID=Individual ID). The following three columns are the probability of the pair sharing 0, 1 or 2 alleles at any given site, respectively. Z0 should be ~ 1 for completely unrelated individuals. Based on the PI_HAT value a degree of relationship is estimated as well as the possible relationship for the individuals sequenced in this study. Determining the exact relationship is difficult, e.g. both parent-offspring and full-siblings is expected to have a PI_HAT of 0.5.

IID1	*IID2*	*Z0*	*Z1*	*Z2*	*PI_HAT*	*Degree of relationship*	*Possible relationship*
D4-3	S3-3	0.2361	0.433	0.3309	**0.5474**	First	Full siblings
D2-4	D4-3	0.2814	0.4561	0.2625	**0.4905**	First	Full siblings

D2-4	S3-3	0.3345	0.4383	0.2272	**0.4463**	First	Full siblings
D5-1	S5-3	0.3703	0.3919	0.2379	**0.4338**	First	Full siblings
D4-3	S6-4	0.5278	0.2938	0.1784	**0.3253**	First or second	Half-siblings or closer
S3-3	S6-4	0.5347	0.2851	0.1802	**0.3228**	First or second	Half-siblings or closer
D4-3	S5-3	0.5334	0.2912	0.1754	**0.321**	First or second	Half-siblings or closer
D2-1	S5-3	0.5558	0.2685	0.1757	**0.3099**	First or second	Half-siblings or closer
D2-1	D4-3	0.5712	0.2393	0.1895	**0.3092**	First or second	Half-siblings or closer
D8-3	S9-1	0.5809	0.2324	0.1868	**0.303**	First or second	Half-siblings or closer
S3-3	S5-3	0.561	0.2774	0.1616	**0.3003**	First or second	Half-siblings or closer
D2-4	S6-4	0.5749	0.2539	0.1712	**0.2981**	First or second	Half-siblings or closer
D2-4	S5-3	0.5859	0.2552	0.159	**0.2866**	First or second	Half-siblings or closer
D2-1	S3-3	0.634	0.1785	0.1875	**0.2768**	First or second	Half-siblings or closer
D10-3	S9-1	0.6465	0.1555	0.198	**0.2758**	First or second	Half-siblings or closer
D9-5	S9-1	0.632	0.1844	0.1835	**0.2758**	First or second	Half-siblings or closer
D2-5	S9-1	0.646	0.1567	0.1974	**0.2757**	First or second	Half-siblings or closer
D2-1	D2-5	0.6577	0.1334	0.2089	**0.2756**	First or second	Half-siblings or closer
D10-3	S9-3	0.6506	0.1519	0.1975	**0.2735**	First or second	Half-siblings or closer
D4-3	D5-1	0.6413	0.1708	0.188	**0.2733**	First or second	Half-siblings or closer
D2-1	D5-1	0.6353	0.1846	0.1801	**0.2724**	First or second	Half-siblings or closer
D10-3	D2-5	0.6453	0.1673	0.1874	**0.271**	First or second	Half-siblings or closer
D11-5	S11-1	0.6542	0.1504	0.1955	**0.2706**	First or second	Half-siblings or closer
D2-3	D2-5	0.6481	0.1637	0.1883	**0.2701**	First or second	Half-siblings or closer
D11-5	S10-5	0.6732	0.1151	0.2117	**0.2692**	First or second	Half-siblings or closer
D2-5	D9-5	0.6574	0.1482	0.1944	**0.2685**	First or second	Half-siblings or closer
D2-3	D5-1	0.6605	0.1428	0.1967	**0.2681**	First or second	Half-siblings or closer
S5-2	S9-1	0.6502	0.1657	0.1841	**0.267**	First or second	Half-siblings or closer
D9-5	S5-2	0.6685	0.1295	0.202	**0.2667**	First or second	Half-siblings or closer
D5-1	S3-3	0.642	0.1843	0.1737	**0.2659**	First or second	Half-siblings or closer
D8-3	D9-5	0.6731	0.1226	0.2042	**0.2656**	First or second	Half-siblings or closer
D2-5	S10-5	0.6578	0.154	0.1881	**0.2652**	First or second	Half-siblings or closer
D10-3	D8-3	0.6585	0.1528	0.1887	**0.2651**	First or second	Half-siblings or closer
D2-5	S5-2	0.6662	0.1376	0.1962	**0.265**	First or second	Half-siblings or closer
S9-1	S9-3	0.6633	0.1437	0.193	**0.2648**	First or second	Half-siblings or closer
D2-3	D9-5	0.6559	0.1595	0.1846	**0.2644**	First or second	Half-siblings or closer
D2-3	S9-1	0.6585	0.1551	0.1865	**0.264**	First or second	Half-siblings or closer
D10-3	D9-5	0.68	0.1145	0.2055	**0.2627**	First or second	Half-siblings or closer
D2-3	S5-2	0.6812	0.1137	0.2051	**0.262**	First or second	Half-siblings or closer
D2-1	D2-4	0.6679	0.141	0.1911	**0.2616**	First or second	Half-siblings or closer
D2-3	S9-3	0.6746	0.1274	0.1979	**0.2616**	First or second	Half-siblings or closer
D10-3	S5-2	0.6708	0.1381	0.1912	**0.2602**	First or second	Half-siblings or closer
D10-3	D2-3	0.6738	0.132	0.1941	**0.2601**	First or second	Half-siblings or closer
D11-5	D9-5	0.6827	0.115	0.2022	**0.2598**	First or second	Half-siblings or closer

D2-1	D2-3	0.6818	0.1176	0.2006	**0.2594**	First or second	Half-siblings or closer
D8-3	S5-2	0.6718	0.1387	0.1895	**0.2588**	First or second	Half-siblings or closer
D2-3	D4-3	0.6721	0.1389	0.189	**0.2584**	First or second	Half-siblings or closer
D2-5	D8-3	0.6753	0.1327	0.192	**0.2583**	First or second	Half-siblings or closer
D2-3	D8-3	0.6789	0.1267	0.1944	**0.2578**	First or second	Half-siblings or closer
D9-5	S10-5	0.6864	0.1119	0.2017	**0.2577**	First or second	Half-siblings or closer
D2-4	D2-5	0.6645	0.1564	0.1792	**0.2574**	First or second	Half-siblings or closer
D2-1	S9-1	0.6763	0.1353	0.1884	**0.256**	First or second	Half-siblings or closer
D9-5	S9-3	0.6802	0.1282	0.1916	**0.2557**	First or second	Half-siblings or closer
D11-5	D2-5	0.6871	0.1161	0.1968	**0.2549**	First or second	Half-siblings or closer
D2-4	D5-1	0.6623	0.1664	0.1713	**0.2545**	First or second	Half-siblings or closer
D8-3	S9-3	0.6802	0.131	0.1888	**0.2543**	First or second	Half-siblings or closer
D11-5	D8-3	0.694	0.1046	0.2014	**0.2537**	First or second	Half-siblings or closer
D11-5	S9-1	0.6815	0.1303	0.1882	**0.2534**	First or second	Half-siblings or closer
S10-5	S9-1	0.6729	0.1515	0.1756	**0.2513**	First or second	Half-siblings or closer
D9-5	S8-1	0.6888	0.1218	0.1894	**0.2503**	First or second	Half-siblings or closer
D10-3	S10-4	0.7031	0.0942	0.2027	**0.2498**	Second	Half-siblings
D2-5	D5-1	0.689	0.1226	0.1884	**0.2497**	Second	Half-siblings
D11-5	S5-2	0.7149	0.0709	0.2142	**0.2496**	Second	Half-siblings
D2-5	S5-3	0.6864	0.1279	0.1857	**0.2496**	Second	Half-siblings
D2-5	S9-3	0.6809	0.14	0.1791	**0.2491**	Second	Half-siblings
D10-3	D2-1	0.7089	0.0845	0.2066	**0.2488**	Second	Half-siblings
S5-2	S9-3	0.7002	0.1022	0.1977	**0.2488**	Second	Half-siblings
D4-3	S9-1	0.676	0.1506	0.1734	**0.2487**	Second	Half-siblings
D11-5	D2-3	0.6986	0.1057	0.1957	**0.2486**	Second	Half-siblings
D2-3	S10-5	0.6964	0.1101	0.1934	**0.2485**	Second	Half-siblings
S11-1	S9-1	0.6717	0.1608	0.1675	**0.2479**	Second	Half-siblings
D2-1	D9-5	0.6988	0.1069	0.1943	**0.2477**	Second	Half-siblings
D2-3	D2-4	0.6861	0.1323	0.1815	**0.2477**	Second	Half-siblings
D2-3	S5-3	0.6836	0.1381	0.1783	**0.2473**	Second	Half-siblings
D2-5	D4-3	0.6834	0.1385	0.1781	**0.2473**	Second	Half-siblings
S10-4	S5-2	0.7033	0.0991	0.1976	**0.2472**	Second	Half-siblings
D10-3	S10-5	0.6992	0.1078	0.193	**0.2469**	Second	Half-siblings
D2-3	S6-4	0.6835	0.1401	0.1764	**0.2465**	Second	Half-siblings
D5-1	D8-3	0.6876	0.1323	0.1801	**0.2462**	Second	Half-siblings
D4-3	S5-2	0.7068	0.0942	0.199	**0.2461**	Second	Half-siblings
D2-4	S9-1	0.6866	0.1349	0.1785	**0.2459**	Second	Half-siblings
D2-1	S5-2	0.7032	0.1035	0.1934	**0.2451**	Second	Half-siblings
D10-3	D2-4	0.6969	0.1162	0.1869	**0.245**	Second	Half-siblings
D2-4	S5-2	0.6897	0.1316	0.1788	**0.2446**	Second	Half-siblings
D2-3	S10-4	0.7045	0.1019	0.1935	**0.2445**	Second	Half-siblings
D8-3	S10-4	0.7105	0.0904	0.1991	**0.2443**	Second or Third	First-cousins or closer

S10-4	S9-1	0.6948	0.1217	0.1835	**0.2443**	Second or Third	First-cousins or closer
D11-5	S10-4	0.7033	0.1055	0.1912	**0.2439**	Second or Third	First-cousins or closer
D10-3	S11-1	0.6976	0.1178	0.1846	**0.2435**	Second or Third	First-cousins or closer
D2-1	D8-3	0.7076	0.0978	0.1946	**0.2435**	Second or Third	First-cousins or closer
D11-5	S9-3	0.7266	0.0602	0.2132	**0.2433**	Second or Third	First-cousins or closer
D2-5	S11-1	0.6951	0.1234	0.1816	**0.2433**	Second or Third	First-cousins or closer
S5-3	S9-1	0.6895	0.1344	0.1761	**0.2433**	Second or Third	First-cousins or closer
D2-3	S3-3	0.692	0.1304	0.1776	**0.2428**	Second or Third	First-cousins or closer
D8-3	S10-5	0.7182	0.08	0.2017	**0.2417**	Second or Third	First-cousins or closer
S10-5	S5-2	0.7235	0.0701	0.2063	**0.2414**	Second or Third	First-cousins or closer
D4-3	D9-5	0.7033	0.1115	0.1853	**0.241**	Second or Third	First-cousins or closer
D5-1	D9-5	0.7181	0.0822	0.1997	**0.2408**	Second or Third	First-cousins or closer
D5-1	S5-2	0.7152	0.0891	0.1958	**0.2403**	Second or Third	First-cousins or closer
D5-1	S9-1	0.6973	0.1267	0.1761	**0.2394**	Second or Third	First-cousins or closer
S10-4	S9-3	0.7154	0.0921	0.1925	**0.2385**	Second or Third	First-cousins or closer
D10-3	D11-5	0.7167	0.0916	0.1918	**0.2375**	Second or Third	First-cousins or closer
S3-3	S9-1	0.6987	0.1289	0.1724	**0.2369**	Second or Third	First-cousins or closer
D2-3	S11-1	0.7074	0.1124	0.1802	**0.2364**	Second or Third	First-cousins or closer
D4-3	S9-3	0.7036	0.1201	0.1763	**0.2363**	Second or Third	First-cousins or closer
D11-5	D4-3	0.7199	0.0882	0.1919	**0.236**	Second or Third	First-cousins or closer
D2-4	D9-5	0.7119	0.1052	0.1829	**0.2355**	Second or Third	First-cousins or closer
D2-4	D8-3	0.7103	0.1094	0.1802	**0.235**	Second or Third	First-cousins or closer
D10-3	S5-3	0.7061	0.1182	0.1757	**0.2348**	Second or Third	First-cousins or closer
S10-4	S10-5	0.7293	0.0727	0.198	**0.2343**	Second or Third	First-cousins or closer
D9-5	S10-4	0.7278	0.0761	0.1961	**0.2341**	Second or Third	First-cousins or closer
D2-5	S10-4	0.7326	0.0673	0.2001	**0.2337**	Second or Third	First-cousins or closer
S10-5	S9-3	0.739	0.0548	0.2063	**0.2337**	Second or Third	First-cousins or closer
D2-1	S10-4	0.7278	0.0773	0.1949	**0.2336**	Second or Third	First-cousins or closer
D10-3	D4-3	0.7061	0.1214	0.1724	**0.2331**	Second or Third	First-cousins or closer
D5-1	S6-4	0.7285	0.0787	0.1928	**0.2322**	Second or Third	First-cousins or closer
D8-3	S11-1	0.7115	0.1139	0.1746	**0.2315**	Second or Third	First-cousins or closer
D8-3	S8-1	0.7423	0.0524	0.2053	**0.2315**	Second or Third	First-cousins or closer
D2-4	S10-5	0.7155	0.1067	0.1778	**0.2311**	Second or Third	First-cousins or closer
D2-1	S9-3	0.7311	0.0759	0.193	**0.2309**	Second or Third	First-cousins or closer
D8-3	S5-3	0.7001	0.138	0.1619	**0.2309**	Second or Third	First-cousins or closer
D4-3	S10-4	0.7263	0.0861	0.1877	**0.2307**	Second or Third	First-cousins or closer
D2-5	S3-3	0.719	0.1013	0.1797	**0.2303**	Second or Third	First-cousins or closer
D11-5	D2-1	0.7394	0.0613	0.1993	**0.23**	Second or Third	First-cousins or closer
D4-3	D8-3	0.7188	0.1029	0.1782	**0.2297**	Second or Third	First-cousins or closer
D8-3	S6-4	0.7255	0.0895	0.185	**0.2297**	Second or Third	First-cousins or closer
D9-5	S5-3	0.7271	0.0877	0.1852	**0.2291**	Second or Third	First-cousins or closer
S6-4	S9-1	0.7168	0.1081	0.175	**0.2291**	Second or Third	First-cousins or closer

S11-1	S9-3	0.7206	0.1011	0.1783	**0.2288**	Second or Third	First-cousins or closer
D9-5	S11-1	0.7215	0.0998	0.1787	**0.2286**	Second or Third	First-cousins or closer
S10-5	S11-1	0.7233	0.0966	0.1801	**0.2284**	Second or Third	First-cousins or closer
D4-3	S10-5	0.7308	0.0833	0.1859	**0.2276**	Second or Third	First-cousins or closer
S11-1	S5-2	0.7314	0.0829	0.1858	**0.2272**	Second or Third	First-cousins or closer
D5-1	S9-3	0.7423	0.0617	0.1959	**0.2268**	Second or Third	First-cousins or closer
D11-5	D5-1	0.7491	0.0484	0.2025	**0.2267**	Second or Third	First-cousins or closer
D2-5	S6-4	0.7246	0.0978	0.1775	**0.2265**	Second or Third	First-cousins or closer
D10-3	S3-3	0.7271	0.093	0.1799	**0.2264**	Second or Third	First-cousins or closer
S5-3	S9-3	0.7218	0.1039	0.1743	**0.2263**	Second or Third	First-cousins or closer
D10-3	D5-1	0.7302	0.0878	0.1821	**0.2259**	Second or Third	First-cousins or closer
D2-1	S10-5	0.7556	0.0369	0.2074	**0.2259**	Second or Third	First-cousins or closer
D2-1	S6-4	0.7444	0.0599	0.1958	**0.2257**	Second or Third	First-cousins or closer
D2-4	S9-3	0.7293	0.09	0.1807	**0.2257**	Second or Third	First-cousins or closer
D2-1	S11-1	0.7372	0.0768	0.186	**0.2244**	Second or Third	First-cousins or closer
S5-2	S6-4	0.7525	0.0469	0.2005	**0.224**	Second or Third	First-cousins or closer
S5-2	S5-3	0.7295	0.0943	0.1762	**0.2233**	Second or Third	First-cousins or closer
S5-3	S6-4	0.7221	0.1095	0.1684	**0.2231**	Second or Third	First-cousins or closer
S8-1	S9-1	0.7409	0.0732	0.186	**0.2226**	Second or Third	First-cousins or closer
D11-5	D2-4	0.7411	0.0739	0.185	**0.222**	Second or Third	First-cousins or closer
D5-1	S10-5	0.7491	0.0594	0.1915	**0.2212**	Second or Third	First-cousins or closer
D9-5	S3-3	0.7388	0.0801	0.181	**0.2211**	Second or Third	First-cousins or closer
D5-1	S10-4	0.7457	0.069	0.1853	**0.2198**	Second or Third	First-cousins or closer
D10-3	S8-1	0.7587	0.0433	0.198	**0.2197**	Second or Third	First-cousins or closer
D9-5	S6-4	0.7492	0.0633	0.1875	**0.2191**	Second or Third	First-cousins or closer
D11-5	S3-3	0.7498	0.0652	0.185	**0.2176**	Second or Third	First-cousins or closer
D8-3	S3-3	0.7406	0.0835	0.1759	**0.2176**	Second or Third	First-cousins or closer
S3-3	S9-3	0.7491	0.0679	0.183	**0.217**	Second or Third	First-cousins or closer
D2-3	S8-1	0.767	0.0328	0.2003	**0.2166**	Second or Third	First-cousins or closer
S3-3	S5-2	0.7465	0.0742	0.1794	**0.2165**	Second or Third	First-cousins or closer
D2-4	S10-4	0.7541	0.0591	0.1869	**0.2164**	Second or Third	First-cousins or closer
D10-3	S6-4	0.742	0.085	0.1731	**0.2156**	Second or Third	First-cousins or closer
S10-4	S11-1	0.7562	0.0574	0.1865	**0.2152**	Second or Third	First-cousins or closer
D4-3	S11-1	0.7407	0.0886	0.1707	**0.215**	Second or Third	First-cousins or closer
S10-5	S3-3	0.7406	0.0888	0.1706	**0.215**	Second or Third	First-cousins or closer
S6-4	S9-3	0.7643	0.0424	0.1933	**0.2145**	Second or Third	First-cousins or closer
D11-5	S5-3	0.7556	0.061	0.1834	**0.2139**	Second or Third	First-cousins or closer
D2-5	S8-1	0.785	0.0035	0.2115	**0.2133**	Second or Third	First-cousins or closer
D2-4	S11-1	0.7499	0.0747	0.1754	**0.2128**	Second or Third	First-cousins or closer
D11-5	S6-4	0.7774	0.0197	0.2029	**0.2127**	Second or Third	First-cousins or closer
S8-1	S9-3	0.7902	0	0.2098	**0.2098**	Second or Third	First-cousins or closer
S5-2	S8-1	0.7902	0.0016	0.2082	**0.209**	Second or Third	First-cousins or closer

S10-5	S5-3	0.7593	0.067	0.1736	**0.2072**	Second or Third	First-cousins or closer
S10-4	S3-3	0.7599	0.0666	0.1735	**0.2068**	Second or Third	First-cousins or closer
S11-1	S3-3	0.755	0.078	0.167	**0.206**	Second or Third	First-cousins or closer
S10-4	S6-4	0.7777	0.0328	0.1894	**0.2059**	Second or Third	First-cousins or closer
S10-4	S5-3	0.7547	0.0789	0.1664	**0.2058**	Second or Third	First-cousins or closer
S10-4	S8-1	0.7907	0.0079	0.2014	**0.2054**	Second or Third	First-cousins or closer
S11-1	S5-3	0.7635	0.0625	0.1739	**0.2052**	Second or Third	First-cousins or closer
S10-5	S6-4	0.7875	0.017	0.1955	**0.204**	Second or Third	First-cousins or closer
S10-5	S8-1	0.7962	0	0.2038	**0.2038**	Second or Third	First-cousins or closer
D5-1	S11-1	0.776	0.0423	0.1817	**0.2029**	Second or Third	First-cousins or closer
D11-5	S8-1	0.8013	0	0.1987	**0.1987**	Second or Third	First-cousins or closer
D5-1	S8-1	0.8026	0	0.1974	**0.1974**	Second or Third	First-cousins or closer
D2-4	S8-1	0.7959	0.0176	0.1865	**0.1953**	Second or Third	First-cousins or closer
D4-3	S8-1	0.8027	0.0046	0.1928	**0.195**	Second or Third	First-cousins or closer
D2-1	S8-1	0.8065	0	0.1935	**0.1935**	Second or Third	First-cousins or closer
S6-4	S8-1	0.808	0	0.192	**0.192**	Second or Third	First-cousins or closer
S11-1	S6-4	0.7942	0.0304	0.1755	**0.1907**	Second or Third	First-cousins or closer
S11-1	S8-1	0.8163	0	0.1837	**0.1837**	Second or Third	First-cousins or closer
S5-3	S8-1	0.8183	0.0033	0.1784	**0.1801**	Second or Third	First-cousins or closer
S3-3	S8-1	0.8212	0	0.1788	**0.1788**	Second or Third	First-cousins or closer

Table S4. ***Identified iHS peaks ordered by log_{10} (p) iHS (pval).***
The table lists the 234 identified outlier peaks exceeding the significance threshold (log_{10} 1 x 10^{-6}). See supplementary spreadsheet.

Table S5. ***Identified xpEHH peaks ordered by log_{10} (p) xpEHH (pval).***
The table lists the 122 identified outlier peaks exceeding the significance threshold (log_{10} 1 x 10^{-6}). For the values in the xpEHH column, highly negative stats suggest selection has occurred in the dead, positive in the survivors. See supplementary spreadsheet.

Table S6. ***Identified scaffolds with 50 SNP window FST > 0.2.***

SNP nr. (50 SNP window, 25 SNP jumps)	*Scaffold*	*From position (bp)*	*To position (bp)*	*Window size (bp)*	F_{ST}
34875-34925	scaffold00006	1979299	2042478	63179	0.22457049
34900-34950	scaffold00006	2004720	2078494	73774	0.20596373
66850-66900	scaffold00011	3130146	3183866	53720	0.22257451
120050-120100	scaffold00022	5257452	5320377	62925	0.20113562
128375-128425	scaffold00024	2250559	2428369	177810	0.22176164
128400-128450	scaffold00024	2332205	2479389	147184	0.23254351
138600-138650	scaffold00026	4353419	4409131	55712	0.24032508
138625-138675	scaffold00026	4371894	4439427	67533	0.23863422

138650-138700	scaffold00026	4409131	4473363	64232	0.23028291
163350-163400	scaffold00032	3969376	4125148	155772	0.20671795
197500-197550	scaffold00040	4773552	4849848	76296	0.23753786
197525-197575	scaffold00040	4810708	4885921	75213	0.31544787
197550-197600	scaffold00040	4849848	4947308	97460	0.29765788
206100-206150	scaffold00043	4140963	4176986	36023	0.21762523
206125-206175	scaffold00043	4158799	4187034	28235	0.24235891
206375-206425	scaffold00043	4397061	4482358	85297	0.24429199
206400-206450	scaffold00043	4436625	4563983	127358	0.21143258
206425-206475	scaffold00043	4482358	4586136	103778	0.21180082
206450-206500	scaffold00043	4563983	4631141	67158	0.25438596
206475-206525	scaffold00043	4586136	4647254	61118	0.28849903
207025-207075	scaffold00043	5281713	5347643	65930	0.2067677
207050-207100	scaffold00043	5317408	5364890	47482	0.20012938
207175-207225	scaffold00043	5471012	5522692	51680	0.22789964
263125-263175	scaffold00059	1530570	1626487	95917	0.21418785
263150-263200	scaffold00059	1557387	1659249	101862	0.20988857
337575-337625	scaffold00082	2553200	2587924	34724	0.22960859
393475-393525	scaffold00100	3823916	3883484	59568	0.24294766
393500-393550	scaffold00100	3843561	3897560	53999	0.25878426
393525-393575	scaffold00100	3883484	3938864	55380	0.21745383
393550-393600	scaffold00100	3897560	3982966	85406	0.20285579
398675-398725	scaffold00102	1965563	2034213	68650	0.28592411
398700-398750	scaffold00102	2002640	2052694	50054	0.40280464
398725-398775	scaffold00102	2034213	2093449	59236	0.27284926
403425-403475	scaffold00105	666638	809356	142718	0.20861105
403450-403500	scaffold00105	738498	1315826	577328	0.2868407
403475-403525	scaffold00105	809356	1656638	847282	0.23588782
510700-510750	scaffold00154	1615151	1744939	129788	0.21721724
510725-510775	scaffold00154	1676942	1795161	118219	0.23435625
545975-546025	scaffold00171	2994718	3073675	78957	0.21582658
546000-546050	scaffold00171	3017785	3178286	160501	0.21173584
587800-587850	scaffold00195	1167418	1198422	31004	0.20052454
613500-613550	scaffold00212	48368	141889	93521	0.21155562
613525-613575	scaffold00212	65413	245258	179845	0.32032294
613550-613600	scaffold00212	141889	276696	134807	0.20524997
635350-635400	scaffold00225	1491037	1703759	212722	0.20897461
651000-651050	scaffold00237	837599	959269	121670	0.23262274
669125-669175	scaffold00251	48268	113940	65672	0.20250627
720725-720775	scaffold00291	407554	489513	81959	0.22113145
720750-720800	scaffold00291	467862	532006	64144	0.24081036
720775-720825	scaffold00291	489513	564543	75030	0.24647054

720800-720850	scaffold00291	532006	589688	57682	0.24007404
767175-767225	scaffold00334	1197353	1327744	130391	0.22454625
820200-820250	scaffold00397	1434534	1494207	59673	0.23929651
820225-820275	scaffold00397	1475599	1534221	58622	0.23699335
820250-820300	scaffold00397	1494207	1627064	132857	0.21113382
820275-820325	scaffold00397	1534221	1704557	170336	0.2168308
820300-820350	scaffold00397	1627064	1734348	107284	0.22617354
863650-863700	scaffold00466	619821	761126	141305	0.25766751
863675-863725	scaffold00466	668765	924245	255480	0.26369863
897650-897700	scaffold00527	1023110	1128900	105790	0.23317627
930750-930800	scaffold00615	387040	513480	126440	0.20947284
935250-935300	scaffold00624	376388	569386	192998	0.21483133
968625-968675	scaffold00729	17208	134569	117361	0.24338351
973625-973674	scaffold00747	424379	697205	272826	0.21893242
1089200-1089250	scaffold01719	10067	18113	8046	0.24034485
1089225-1089275	scaffold01719	14421	22286	7865	0.20595014

Table S7. ***Significantly enriched gene pathways identified for iHS outlier genes.***
The terms in bold and highlighted in green are the Gene Ontology (GO) term that defines the GO group (i.e. significant pathway) in the GO analysis performed with GlueGo in Cytoscape. See supplementary spreadsheet.

Table S8. ***Genes with overlapping genome scan outliers.***
The table lists the genes, full names, scaffold and number of outliers found within each gene.

Gene	***Full name***	***Scaffold***	***Number of outlier positions***	***Genome scan***
MAN2B1	Mannosidase Alpha Class 2B Member 1	scaffold00031	1	xpEHH
MRPL47	Mitochondrial Ribosomal Protein L47	scaffold00054	1	xpEHH
CXCL14	C-X-C Motif Chemokine Ligand 14	scaffold00061	1	xpEHH
TXLNA	Taxilin Alpha	scaffold00099	1	xpEHH
BPGM	Bisphosphoglycerate mutase	scaffold00376	1	xpEHH
OR13A1	Olfactory Receptor Family 13 Subfamily A Member 1	scaffold00548	1	xpEHH
AKR1A1	Aldo-Keto Reductase Family 1 Member A1	scaffold00569	1	xpEHH
RPN2	Ribophorin II	scaffold00006	6	F_{ST}
GHRH	Growth Hormone Releasing Hormone	scaffold00006	7	F_{ST}
TTI1	TELO2-interacting protein 1	scaffold00006	3	F_{ST}
DDX27	DEAD-Box Helicase 27	scaffold00006	4	F_{ST}

GRWD1	Glutamate-rich WD repeat-containing protein 1	scaffold00011	1	F_{ST}
FANCG	FA Complementation Group G	scaffold00011	1	F_{ST}
MLF1	Myeloid leukemia factor 1	scaffold00022	7	F_{ST}
AMDHD2	Amidohydrolase Domain Containing 2	scaffold00024	3	F_{ST}
EIF4A1	Eukaryotic initiation factor 4A-I	scaffold00026	5	F_{ST}
EEF1E1	Eukaryotic translation elongation factor 1 epsilon-1	scaffold00032	2	F_{ST}
KIAA0408	Uncharacterized protein coding gene KIAA0408	scaffold00043	1	F_{ST}
EPB41L2	Erythrocyte Membrane Protein Band 4.1 Like 2	scaffold00043	9	F_{ST}
CCN	Cellular Communication Network Factor 2	scaffold00043	1	F_{ST}
FAN1	FANCD2 And FANCI Associated Nuclease 1	scaffold00059	2	F_{ST}
MPHOSPH10	M-Phase Phosphoprotein 10	scaffold00059	1	F_{ST}
MCEE	Methylmalonyl-CoA Epimerase	scaffold00059	10	F_{ST}
PLEKHA3	Pleckstrin homology domain-containing A3	scaffold00082	4	F_{ST}
TTN	Titin	scaffold00082	23	F_{ST}
DFNB59	Pejvakin	scaffold00082	1	F_{ST}
FKBP7	FKBP Prolyl Isomerase 7	scaffold00082	1	F_{ST}
MAP3K7	Mitogen-activated protein kinase kinase kinase 7	scaffold00105	1	F_{ST}
TMC3	Transmembrane channel-like protein 3	scaffold00195	1	F_{ST}
RPL37A-PS1	Putative 60S ribosomal protein L37a	scaffold00212	6	F_{ST}
ANKRD26	Ankyrin Repeat Domain 26	scaffold00225	1	F_{ST}
RAD52	RAD52 Homolog, DNA Repair Protein	scaffold00225	1	F_{ST}
WNK1	WNK Lysine Deficient Protein Kinase 1	scaffold00225	3	F_{ST}
IL17RA	Interleukin-17 receptor A	scaffold00225	1	F_{ST}
C1ORF43	Chromosome 1 Open Reading Frame 43	scaffold00291	8	F_{ST}
RIC1	RAB6A-GEF complex partner protein 1	scaffold00334	7	F_{ST}
TMPRSS11A	Transmembrane protease serine 11A	scaffold00466	8	F_{ST}
CELF4	CUGBP Elav-like family member 4	scaffold00466	31	F_{ST}
TMPRSS11E	Transmembrane protease serine 11E	scaffold00466	3	F_{ST}
KARS	Lysine--tRNA ligase	scaffold00005	1	iHS
TOPAZ1	Testis- and ovary-specific PAZ domain-containing protein 1	scaffold00009	1	iHS
RGS10	Regulator of G-protein signaling 10	scaffold00011	1	iHS
DAB2	Disabled homolog 2	scaffold00012	1	iHS
KCNIP1	Potassium Voltage-Gated Channel Interacting Protein 1	scaffold00024	1	iHS

RPL37A	60S ribosomal protein L37a	scaffold00094	1	iHS
ZC3H12C	Zinc Finger CCCH-Type Containing 12C	scaffold00099	1	iHS
ACSM4	Acyl-CoA Synthetase Medium Chain Family Member 4	scaffold00142	1	iHS
IFT81	Intraflagellar transport 81	scaffold00198	1	iHS
RPS26	40S ribosomal protein S26	scaffold00198	1	iHS
G6PC	Glucose-6-phosphatase	scaffold00209	1	iHS
DDT	D-Dopachrome Tautomerase	scaffold00217	1	iHS
CROT	Carnitine O-Octanoyltransferase	scaffold00240	1	iHS
PRL8A4	Prolactin-8A4	scaffold00331	1	iHS
NLRP1B	NLR Family Pyrin Domain Containing 1	scaffold00346	1	iHS
PCDHB18	Protocadherin Beta 18	scaffold00422	1	iHS
BBS10	Bardet-Biedl syndrome 10	scaffold00423	1	iHS
ROS1	ROS Proto-Oncogene 1, Receptor Tyrosine Kinase	scaffold00551	1	iHS

Table S9. ***Significantly enriched gene pathways identified for xpEHH outlier genes.***
The terms in bold and highlighted in green are the Gene Ontology (GO) term that defines the GO group (i.e. significant pathway) in the GO analysis performed with ClueGo in Cytoscape. See supplementary spreadsheet.

Table S10. ***Significantly enriched gene pathways identified for F_{ST} outlier genes.***
The terms in bold and highlighted in green are the Gene Ontology (GO) term that defines the GO group (i.e. significant pathway) in the GO analysis performed with GlueGo in Cytoscape. See supplementary spreadsheet.

Table S11. ***Genes found in both xpEHH and F_{ST} genome scans.***
The table lists the 24 candidate genes identified in both analyses.

Gene	***Full name***	***Scaffold***
GAPDH	Glyceraldehyde-3-phosphate dehydrogenase	scaffold00006
PPIA	Peptidyl-prolyl cis-trans isomerase A	scaffold00022
RPL21	60S ribosomal protein L21	scaffold00022
RPL29	60S ribosomal protein L29	scaffold00022
RPS6	40S ribosomal protein S6	scaffold00022
HMGB2	High mobility group protein B2	scaffold00032
AIMP1	Aminoacyl tRNA synthase complex-interacting multifunctional protein 1	scaffold00043
ECHDC1	Ethylmalonyl-CoA decarboxylase	scaffold00043
KIAA0408	Uncharacterized protein KIAA0408	scaffold00043
LEG1	Protein LEG1 homolog	scaffold00043
RPL7A	60S ribosomal protein L7a	scaffold00043
SOGA3	SOGA Family Member 3	scaffold00043
THEMIS	Thymocyte Selection Associated	scaffold00043
TMEM200A	Transmembrane Protein 200A	scaffold00043
UBE2E1	Ubiquitin-conjugating enzyme E2 E1	scaffold00043

VDAC1	Voltage-dependent anion-selective channel protein 1	scaffold00043
RPSA	40S ribosomal protein SA	scaffold00059
HNRNPH2	Heterogeneous nuclear ribonucleoprotein H2	scaffold00100
FTSJ2 (MRM2)	Mitochondrial rRNA methyltransferase 2	scaffold00102
RPS23	40S ribosomal protein S23	scaffold00171
BCL2L13	Bcl-2-like protein 13	scaffold00225
BID	BH3-interacting domain death agonist	scaffold00225
MICAL3	Microtubule Associated Monooxygenase, Calponin And LIM Domain Containing 3	scaffold00225
RPL28	60S ribosomal protein L28	scaffold00237

Table S12. ***Genes differentially expressed between dead and survivors.***
List of differentially expressed genes when comparing moribund gerbils to survivors. Thus, positive values of the log fold change (logFC) are to be interpreted as the level of upregulation of said gene in the moribund gerbils while negative values are correspondingly the level of downregulation in moribund gerbils. See supplementary spreadsheet.

Table S13. ***Enriched pathways for all DE genes.***
The terms in bold are the Gene Ontology (GO) term that defines the GO group (i.e. significant pathway) in the GO analysis performed with ClueGo in Cytoscape. See supplementary spreadsheet.
The GO analyses revealed 22 significantly enriched pathways in the full gene set and showed that the gerbil immune system is highly activated during infection with plague. Several prominent inflammatory pathways are enriched and include both cellular and humoral immunity such as *acute inflammatory response* ($p = 1.58 \times 10^{-9}$), *leukocyte mediated immunity* ($p = 4.93 \times 10^{-12}$) and *humoral immune response* ($p = 3.50 \times 10^{-5}$).

Table S14. ***Enriched pathways for DE genes upregulated in dead.***
The terms in bold are the Gene Ontology (GO) term that defines the GO group (i.e. significant pathway) in the GO analysis performed with ClueGo in Cytoscape. See supplementary spreadsheet.
Most of the enriched pathways reported for all 146 genes are those reported for just the upregulated genes.

Table S15. ***Enriched pathways for DE genes downregulated in dead.***
The terms in bold are the Gene Ontology (GO) term that defines the GO group (i.e. significant pathway) in the GO analysis performed with ClueGo in Cytoscape. See supplementary spreadsheet.
Three significantly enriched pathways were reported for the 22 downregulated genes; *positive regulation of IL-6 production* ($p = 3.29 \times 10^{-4}$), *antibiotic catabolic process* ($p = 2.29 \times 10^{-4}$) and *positive regulation of G1/S transition of mitotic cell cycle* ($p = 1.03\ 10^{-3}$).

Table S16. ***Genes identified in both DE and genome scan analyses.***
The table lists the 22 genes identified as differentially expressed between survivors and moribund that was also identified in one or more genome scan analyses.

Gene	*Full name*	*Scaffold*	*Regulation in moribund*	*Genome scan*
SYCP3	Synaptonemal complex protein 3	scaffold00003	Downregulated	iHS
SDC4	Syndecan-4	scaffold00006	Upregulated	fst
MMP9	Matrix metalloproteinase-9	scaffold00006	Upregulated	fst
FGF21	Fibroblast growth factor 21	scaffold00011	Upregulated	fst
RASIP1	Ras-interacting protein 1	scaffold00011	Upregulated	fst
PRSS29	Serine protease 29	scaffold00024	Upregulated	fst, iHS
MASTIN	Mastin	scaffold00024	Upregulated	fst
NPW	Neuropeptide W	scaffold00024	Upregulated	fst
NAA60	N-Alpha-Acetyltransferase 60, NatF Catalytic Subunit	scaffold00024	Upregulated	iHS
SERPINA1	1-antitrypsin	scaffold00030	Upregulated	xpEHH, iHS
HMGB2	High mobility group protein B2	scaffold00032	Downregulated	fst
CLK4	CDC Like Kinase 4	scaffold00039	Downregulated	xpEHH
SPP1	Secreted Phosphoprotein 1	scaffold00056	Upregulated	xpEHH
PLAC8	Placenta Associated 8	scaffold00056	Upregulated	iHS
RPSA	40S ribosomal protein SA	scaffold00059	Downregulated	fst, xpEHH, iHS
MRC2	Mannose Receptor C Type 2	scaffold00104	Upregulated	iHS
BST1	Bone Marrow Stromal Cell Antigen 1	scaffold00109	Upregulated	xpEHH
RPL27	60S ribosomal protein L27	scaffold00180	Downregulated	xpEHH, iHS
GRN	Granulin Precursor	scaffold00209	Upregulated	iHS
IGFBP2	Insulin-like growth factor-binding protein 2	scaffold00245	Upregulated	xpEHH
PCP4	Purkinje cell protein 4	scaffold00271	Upregulated	iHS
PSD4	PH and SEC7 domain-containing protein 4	scaffold00279	Upregulated	iHS

Table S17. ***Repeats and transposable elements in the great gerbil genome assembly.***
The table consists of results reported by RepeatMasker (v4.0.6), RM database version 20150807.

	Number of elements	*Length occupied (bp)*	*Percentage of sequence*
**SINEs**	1 277 050	175 362 841	7.38 %
Alu/B1	486 842	56 246 833	2.37 %
B2-B4	625 530	102 305 618	4.31 %
IDs	59 572	4 167 984	0.18 %
MIRs	102 608	12 373 453	0.52 %
**LINEs**	653 666	373 716 365	15.73 %
LINE1	583 914	362 038 815	15.24 %
LINE2	55 464	9 704 805	0.41 %
L3/CR1	10 407	1 462 460	0.06 %
**LTR elements**	495 872	159 611 732	6.72 %

ERVL	78 593	23 061 183	0.97 %
ERVL-MaLRs	286 034	84 110 794	3.54 %
ERV_classI	24 396	8 833 851	0.37 %
ERV_classII	102 289	42 656 671	1.80 %
**DNA elements**	140 568	28 150 956	1.18 %
hAT-Charlie	91 613	17 850 598	0.75 %
TcMar-Tigger	22 826	5 273 523	0.22 %
**Unclassified**	14 666	7 184 112	0.30 %
**Total interspersed repeats**		744 026 006	31.31 %
**Small RNA**	12 499	1 019 847	0.04 %
**Satellites**	2 187	361 666	0.02 %
**Simple repeats**	1 100 624	54 635 296	2.30 %
**Low complexity**	168 239	11 669 447	0.49 %

References

1. Lu, H. *et al.* Chinese deserts and sand fields in Last Glacial Maximum and Holocene Optimum. *Chin. Sci. Bull.* **58,** 2775–2783 (2013).
2. Star, B. & Spencer, H. G. Effects of genetic drift and gene flow on the selective maintenance of genetic variation. *Genetics* **194,** 235–244 (2013).
3. Randall, J. A. Flexible social structure of a desert rodent, *Rhombomys opimus*: philopatry, kinship, and ecological constraints. *Behavioral Ecology* **16,** 961–973 (2005).
4. Wang, Y., Liu, W., Wang, G. M., Zhong, W. & Wan, X. Genetic consequences of group living in Mongolian gerbils. *J. Hered.* **102,** 554–561 (2011).
5. Fothergill-Gilmore, L. A. & Watson, H. C. The phosphoglycerate mutases. *Adv. Enzymol. Relat. Areas Mol. Biol.* **62,** 227–313 (1989).
6. Hromas, R. *et al.* Cloning of BRAK, a novel divergent CXC chemokine preferentially expressed in normal versus malignant cells. *Biochem. Biophys. Res. Commun.* **255,** 703–706 (1999).
7. Shurin, G. V. *et al.* Loss of new chemokine CXCL14 in tumor tissue is associated with low infiltration by dendritic cells (DC), while restoration of human CXCL14 expression in tumor cells causes attraction of DC both *in vitro* and *in vivo*. *J. Immunol.* **174,** 5490–5498 (2005).
8. Koyanagi, M. *et al.* ZFAT expression in B and T lymphocytes and identification of ZFAT-regulated genes. *Genomics* **91,** 451–457 (2008).
9. Doi, K. *et al.* ZFAT plays critical roles in peripheral T cell homeostasis and its T cell receptor-mediated response. *Biochem. Biophys. Res. Commun.* **425,** 107–112 (2012).
10. Fujimoto, T. *et al.* ZFAT is an antiapoptotic molecule and critical for cell survival in MOLT-4 cells. *FEBS Letters* **583,** 568–572 (2009).
11. Ishikura, S. *et al.* Zfat-deficient $CD4^+$ $CD8^+$ double-positive thymocytes are susceptible to apoptosis with deregulated activation of p38 and JNK. *J. Cell. Biochem.* **116,** 149–157 (2015).
12. Shirasawa, S. *et al.* SNPs in the promoter of a B cell-specific antisense transcript, SAS-ZFAT, determine susceptibility to autoimmune thyroid disease. *Hum. Mol. Genet.* **13,** 2221–2231 (2004).
13. Inoue, N. *et al.* Associations between autoimmune thyroid disease prognosis and functional polymorphisms of susceptibility genes, CTLA4, PTPN22, CD40, FCRL3, and ZFAT, previously revealed in genome-wide association studies. *J. Clin. Immunol.* **32,** 1243–1252 (2012).
14. Ji, H. Y. *et al.* A genome-wide association analysis for susceptibility of pigs to enterotoxigenic *Escherichia coli* F41. *Animal* **10,** 1602–1608 (2016).
15. Williams, J. H. *et al.* HLS7, a hemopoietic lineage switch gene homologous to the leukemia-inducing gene MLF1. *EMBO J.* **18,** 5559–5566 (1999).
16. Matsumoto, N. *et al.* Elevated MLF1 expression correlates with malignant progression from myelodysplastic syndrome. *Leukemia* **14,** 1757–1765 (2000).
17. Cornelis, G. R. Yersinia type III secretion: send in the effectors. *J. Cell Biol.* **158,** 401–408 (2002).

18. Mukherjee, S. Yersinia YopJ Acetylates and Inhibits Kinase Activation by Blocking Phosphorylation. *Science* **312,** 1211–1214 (2006).
19. Sweet, C. R., Conlon, J., Golenbock, D. T., Goguen, J. & Silverman, N. YopJ targets TRAF proteins to inhibit TLR-mediated NF-kappaB, MAPK and IRF3 signal transduction. *Cellular Microbiology* **9,** 2700–2715 (2007).
20. Shao, F. Biochemical functions of Yersinia type III effectors. *Current Opinion in Microbiology* **11,** 21–29 (2008).
21. Kerschen, E. J., Cohen, D. A., Kaplan, A. M. & Straley, S. C. The Plague Virulence Protein YopM Targets the Innate Immune Response by Causing a Global Depletion of NK Cells. *Infection and Immunity* **72,** 4589–4602 (2004).
22. Kulikov, A. V. *et al.* Cytochrome c: the Achilles' heel in apoptosis. *Cell. Mol. Life Sci.* **69,** 1787–1797 (2012).
23. Shoshan-Barmatz, V., Maldonado, E. N. & Krelin, Y. VDAC1 at the crossroads of cell metabolism, apoptosis and cell stress. *Cell Stress* **1,** 11–36 (2017).
24. Praefcke, G. J. K. Regulation of innate immune functions by guanylate-binding proteins. *Int. J. Med. Microbiol.* **308,** 237–245 (2018).
25. Pilla-Moffett, D., Barber, M. F., Taylor, G. A. & Coers, J. Interferon-Inducible GTPases in Host Resistance, Inflammation and Disease. *J. Mol. Biol.* **428,** 3495–3513 (2016).
26. Ogata, H. *et al.* The toll-like receptor protein RP105 regulates lipopolysaccharide signaling in B cells. *J. Exp. Med.* **192,** 23–29 (2000).
27. Kimoto, M., Nagasawa, K. & Miyake, K. Role of TLR4/MD-2 and RP105/MD-1 in innate recognition of lipopolysaccharide. *Scand J Infect Dis* **35,** 568–572 (2003).
28. Divanovic, S. *et al.* Negative regulation of Toll-like receptor 4 signaling by the Toll-like receptor homolog RP105. *Nat. Immunol.* **6,** 571–578 (2005).
29. Liu, B. *et al.* Involvement of RP105 and toll-like receptors in the activation of mouse peritoneal macrophages by *Staphylococcus aureus*. *Scand J Immunol* **78,** 8–16 (2013).
30. Aepfelbacher, M. & Heesemann, J. Modulation of Rho GTPases and the actin cytoskeleton by Yersinia outer proteins (Yops). *Int. J. Med. Microbiol.* **291,** 269–276 (2001).
31. Velan, B. *et al.* Discordance in the effects of *Yersinia pestis* on the dendritic cell functions manifested by induction of maturation and paralysis of migration. *Infection and Immunity* **74,** 6365–6376 (2006).
32. Hall, A. Rho GTPases and the actin cytoskeleton. *Science* **279,** 509–514 (1998).
33. Lutz, S. *et al.* Structure of Galphaq-p63RhoGEF-RhoA complex reveals a pathway for the activation of RhoA by GPCRs. *Science* **318,** 1923–1927 (2007).
34. Swenson-Fields, K. I. *et al.* MLK3 limits activated Galphaq signaling to Rho by binding to p63RhoGEF. *Mol. Cell* **32,** 43–56 (2008).
35. Momotani, K. *et al.* p63RhoGEF Couples G αq/11-Mediated Signaling to Ca 2+Sensitization of Vascular Smooth Muscle Contractility. *Circulation Research* **109,** 993–1002 (2011).
